# Synergistic and potential antifungal properties of tailored, one pot multicomponent monoterpenes co-delivered with fluconazole encapsulated nanostructure lipid carrier

**DOI:** 10.1038/s41598-024-63149-x

**Published:** 2024-06-22

**Authors:** Ibrahim Taha Radwan, Ibrahim M. El-Sherbiny, Nadia Hanafy Metwally

**Affiliations:** 1 Supplementary General Sciences Department, Faculty of Oral and Dental Medicine, Future University in Egypt, Cairo, 11835 Egypt; 2 Center for Materials Science (CMS), Zewail City of Science and Technology, 6th of October, Giza, 12578 Egypt; 3 Chemistry Department, Faculty of Science, Cairo University, Giza, 12613 Egypt

**Keywords:** Biochemistry, Biological techniques

## Abstract

Frequent and variant infections are caused by the virtue of opportunistic fungi pathogens. Candidiasis, aspergillosis, and mucormycosis are pathogenic microorganisms that give rise to vast fungal diseases that alternate between moderate to fatal in severity. The use of fluconazole as an antifungal drug was limited due to the acquired resistance in some types of Candida and other fungal species. This study aims to consolidate fluconazole’s biological effectiveness against several pathogenic fungi. Six active monoterpenes (MTs) of carvacrol, linalool, geraniol, *α*-terpinene, citronellal, and nerolidol were selected and encapsulated in nanostructure lipid carrier (NLC) with (NLC-Flu-MTs) and/without (NLC-MTs) fluconazole in one nanoformulation to determine if they will act synergistically or not? The synthesized nanoformulation NLC-Flu-MTs and NLC-MTs exhibited very good particle size of 144.5 nm and 138.6 nm for size and zeta potential values of (− 23.5 mV) and (− 20.3 mV), respectively. Transmission electron microscope investigation confirmed that the synthesized NLCs have regular and spherical shape. The abundance and concentration of the six released monoterpenes were determined, as a novel approach, using GC–MS with very good results and validity. In-vitro antifungal screening was done before and after nano co-delivery against seven pathogenic, and aggressive fungi of *Candida tropicalis, Candida krusei, Candida glabrata, Geotrichum Candidum*, *Candidaalbicans*, *Aspergillus Niger, and mucor circinelloides.* Inhibition Zone diameter (IZD) and the minimum inhibitory concentration (MIC) were measured. Nanoformulations NLC-Flu-MTs and NLC-MTs manifested potential and unique biological susceptibility against all the tested microorganisms with reduced (MIC) values, especially against *Candida Tropicalis* (MIC = 0.97 µg/ml) which represents 16-fold of the value shown by NLC-MTs (MIC = 15.6 µg/ml) and 64-fold of fluconazole free before nanoformulation (MIC = 62.5 µg/ml). The efficiency of nanomaterials, particularly NLC-Flu-MTs, has become evident in the diminishing value of MIC which affirmed the synergism between fluconazole and the other six monoterpenes.

## Introduction

A fungus is one of the most diverse organisms in the world. Thus it’s essential to synthesize new fungicidal compounds with high selectivity for fungal receptors and low affinity for human receptors^1,2^. Recently, fungal infections have increased and are responsible for 1–2 million fatalities annually^3^. *Aspergillus* and *Candida* species are responsible for about 90% of deaths (∼ 90%), but other species like *Candida tropicalis*, *Candida glabrata*, *Candida parapsilosis*, *Candida krusei*, and *Candida albicans* enhanced rate of mortality (75%)^4^. *Candida* species have emerged as the most common cause of systemic fungal infections in hospitals worldwide. Although *Candida albicans* is the most frequently isolated yeast, non-*albican Candida* species including *C. lipolytica, C. parapsilosis*, and *C. tropicalis* are increasingly found^5^.

Azoles have a wide range of biological activities, including anticancer, anti-diabetic, anti-inflammatory and a variety of other medicinal purposes, especially as antimicrobial and antifungal agents^6–10^. Triazoles are an important class of azoles like 1,2,3-triazole derivatives, which in the last few years have been generated and evaluated for their antifungal activity, with some potential activity against various fungi. Effect of the nitrogen heteroatoms in the triazole ring system on the reactivity of lead compound target medication pharmacokinetics and metabolism are affected by the interactions of lead compounds with several target inhibitors^11^. Important approved triazole antifungals include fluconazole (Diflucan™), voriconazole (Vfend™) and posaconazole (Noxa-fil™), which appear to have expanded antifungal activity. Ketoconazole was the first drug used for oral systemic fungal infections. Subsequently, ongoing research attempted to design broad-spectrum antifungal agents, the triazole derivatives fluconazole and itraconazole were prepared and succeeded in achieving the purpose with an improved safety profile.

**Figure 1:**
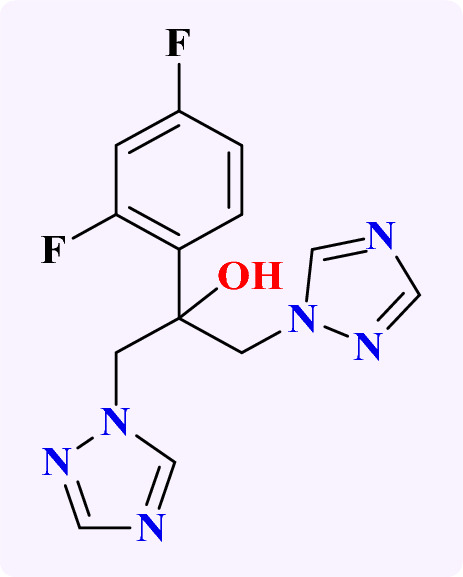
The structure of fluconazole.

Fluconazole (Fig. 1) is one of the most recommended and prescribed antifungal agents for Candida infections. Like other triazole antifungals, fluconazole targets the ergosterol biosynthetic process in fungi, especially, Candida by inhibiting the cytochrome P450-dependent enzyme lanosterol 14-*α*-demethylase encoded by ERG11. Here, the free nitrogen atom in the triazole binds to the iron atom in the heme group of the enzyme^12^. As a result, oxygen is no longer activated, the demethylation of lanosterol is inhibited, and the ergosterol biosynthesis process is inhibited^13^. Since ergosterol is a substantial ingredient of the fungal cell membrane, this change is very detrimental to the accumulation of methylated sterols in the fungal cell membrane, and results in cell growth arrest^14^.

Fluconazole is fungistatic (drugs inhibit fungal growth) rather than fungicidal (drugs kill fungal pathogens). Today increase in fluconazole resistance is one of the crucial problems in long-term fungal infections. Resistance to fluconazole may result from different mechanisms, including the successive secretion of *α*-demethylase in ergosterol biosynthesis which mean ERG11 overexpression, which led to successive synthesis of Lanosterol 14*α*-demethylase that make the fluconazole need to be increased to compensate the excess of Lanosterol 14*α*-demethylase secreted (as the triazole in fluconazole ring binds to the heme group of the enzyme) made fluconazole less active and more resistant^15–18^ that typically associated with widespread and overuse of fluconazole as prophylaxis medications^15^. About 65% of fluconazole-resistant candida species mutations in the ERG11 gene expression^19,20^. Another factor contributing to fluconazole-resistant strains is the widespread involvement of secreted aspartyl proteinases (SAPs), which are considered potential factors affecting the virulence and extracellular proteolytic activity of the candida strains. SAPs also play an important role in colony formation, adhesion to the host cells via penetration, and tissue attack by destroying the surface, and cell membrane of the host cells^21^. A Family of SAP genes has been presented all of which were effective against the *C. albicans* pathogenicity^22,23^. Many trials have been made to get rid of fluconazole resistance and to restore its reactivity by converting fluconazole to its nanoform for example, fluconazole combined with curcumin and/or silver nanoparticle^24^ loading fluconazole on the surface of ZnO nps^25^, fluconazole conjugated with Cu–Ag and/ or ZnO nps^26^, fluconazole coupled with Au NPs^27^ and many other successive loading attempts using gel and lipid nps^28,29^.

Nystatin is one of the drugs combined with fluconazole and used for the treatment of recurrent vulvovaginal candidiasis (VVC) caused by *C. albicans*. Nystatin belongs to the polyenes family, which changes in the cell membrane permeability through binding to the sterols and causes the substantial contents of the fungus intracellular to be removed^25^. The utility of natural products (Terpenes) is one of the most attractive areas in medicinal and herbal chemistry. In one of the attempts to examine the antifungal efficacy of carvacrol, thymol, eugenol, and menthol terpenes as alternative agents to control fungus growth in the food industry, it was found that all the used Terpenes have a positive effect on all fungus strains with good minimal inhibitory concentrations ranged between 100 and 300 µg/ml and the activity order was thymol, carvacrol, eugenol and menthol^30^. Another study aimed to evaluate carvacrol and thymol as postharvest pathogens of *Botrytis cinerea* retardant, which causes stem and fruit rot during pre-harvesting. The results of this study indicated very strong antifungal activity, causing obvious changes in the morphology of the *B. cinerea* hyphae^31^. It has been reported that the using of carvacrol as an antifungal agent could be effective reasoning that due to, carvacrol could induce apoptosis in *Candida albicans* via different mechanisms of the total active and the mitochondrial reactive oxygen species (ROS) levels were exalted, and each of the mitochondrial transmembrane potential and morphology were changed. In addition, the cytosolic and mitochondrial calcium standards were also elevated^32^. Linalool is a very important member of monoterpenes, and has a major role as an antifungal agent, especially *Candida albicans*. The inhibitory effect of linalool against *C. albicans's* filamentous growth and biofilm formation was studied; the outcomes published attributed to the great effect of linalool on both fungal growth and biofilm formation. Additionally, gene expression estimated by RT-PCR confirmed the downregulation of HWP1 and ALS3. Like the adhesion genes (HWP1 and ALS3), the expression levels of long-term hyphae maintenance were also suppressed^33^ and some other studies confirmed that linalool is a very good antifungal agent^34,35^. The antifungal activity of geraniol and citronellol (alcoholic form of citronellal) was studied against the most common fungus causing dermatophytosis, trichophyton rubrum. One of the main outcomes of this study is, that the minimal inhibitory concentration is between 16 and 256 µg/ml for geraniol and 8–1024 µg/ml for citronellol against different strains of *T. rubrum*. In addition, using MIC and twice MIC could inhibit the mycelia growth and conidia germination, and fungal growth on nail fragments, and using half the MIC could cause wide, short, and crooked hyphae in *T. rubrum* morphology^36^. Not only do single citronellal or even citronellal have great antifungal but the citronella extracts enriched with both have also very good antifungal activities^37^. Peruvian piper is one of the most important plant extract enriched with natural terpenes like alpha terpinene and nerolidol, the antifungal study of this plant extract against the phytopathogenic *Aspergillus niger*, *Botrytis cinerea*, and *Alternaria alternate* revealed a very good activity those single compounds especially, with the fungus *Botrytis cinerea*^38^.

Frequent and variant infections caused by different fungal species in recent years. It poses a severe threat to humanity, especially people who undergo surgical interventions such as organ transplantation, which require taking immunosuppressants to make the body accept the transplanted organ. And those who suffer from autoimmune diseases, resulting in high incidence lethal rates^39,40^. Perhaps the Candida species, such as *Candida albicans*, *tropicalis*, *glabrata*, and *krusei* have the upper hand in causing most fungal infections. The real danger of these pathogens is not only the ease with which they spread but their acquisition of some resistance to antibiotics, not only azole antibiotics^41–43^ but also amphotericin B, especially the species *C. albicans*, *C. glabrata* and *C. krusei*^44,45^. The ability candida species, especially albicans, to infect such diverse hosts is confirmed by a wide range of virulence. A few hallmarks, including the morphological transformation from yeast to hyphal form, the expression of adhesion and invasions in the cell surface, biofilm formation, thigmotropism, response to contact stimulus, secretion of hydrolytic enzymes and phenotype switching in addition to the fast adaptation to the environmental pH-fluctuations^46^. *Aspergillus niger*, is a profiteer pathogen, usually presented in different indoor and outdoor environments^47^. It has special agility features such as spores can be easily aerosolized^48^, carried by air, and potentially can be deposited in bronchioles of the human respiratory system^49^ causing allergic bronchopulmonary aspergillosis which can be lethal in more susceptible patients^50,51^. Like what happens with different types of Candida, the *Aspergillus niger* has also recently shown resistance to some azole antibiotics, such as itraconazole and some triazoles^52–55^. *Mucor circinelloides* is depicted as one of the most frequent species of Mucorales which causes lethal mucormycosis. It was well known that Mucorales infections are difficult to treat easily, as a result of rapid propagation within the target host in addition to their low susceptibility to most of the antifungal agents and eventually drives to miserable scenarios than those made by aspergillosis^56–58^.

**Figure 2:**
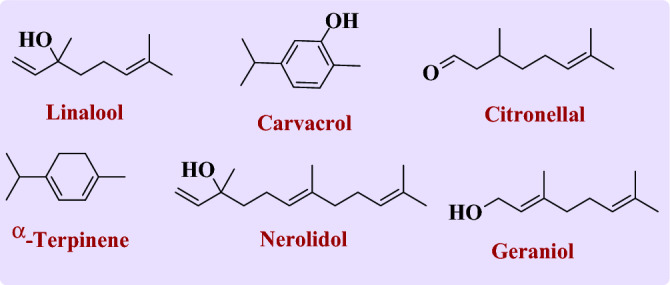
The structure of the selected Monoterpene to be encapsulated into nanostructured lipid carrier (NLC).

In connection to these findings of potential antifungal activity of carvacrol, linalool, citronellal, nerolidol, geraniol, and *α*-terpinene, what will happen if all those terpenes are mixed in definite concentration in one nanoformulation? Are they will affect fungal strains synergistically or anti-synergistically? If this is effective in treatment of fungal infections largely, how to measure the content of the drug loaded or released knowing that, all the chosen terpenes are comparable in their physical properties (liquids, low melting point)? Our passion for knowledge and in continuation of our interest in both synthesis and drug delivery applications^59–70^. We report herein the design of pre-selected, multi-component monoterpenes (Fig. 2) encapsulated into nanostructured lipid carrier (NLC) to test their antifungal activities against five pathogenic fungal strains, trying to determine what if the fluconazole and the pre-selected MTs work synergistically or not and to introduce a novel reliable chromatography approach to estimate the concentration of the drug-loaded and drug released.

## Materials and methods

### Materials

Lauric acid 98%, oleic acid 99%, sodium glycocholate 97%, sodium tourocholate 97%, tween 20 98%, decarbonated water supplied from Sigma Alderich-Germany. Mono Terpenes, geraniol 99%, carvacrol 98%, linalool 97%, nerolidol 97%, *α*-terpinene 90%, citronellal 93% and *n*-hexane (HPLC-grad) were purchased from ACROS ORGANICS-Germany. Fluconazole powder was supplied as a gift from Amoun Pharmaceuticals Company, Obour city, Cairo, Egypt. All the chemical solvents and reagents were used without any purification or distillation. Monoterpenes (MT) selected to be encapsulated according to their miscibility in normal hexane and/or dichloromethane where all the selected MT completely miscible in *n*-hexane.

### Preparation of nanostructured lipid carrier loaded with natural terpenes (NLC-MTs)

Nanostructured lipid carrier’s nanoformulation were synthesized according to hot homogenization method^71^. with little modifications, using a 50 ml beaker (B1, aqueous phase) 2.5 ml of tween 20 was added to a well stirred and filtered mixture of 0.01 g sodium glycocholate and 0.01 g sodium taurocholate dissolved in 10 ml distilled water. Using a pre-adjusted low-temperature water bath, the beaker (B1) was placed at a hotplate stirrer and shaken for a few minutes while the solution temperature was raised to 45 °C and kept warm to that temperature.

Parallel, solid and liquid lipids were mixed in a ratio of (1:2) and exactly, 500 mg (0.5 g) of lauric acid and 1000 mg (1 g) oleic acid were placed in a 50 ml beaker (B2 lipid part). Using a micropipette and 4-digit balance, weigh exactly 25 mg of each single natural terpenes geraniol, carvacrol, citronellal, *α*-terpinene, linalool, and nerolidol. The weighted amounts were injected separately, using a micropipette, in the middle of the oleic acid liquid lipid phase layer (all chosen MTs are completely miscible with oleic acid) to avoid the decomposition or volatilizing of natural terpenes as possible, then the mixture was placed at the water bath with the same conditions (low temperature) until all lauric acid converted from solid to its liquid phase (m.p 43.2 °C) and all solution became homogenous molten.

On hot (45 °C), the solution (B2) was added rapidly to the solution (B1) and kept heating for two minutes with a short lifetime (1 min) application of high shearing homogenization, and clear microemulsion was obtained. Attain the final solution to cool with stirring for two minutes then 10 ml of ice-cold water was added with sonication using a probe sonicator (200w for 7 min) keeping the solution cold till sonication finished. Then the final concentration was completed to 40 ml solution by adding distilled water and kept in a 50 ml falcon tube with a total concentration of 150 mg/40 ml. Using cry-protectant sugar, like trehalose or sucrose, the microdispersion converted from liquid to semi-solid substance by freeze-drying lyophilization for 2 days at -50 °C and kept at a temperature below 10 °C.

For fluconazole encapsulation, the same protocol was applied with the addition of 150 mg of fluconazole dissolved in distilled water, and complete solubility was achieved by heating or sonication. The fluconazole solution was added to the aqueous phase and then the mixture was warmed up using a water bath and the protocol was completed as mentioned earlier. The nanoformulation containing fluconazole and six essential oils was labeled as NlC-Flu-MTs whereas, the sample without fluconazole was labeled as NlC-MTs.

### Particle size and surface charge

One of the most important measurements that control the quality of prepared nanoparticles is the hydrodynamic radius and polydispersity index (PDI) which is inspected using dynamic light scattering (DLS) at an angle of 173° in the room temperature. As DLS and PDI, zeta potential supports us with valuable data about nanoparticle's stability. Zeta potential (z.p) was investigated by measuring the frequent shift of the scattered light due to the particle charge at a scattering angle of 12°. Each of the radius, PDI, and Z.p were quantified with (Zeta Sizer Nano Series (HT), Nano ZS, Malvern Instruments, UK) in the Egyptian Petroleum Research Institute (EPRI). 5–10 mg of each liquid nanoformulation was dispersed and sonicated (sonication bath) in 10 mL of distilled, and then the sample was placed in quartz cell for analysis. For DLS and PDI, three measurements were analyzed and the best one was chosen.

### Surface morphology by transmission *electron* microscope (TEM)

Transmission Electron Microscope described the internal morphology of NLCs nanoparticles to confirm the regularity of the nanoparticles and consequently shows the aggregations as one of the crucial aspects of nanoparticle stability. High-Resolution Transmission electron microscopy was used (HR-TEM, JSM-7100F) in the Egyptian Petroleum Research Institute (EPRI), Cairo; images were recorded with JEOL JEM-2100-115 high-resolution transmission electron microscopes with accelerating voltage 200 kV. Nearly, 1 µL of the NLCs nanoparticles was diluted with double distilled (1:200) and placed on a 200-mesh carbon-coated grid, and left for 2 min. The excess liquid was removed by cellulose filter paper. Two drops of 2% (w/w) phosphotungastic acid (PTA) were added to the carbon-coated grid for a short time of 10 s. to get hold of negative staining and the excess PTA was adsorbed via filter paper as earlier. Then the grid was fixed on the sample holder and the TEM was adjusted for imaging.

### Monoterpenes abundance, drug release and concentration quantization using gas chromatography–mass spectrometry analysis (GC–MS)

#### Standard preparation

Exactly 10 µl of known concentration of each standard carvacrol, linalool, geraniol, α-terpinene, citronellal and nerolidol are mixed to obtain total volume of 60 µl which is completed to one ml by adding 940 µl of n-hexane or dichloromethane (HPLC grade), 10 µl of the mixture was injected to the GC–MS system (Agilent Technologies). After the GC–MS run is completed, the six monoterpenes are identified based on their mass fragmentation and the peak area will be specific for the standard concentration prepared.

#### Sample preparation

As the standard, the sample preparation was done with the same sequence, 10 µl of the sample was completed to 1 ml by adding 940 µl of *n*-hexane or dichloromethane to keep the dilution fixed in all preparations. The sample may be media from the drug released or free unloaded monoterpenes from centrifugation ultrafiltration which is done to estimate the encapsulation efficiency. 10 µl was taken from the sample mixture and injected to the GC–MS system (Agilent Technologies). After the GC–MS run is completed, the six monoterpenes are separated and identified based on their mass fragmentation and in this case the peak area will be specific for the sample concentration prepared.

### Drug (monoterpenes) release

**Figure 3:**
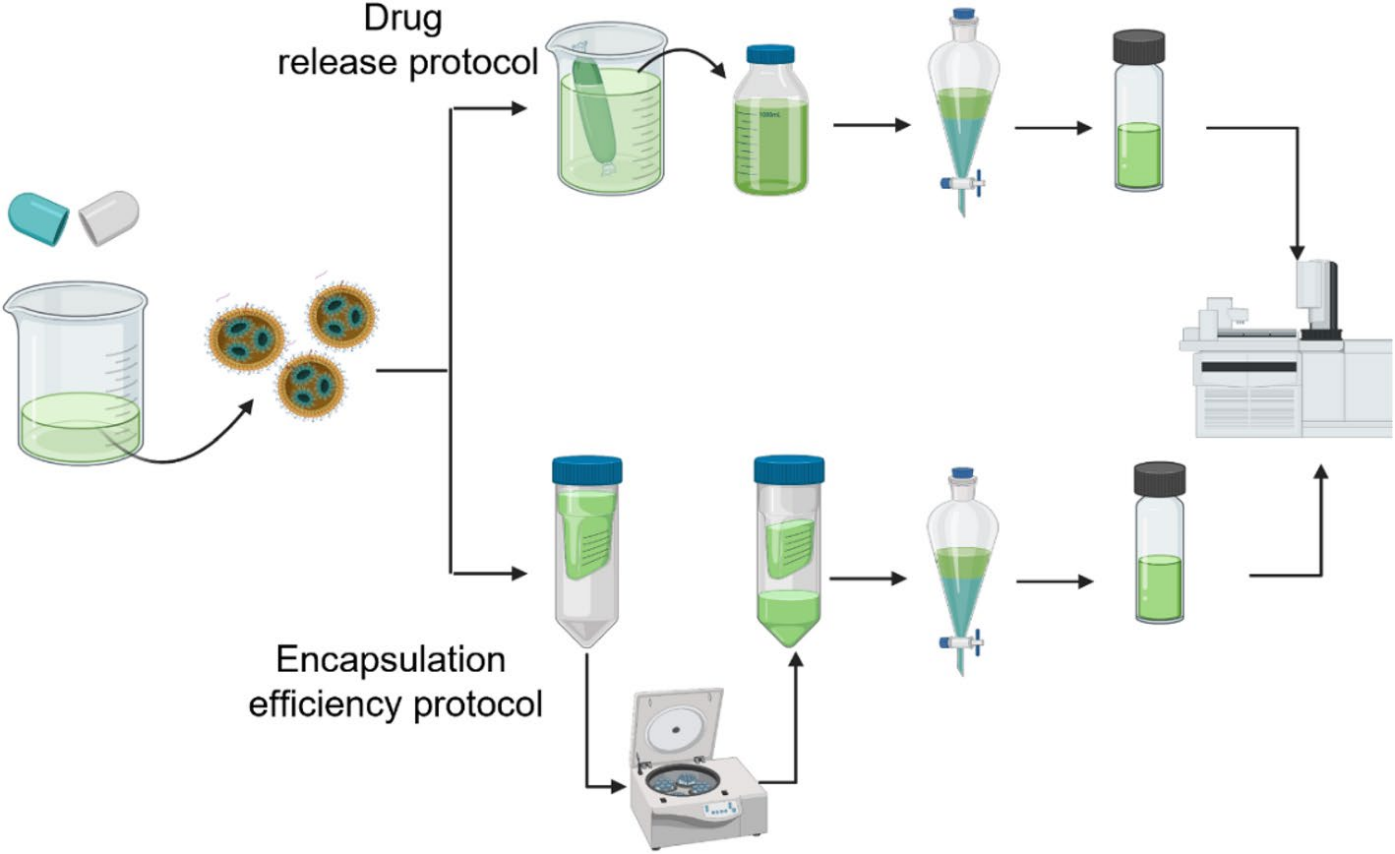
Schematic illustration of the concentration-determination of the six terpenes encapsulated NLC nanoparticles for drug release and encapsulation efficiency.

To determine the released (in-vitro) monoterpene’s abundance and concentration from the synthesized NLC nano-capsule, a dialysis bag protocol was accomplished^72,73^. About 10 cm of dialysis bag was soaked in distilled water for 12 h prior conducting the release study protocol. Nearly 10 ml of NLC-MTs nanoformulation was poured into a dialysis bag (MWCO 12 kDa, Sigma Aldrich) tied at two ends to keep the nanoformulation inside. The dialysis bag was fully immersed in a 250 ml beaker containing 100 ml of distilled water mixed with 2 ml tween 20 which was placed in an incubator at a temperature of 25 °C with occasional stirring from time to time. The solution was taken out and 10 ml of the release media was withdrawn at different time intervals of 0–72 h (batches) whereas, another 10 ml of fresh distilled water was added to compensate for what was lacking. The withdrawn volume of release media containing monoterpenes active ingredients (MTs) released in case of NLC-MTs nanoformulation, and each of MTs and fluconazole in case of NLC-Flu-MTs nanoformulation were directly, mixed with10ml of *n*-hexane as organic phase to extract all MTs and fluconazole (soluble in *n*-hexane)^74^. The extraction step was repeated several times, keeping the dilution factor in account. Solvent extraction was preceded using a proper separatory funnel and this step was repeated three times for the same batch to confirm the complete extraction of natural terpenes then all organic layer of *n*-hexane was collected (for the same batch) and dried using anhydrous magnesium sulfate. Before injection to GC mass, the extracts were concentrated under vacuum to a definite concentration then the sample was injected to GC to evaluate the abundance and concentration regarding the dilution (Fig. 3). The abundance of MTs was identified according to their mass fragmentation and the peak area will be specific for the sample concentration which could be calculated from the Eq. (1).

1 $$sample conc=\frac{sample peak area}{standard peak area}\times standard con.\times dilution factor$$

### Entrapment efficiency

The encapsulation efficiency (E.E) of the prepared NLCs was determined according to centrifugation ultrafiltration method^75^. Using Vivaspin 20 centrifugal concentrator tube. Aliquots of freshly prepared NLC samples (10 ml) were placed on the ultrafiltration tube supported with polyethersulfone membrane (PES) with a 50kDa cut-off (Sigma Aldrich, Germany). After centrifugation (10,000 rpm, 10 min) with Heal Force^@^ (Neofuge 15R refrigerated centrifuge, China), the non-encapsulated (Free) monoterpenes was filtered and mixed with the aqueous media filtration. Like the drug release study, free monoterpenes was collected by adding n-hexane several times by solvent extraction to emphasize complete drug extraction. Hexane-containing free terpenes were collected and dried by passing through anhydrous magnesium sulfate, followed by re-concentration under vacuum to a specific volume keeping the dilution factor into account then the sample was injected into the GC–MS to determine the abundance and concentration of the free non-encapsulated monoterpenes (Fig. 1). The entrapment efficiency is then calculated from Eq. (2).

2 $$E.E \left(\%\right)=\frac{actual encapsulated}{total amount added }=\frac{total amount added of MTs- unloaded MTs}{total amount added of MTs}$$

### GC–MS analysis

GC–MS system (Agilent Technologies) was equipped with a gas chromatograph (7890B) and mass spectrometer detector (5977A) at Central Laboratories Network, National Research Centre, and Cairo, Egypt. Samples were diluted with hexane (1:19, v/v). The GC was equipped with a DB-624 column (30 m × 320 μm internal diameter and 1.8 μm film thickness). Analyses were carried out using helium as the carrier gas at a flow rate of 3.0 ml/min at a split 1:20 of, injection volume of 1 µl and the following temperature program: 40 °C for 1 min; rising at 7 °C/min to 250 °C and held for 5 min. The injector and detector were held at 250 °C. Mass spectra were obtained by electron ionization (EI) at 70 eV; using a spectral range of 30–440 m/z and solvent delay of 6 min. Identification of different constituents was determined by comparing the spectrum fragmentation pattern with those stored in Wiley and NIST Mass Spectral Library data. Semi-quantitative data were obtained from GC–MS area percentages, the retention time and peak area for each single natural terpene were used to calculate the concentration. Peak identification was performed by comparing the relative retention time of each compound with those of standard materials.

## Biology evaluation

### In-vitro antimicrobial screening (inhibition zone)

The Clinical and laboratory standards institute (CLISI) recommended the most popular technique for estimating antimicrobial susceptibility testing as the well diffusion method. One of the main potential usefulness of this disk diffusion method is that results could be obtained after a relatively short time as an inhibition zone diameter in mm within 16–48 h of the incubation that is shorter incubation time than the method described by the M38. The tested fungus strains of *candida albicans (ATCC-10221)*, *Candida Tropicalis (ATCC-131803), Candida krusei (ATCC-14243), Candida glabrata (ATCC-15126), Geotrichom candidum (ATCC-34614), Aspergillus niger (KU681408)* and *Mucor citcinelloid (ATCC-8542)* were ± and the inculcation preparation was done using direct colony suspension as follows; the inoculum was prepared by making a direct broth or saline suspension of the isolated colonies within 24 h in an agar plate using the non-selective medium, such as blood agar. The suspension adjusted to achieve turbidity equivalent to 0.5 McFarland standard which leads to approximately 1 × 10^8^ CFU/ml by using either visual or spectrophotometric method. The antimicrobial assessment using the well diffusion method is usually used to assess synthetic compounds as well as plant extracts, whereas the agar plate surface was inoculated by wiping the target microbial inoculum over the whole agar surface. Holes with a diameter of 6–8 mm were punched aseptically with a sterile cork borer or a tip, and a volume (20–100 µl) of specific concentration (10 mg/ml dissolved in 0.9% saline or DMSO) of the antimicrobial agent under investigation after dissolution in a specific solvent (have no antimicrobial properties) was introduced into the well. The plates were incubated under suitable conditions depending on the tested microorganism at a temperature of 35 ± 2 °C in an ambient air incubator within 15 min of adding the inoculum. To maintain the same incubation temperature for all cultures, do not stack micro-dilution trays more than four high. To prevent drying, seal each tray in a plastic bag, with plastic tape, or with a tight-fitting plastic cover before incubating. After incubation time has passed (24 h is recommended in the case of *Aspergillus niger, Mucor citcinelloid, Candida tropicalis, Candida albicans, Candida krusei, Candida glabrata and Geotrichum candidum* depending on the nature of the microorganism. From its name, the antimicrobial agent diffuses through the agar medium and causes growth inhibition^76,77^. The resulting inhibition zone diameters surrounding the wells should be measured to the nearest whole millimeter at the point at which there is a prominent reduction in growth. If growth is insufficient at the recommended times, the plates should be re-incubated and read later.

### Determination of minimum inhibitory concentration

Minimum inhibitory concentration (MIC) shows the minimum or lowest concentration of an antimicrobial agent that prevents the visible growth of a microorganism in broth dilution susceptibility test. The sample (10 mg) was dissolved in 10 ml distilled water to prepare the concentration of 1000 µg/ml as a stock solution. Based on the broth micro dilution method^78^, two-fold dilutions of antimicrobial agent in a broth were prepared (1000, 500, 250, 125, 62.5, 31.25, 15.6, 7.8, 3.9, 1.95 and 0.97 μg/ml) were prepared. The inoculum was prepared by taking a few colonies from the agar plate with a sterile swap to prepare McFarland standard which diluted with the media. Dispense the antimicrobial/broth solutions into the 96 plastic microdilution trays and incubation processed. Turbidity indicates growth of the microorganism, and the MIC is the lowest concentration where no growth is visually observed.

## Results and discussion:

### Particle size and distribution

**Figure 4:**
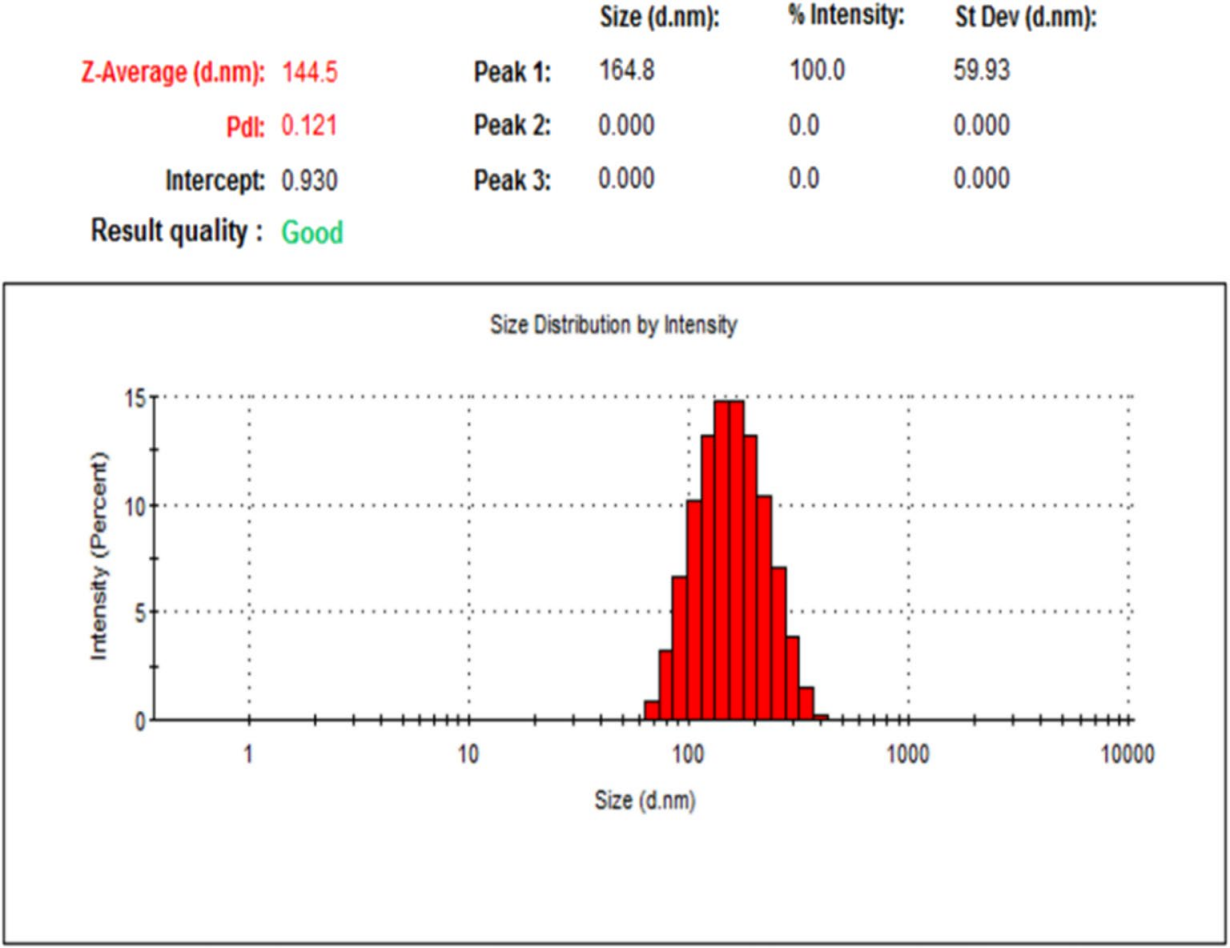
Size distribution and polydispearsity index of (NLC-FLu-MTs).

To identify at which range of size the sample was prepared, DLS was done and the particle size exposed 144.5 and 138.6 nm for NLC-Flu-MTs and NLC-MTs, respectively as shown in Figs. 4 and 6. The particle size of the nanostructured lipid carrier encapsulated with monoterpenes active ingredients only revealed a smaller size which may confirm that increasing the number of loading components affected the particle size and made the droplet size larger. In addition to DLS, the particle homogeneity size distribution or polydispersity index, (PDI) is a crucial factor promoting stability. Smaller values of pdi, from 0.0 to 0.3, point out to more homogenous particles and very narrow size distribution, and the particles near to be monodispersed but when the value of pdi > 0.3, the particles will act as poly dispersed^79^. The values of pdi presented by NLC-Flu-MTs and NLC-MTs were 0.121 and 0.066, respectively which means the addition of fluconazole as an extra component makes severe changes in the physiochemical properties of the particle distribution, via making size-varied particles and that breaks the homogeneity to some extent and makes the pdi value in case of NLC-Flu-MTs became larger.

Flu-MTs but both nanoformulation even with or without fluconazole still homogenous solution that as presented in Figs. 4 and 6.

**Figure 5:**
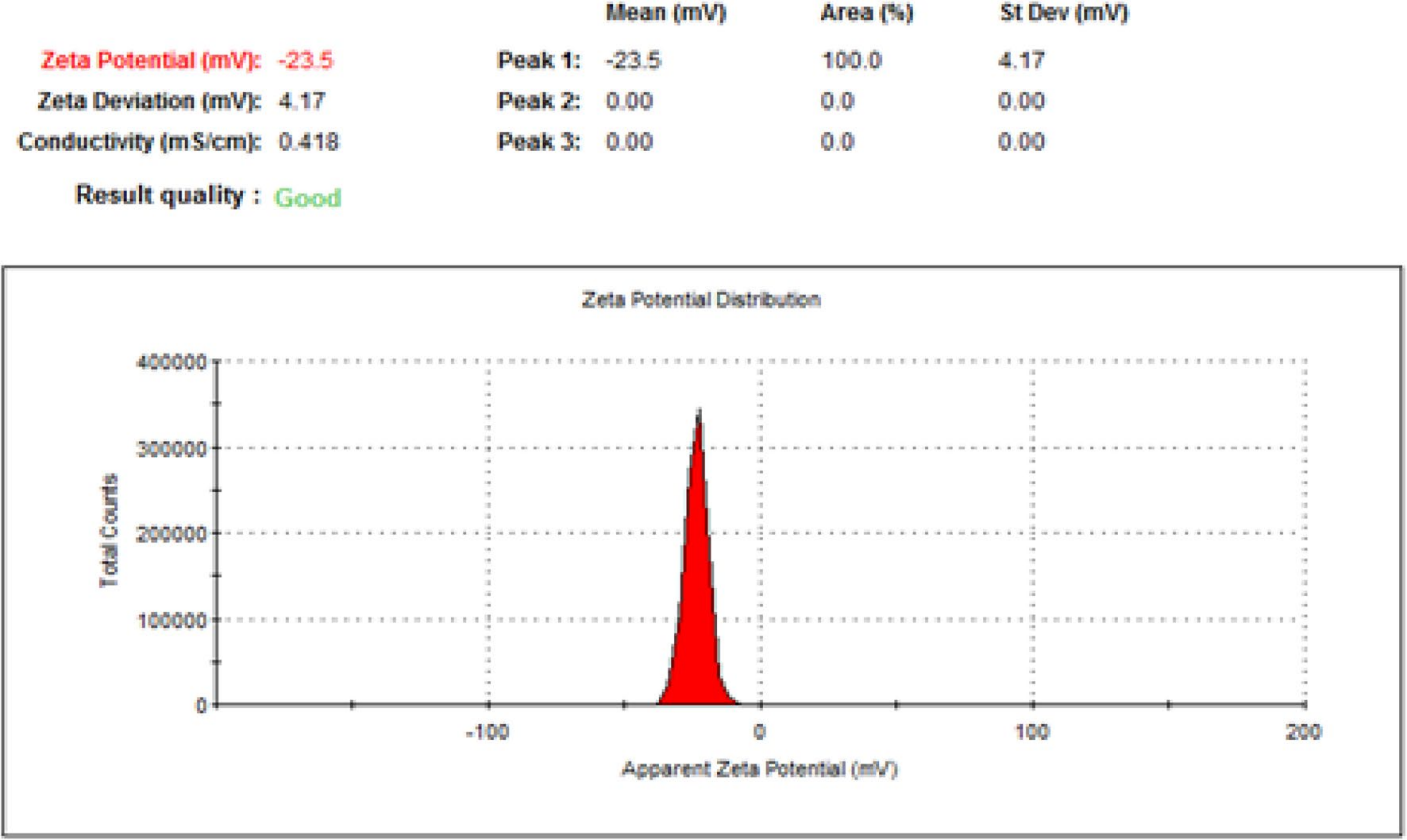
Zeta potential of (NLC-Flu-MTs).

**Figure 6:**
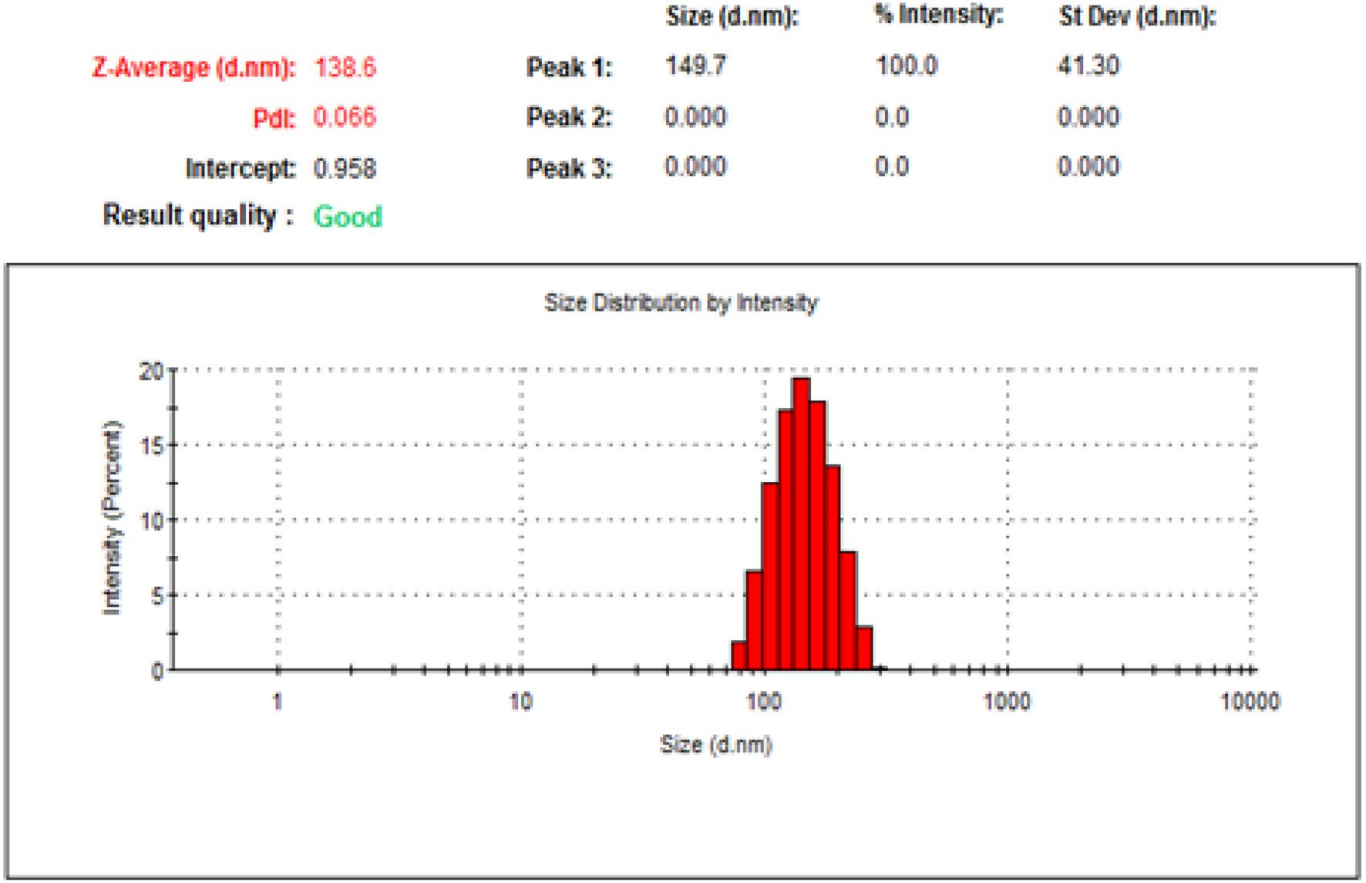
Size distribution and polydispearsity index of (NLC-AI).

**Figure 7:**
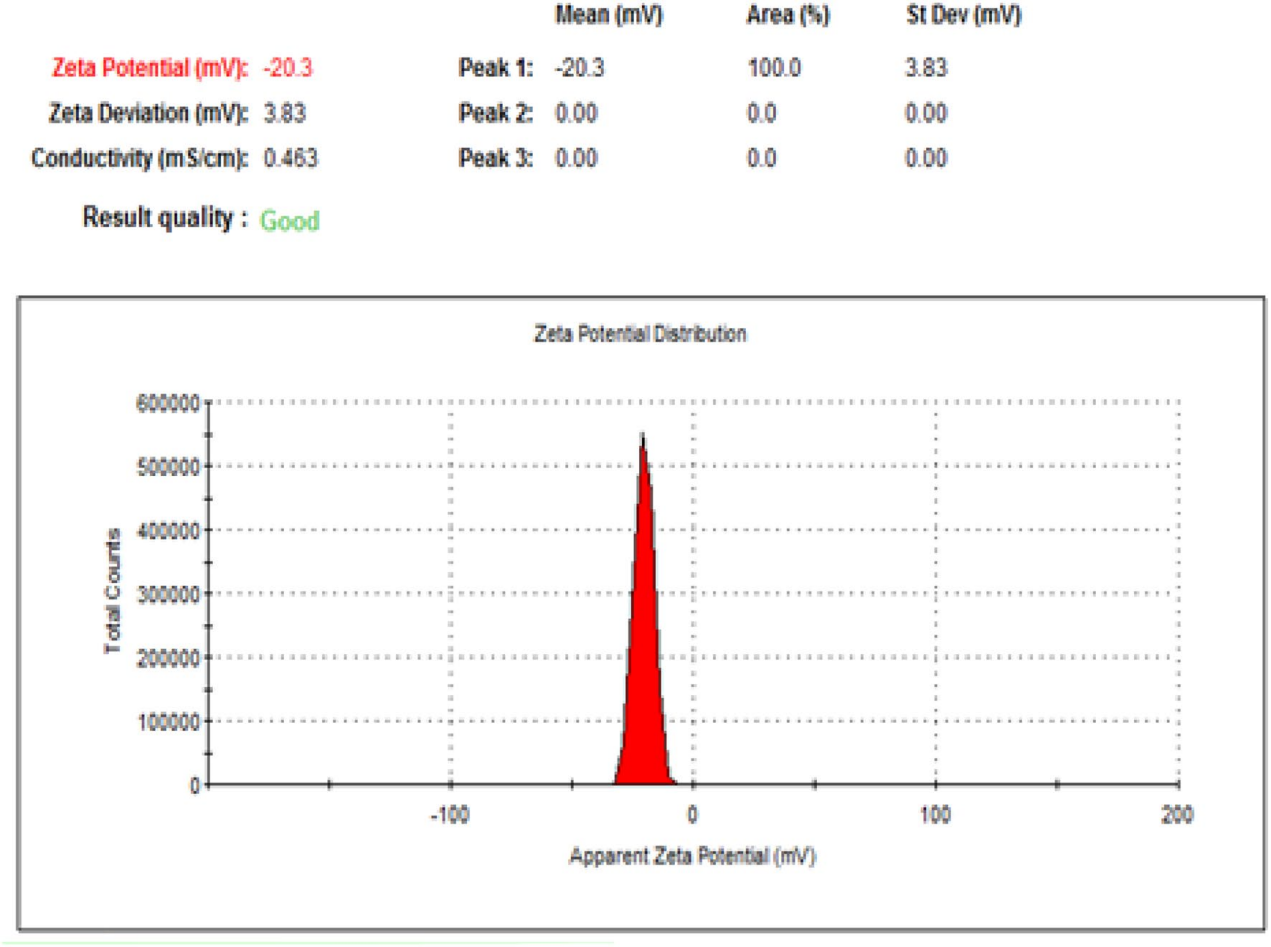
Zeta potential of (NLC-FLU-MTs).

Zeta potential is one of the extremely important physical stability parameters, as increasing the charge, even if negative or positive, indicates a more stable colloidal system. The nanoformulation of NLC-Flu-MTs showed z.p value of − 23.5 mV whereas, NLC-MTs presented − 20.3 mV. The values of z.p presented in Figs. 5 and 7 confirmed that the two nanoformulations showed good physical stability as we move away from the zero-charge point (positive or negative) which encourages better stability. The reason for high stability is due to high repulsive forces between the same charges that make the chances of aggregation diminished (Figs. 6, 7).

### NLC surface morphology by transmission *electron* microscope (TEM)

**Figure 8:**
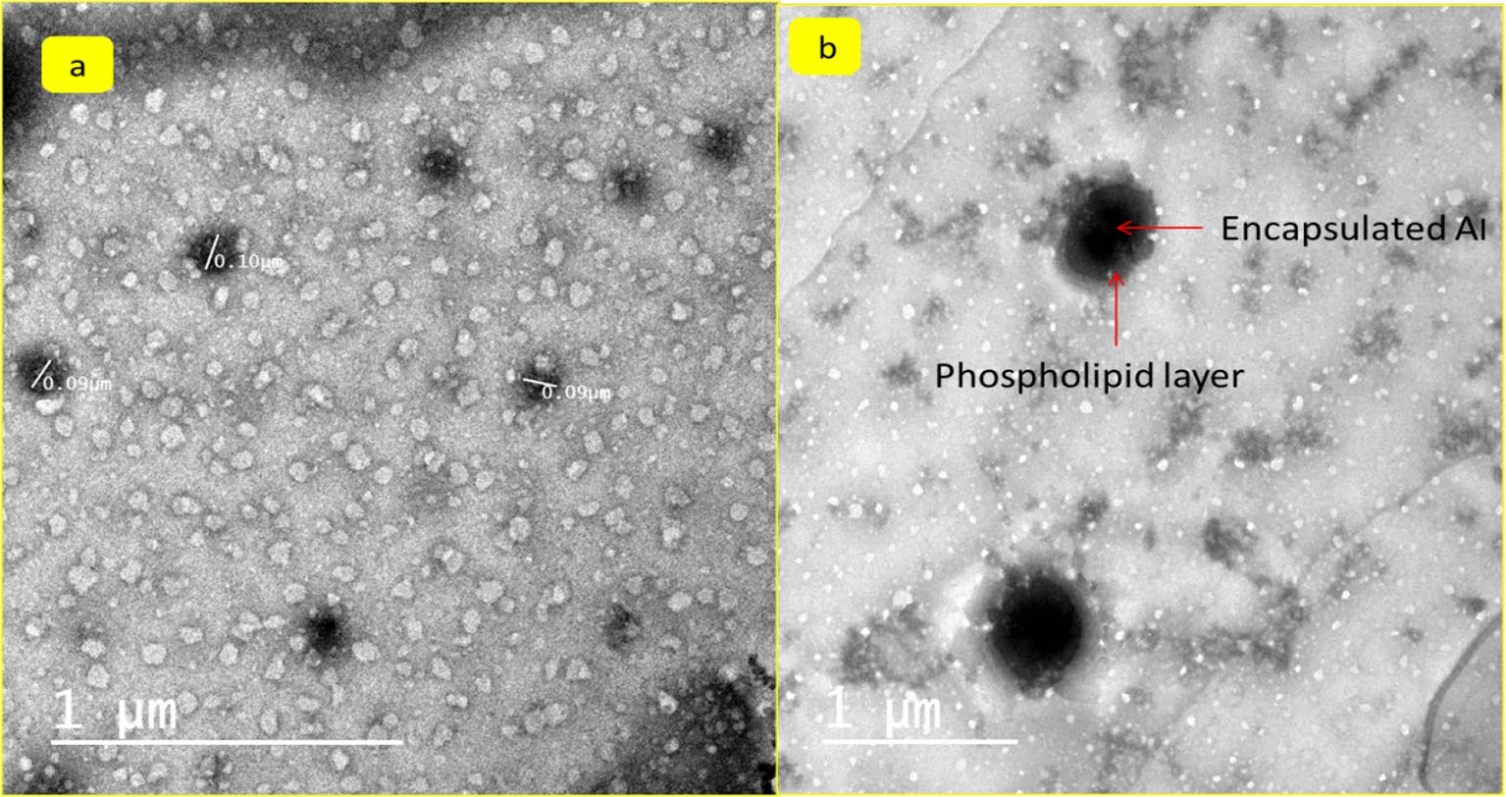
Internal structure and morphology by transmission electron microscope.

For a high-resolution view of the internal morphology, a transmission electron microscope was used to show the shape of the nanoparticles prepared. The field shown in Fig. 8a approved that the prepared NLC-MTs have regular and spherical shapes in a range of size 90 up to 100 nm. The particle became larger, and the morphology changed a little bit to be a spherical, semispherical, and oval shape. The encapsulation process is shown in Fig. 8b the results from DLS and z.p confirmed the same consequences of size and homogeneity, also TEM showed no aggregation at most of the field imaged and that is inconsistent with the values of PDI, especially in case NLC-MTs. The internal dark area shown in Fig. 8b represented the encapsulated active ingredients in the vicinity of NLC meanwhile, the gray outer layer represented the phospholipid and surfactant layer.

### Encapsulation efficiency, in-vitro monoterpenes release and their concentration quantization by GC–MS

The use of a nanocarrier is a breakthrough tool that helps us to reach sites it was previously difficult to access and the quantity in addition to concentration of the drug released (one component) could be estimated easily via a different protocol like dialysis bag, Franz-type diffusion cells^80^ and consequently the drug loading capacity calculated smoothly. Based on different literatures on natural active ingredients, herein we designed a tailored nanoformulation consisting of multiple effective antifungal drugs of six mono terpenes co-delivered with fluconazole. The synthesized nanoparticles tested for their antifungal activity, and promising results were obtained especially, NLC-Flu-MTs*.* The potential effect of nanoformulation is due to the synergistic effect of all components together.

There was a mandatory need to know the quantity of the actual drug loaded, the main problem here was all mono terpenes have comparable physical properties and light absorbability. The choices are limited, and each method has its drawbacks, the concentration determination could be carried out using spectrophotometry such technique, that is very reliable and was used widely to determine the concentration^61,81^ via making a standard curve, and the concentration is calculated directly by comparison. The encapsulation of six monoterpenes at the same nanoformulation makes the concentration determination using UV spectrophotometry may be not accurate due to the comparable ƴ_max_ for each one of the monoterpenes used in this study that makes the measurements interferential.

**Figure 9:**
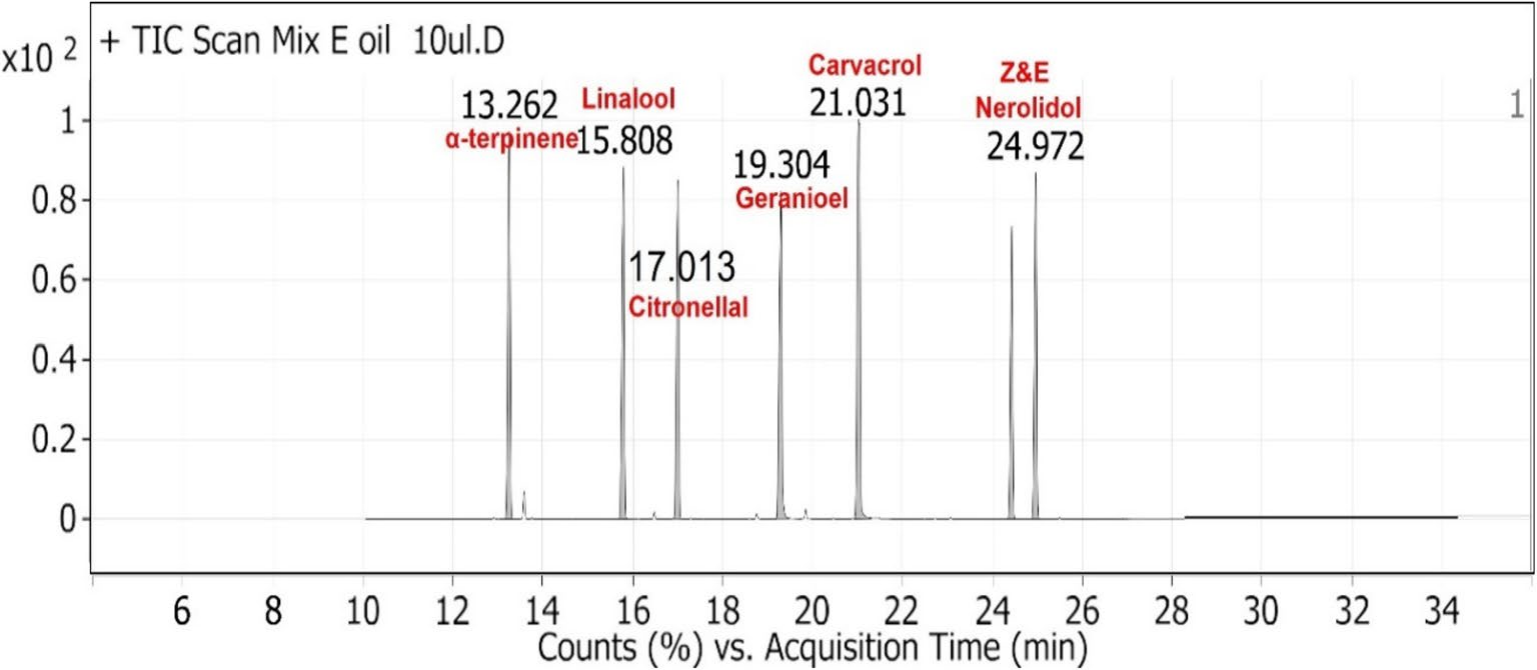
Chromatogram of the standards mixture.

**Figure 10:**
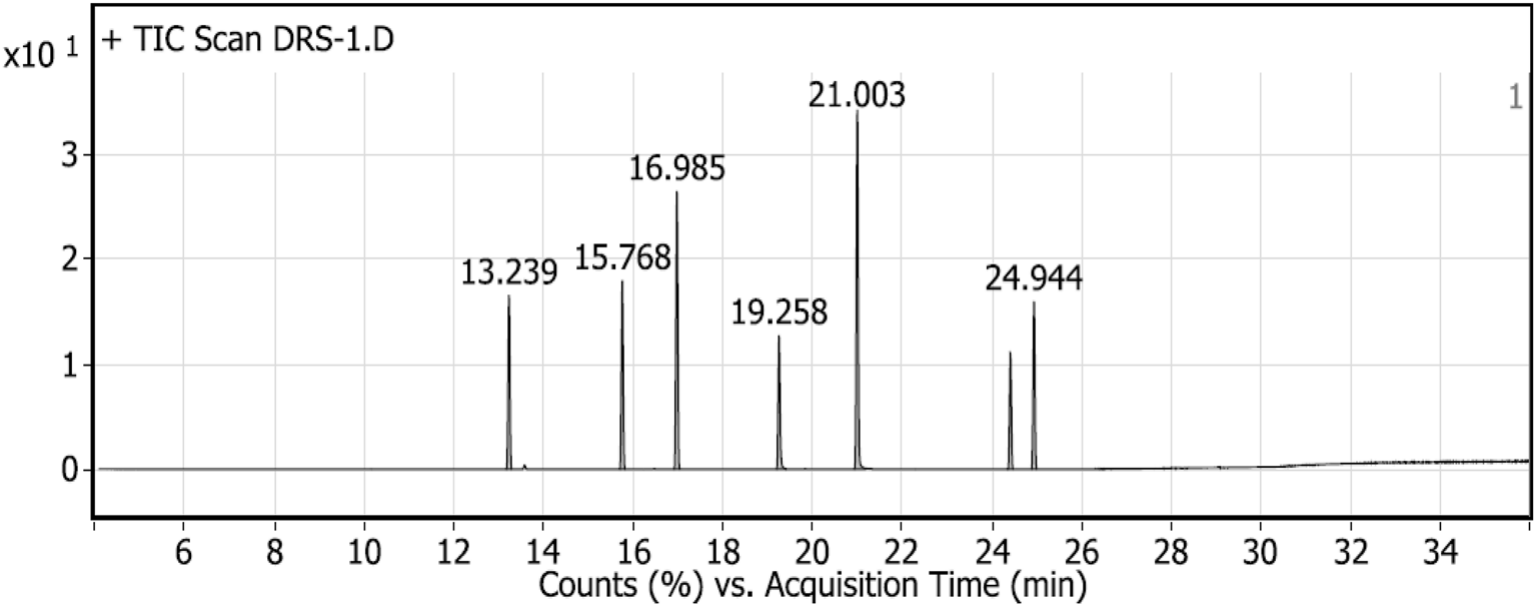
Chromatogram of the drug release sample after 2 h.

**Figure 11:**
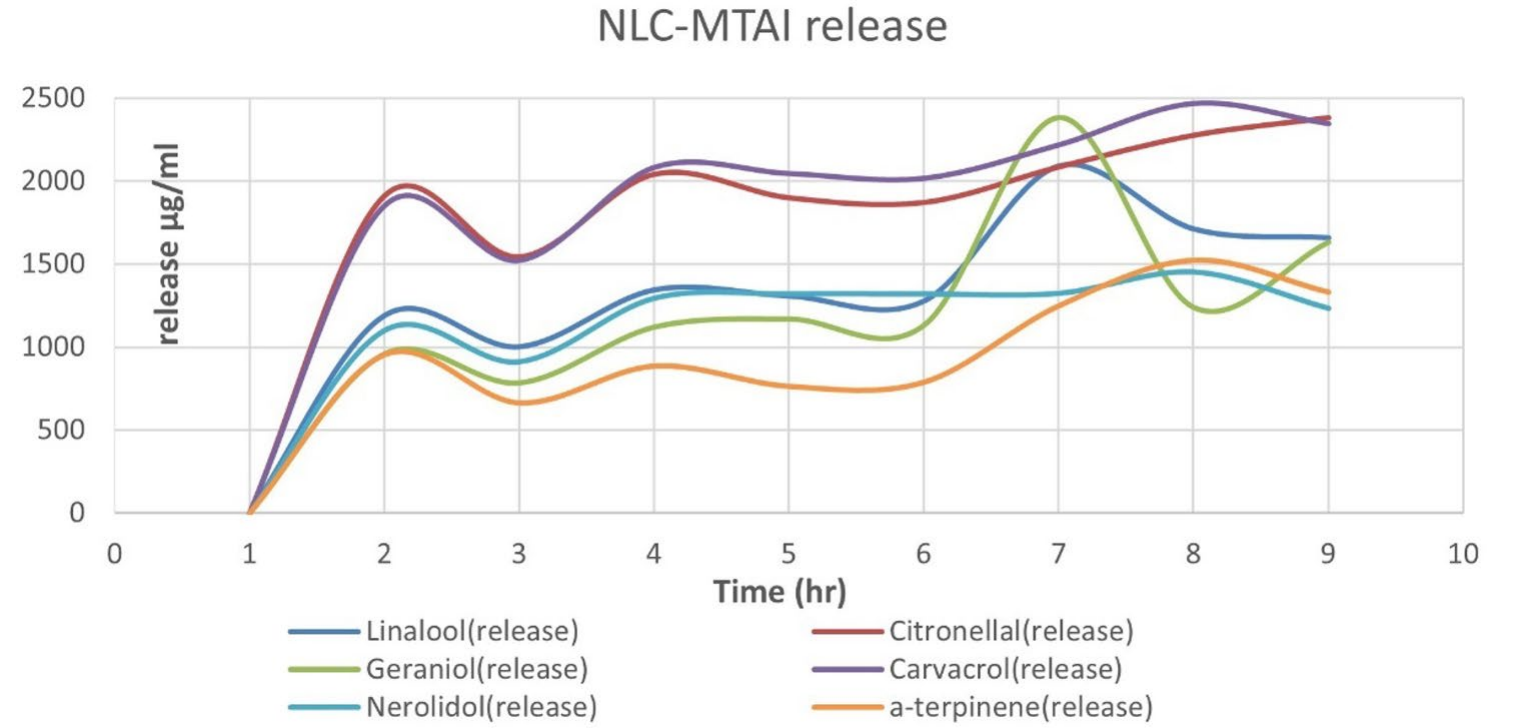
Drug release of the NLC-MTs active ingredients.

Gas chromatography–mass spectrometry (GC–MS) is one of the most reliable tools to estimate the concentration of volatile components like an essential oil in a mixture^82^ and the concentration determination step requires a known concentration of the standard to be injected and consequently, the separation parameters of the area percentage, retention time and peak area for each component obtained which is very specific to the standard itself. Knowing the sample and standard separation parameters, the concentration determination is no longer difficult and has become accessible. After the standards (mixture) injection, the chromatogram (Fig. 9) showed seven peaks instead of six peaks, the reason for the extra peak comes from the existence of two isomers of nerolidol which appeared at two different retention times of 24.438 and 24.972 (min) for the isomers *Z*-nerolidol and *E*-nerolidol, respectively in addition to six single peaks at retention times of 21.03, 19.30, 17.01, 15.80 and 13.26 min (Table 1). Based on the fragmentation pattern and mass spectra, the identification of every single monoterpene was accomplished by comparing the spectrum fragmentation pattern with those stored in Wiley and NIST Mass Spectral Library data. The basic, six retention times were best fit for monoterpenes: carvacrol, geraniol citronellal, linalool, and *α*-terpinene, respectively, the corresponding standards peak area related to concentration and detected peak area of monoterpenes with related concentration after two hours drug release listed in Table 3. Generally, the synthesized NLCs exhibited very good encapsulation efficiency (Table 5) ranging from 84 up to 98%. The encapsulation efficiency was determined by estimating the unloaded or free non-encapsulated MTs using ultrafiltration certification, such tool permits the free MTs to be separated from NLC loaded, the free MTs identified and concentration determined in the same manner discussed before, and consequently the loaded MTs by contrary way using Eqs. (1) and (2). For the MTs drug release study, the samples taken from the release media at time intervals 0 (before release, at the beginning) up to 72 h were processed and identified as the same as standards. Similarly, the same seven peaks were obtained, confirming that all encapsulated monoterpenes released successfully including the presence of the same isomers *Z*-nerolidol and *E*-nerolidol. The integration peak list (Table 2) at the first two hours (Fig. 10), for instance, is used to calculate the exact concentration of each monoterpene released via Eq. (1), thus the total monoterpene e.g., carvacrol, released over 72 h equals the summation of microgram released from time zero to 72 h via Eq. (3). Despite the non-aqueous nature of the loaded oils, they will be liberated effectively in a short period in the release media and according to Table 4 and Fig. 11 it was found that the carvacrol release curve (purple) and citronellal release curve (red curve) have generous drug release where they exonerated more than 67% (16.5 mg) of its original (25 mg) concentration through the first 72 h, regarding to their entrapment efficiency 97.8% and 94.9%, respectively which could be calculated by Eq. (4). It’s noteworthy that the order of drug release capabilities ascendingly from the lowest to the highest one as *α*-terpinene, geraniol, nerolidol, linalool, citronellal and carvacrol, respectively (Tables 1, 2, 3, 4, 5).

3 $${\text{Total release}}\left( {\mu {\text{g}}/{\text{ml}}} \right) = {\text{summation of all released micrograms for the same monoterpenes through 72}}\;{\text{hr}}$$

**Table 1:** Integration peak list of standards mixture.

Peak	Start	RT	End	Height	Area	Area %
1	13.173	13.262	13.339	406,678.88	1,541,316.38	74.17
2	15.707	15.808	15.889	369,513.3	1,497,195.3	72.04
3	16.918	17.013	17.11	355,431.53	1,359,477.83	65.42
4	19.194	19.304	19.488	344,003.85	1,436,341.25	69.11
5	20.936	21.031	21.322	418,592.55	2,078,204.8	100
6	24.349	24.438	24.515	306,904.98	1,077,254.34	51.84
7	24.879	24.972	25.061	363,567.01	1,372,377.83	66.04

**Table 2:** Integration Peak List of drug release sample after 2 h.

Peak	Start	RT	End	Height	Area	Area %
1	13.175	13.239	13.304	69,502.79	195,193.74	47.05
2	15.709	15.768	15.827	75,352.49	213,206.8	51.39
3	16.914	16.985	17.05	111,116.87	324,678.91	78.26
4	19.204	19.258	19.424	53,495.51	158,403.54	38.18
5	20.938	21.003	21.187	143,524.38	414,874.08	100
6	24.35	24.416	24.481	46,241.92	131,032.41	31.58
7	24.885	24.944	25.009	67,077.94	188,644.32	45.47

**Table 3:** Injected standards monoterpenes peak area and related concentration.

Compound	Area	Conc. (µg/ml)
STD (standards)		
α-Terpinene	1,541,316.4	7.533
Linalool	1,497,195.3	8.3517
Citronellal	1,359,477.8	7.998
Geraniol	1,436,341.3	8.692
Carvacrol	2,078,204.8	9.272
Nerolidol	2,449,632.2	8.439
Drug release sample 1 (DRS-1)		
α-Terpinene	195,193.74	95.40
Linalool	213,206.8	118.93
Citronellal	324,678.91	191.01
Geraniol	158,403.54	95.86
Carvacrol	414,874.08	185.10
Nerolidol	319,676.73	110.13

Detected peak area of monoterpenes and related concentration after 2 h drug release.

**Table 4:** Drug released (D.R) of MTs versus time and release percentage.

Compound	Concentration of released MTS (µg/ml)									Total release µg/ml	Total release mg/ml	Entrapment efficiency (%)	Released percent (%) after 72 h
	0 h	2 h	4 h	6 h	8 h	10 h	24 h	48 h	72 h	–	–	–	–
α-Terpinene	0	954 ± 55	663.7 ± 29	885.4 ± 58	763.3 ± 55	788.1	1248.4	1034	1331.1	8157.3	8.1573	93.4	34.935
Linalool	0	1189.3	1001.2	1343.6	1306.8	1276.5	2090.3	1711	1657.8	11,576.1	11.5761	94.6	48.948
Citronellal	0	1910.1	1541.8	2039.4	1897.6	1869.9	2083.9	2274	2380.1	15,996.7	15.9967	94.9	67.426
Geraniol	0	958.6	784.5	1120.4	1169	1131.4	2383.3	1239	1632.8	10,418.9	10.4189	96.2	43.322
Carvacrol	0	1851	1520.9	2081.2	2044.2	2015.6	2216.8	2466	2344.8	16,540.8	16.5408	97.8	67.652
Nerolidol	0	1101.3	912.4	1295.7	1321.5	1322	1325.3	1454	1234.7	9967.2	9.9672	84.5	47.182

**Table 5:** Free unloaded MTs peak area, concentration, and encapsulation efficiency.

Free unloaded MTs				Loaded MTs = total added-free drug (µg/ml)	EE (%) = (Loaded MTS/total drug added) × 100
Compound	Area	Conc. (µg/ml)	(Dilution factor) Conc. × 10 (µg/ml) unloaded MTs		
α-Terpinene	336,845.03	164.63	1646.28	23,353.71009	93.415
Linalool	240,331.13	134.06	1340.6	23,659.37764	94.638
Citronellal	216,087.01	127.13	1271.27	23,728.72961	94.915
Geraniol	153,981.21	93.18	931.8	24,068.18475	96.273
Carvacrol	119,301.11	53.23	532.2	24,467.73297	97.871
Nerolidol	1,121,231.44	386.27	3862.650172	21,137.34983	84.549

4 $$\text{Release percentage }\left(\text{\%}\right)=\frac{\text{Total drug released}}{\text{actual drug loaded}}=\frac{\text{Total drug released}}{\text{Total drug }\left(25\text{mg for each one}\right)\times Entrapment effeciency percentage}$$

### Antifungal activity

#### In-vitro antimicrobial screening (inhibition zone)

Herbal medicine is a very important class of medicinal chemistry and its therapeutical effect extended to include treatment of different diseases. The use of plant extracts as antifungal was proven in many studies in the last ten years and all researchers participating in these studies attributed the reason for the activity of these extracts to the fact that they contain natural compounds like monoterpenes, alkaloids, and others. From the literature, it was found that each of carvacrol, linalool, *α*-terpinene, citronellal, geraniol, and nerolidol has special antifungal properties. We designed herein, a combination of six monoterpenes in one nanostructured lipid carrier (2nd generation of lipid nanoparticles) nanoformulation and studied for its antifungal effect, to find out if they work synergistically or anti-synergistically when combined with fluconazole which almost lost its major activity due to resistance against different fungal strains. The two nanoformulation prepared consist of fluconazole in addition to a six monoterpenes encapsulated within the vicinity of NLC nanoparticles (NLC-FLu-MTs) and without fluconazole (NLC-MTs). The two nanoparticles are in-vitro tested against seven different fungus strains, *C. albicans*, *C. tropicalis, C. krusei, C. glabrata, G. candidum, A. niger,* and *Mucor citcinelloid.* Antimicrobial activity evaluated regarding the inhibition zone diameter and minimum inhibitory concentration presented in Tables 6 and 7. Individual monoterpenes showed modest antimicrobial activity against all the tested microorganisms. Nanoformulation with/without fluconazole depicted a noticeable and sharp change in its activity against most of the studied fungus strains.

**Table 6:** Antifungal screening of the prepared NLCs (zone inhibition diameter).

Fungus strain	Inhibition zone (mm) of each single component and tailored NLCs								
	α-Terpinene	Carvacrol	Nerolidol	Citronellal	Geraniol	FLU	1 (NLC-FLU-MTs)	2 (NLC-MTs)	Cont
*C. tropicalis*	12 ± 0.41	18 ± 0.60	18 ± 0.42	18 ± 0.33	22 ± 0.62	21 ± 0.2	35 ± 0.75	27 ± 0.80	22 ± 0.4
*C. krusei*	NA	19 ± 0.20	NA	18 ± 0.30	NA	14 ± 0.10	27 ± 0.40	22 ± 0.20	13 ± 0.10
*C. glabrata*	NA	NA	NA	14 ± 0.10	NA	11 ± 0.08	30 ± 0.50	14 ± 0.28	15 ± 0.14
*G. candidum*	14 ± 0.11	24 ± 0.00	22 ± 0.18	24 ± 0.22	18 ± 0.15	20 ± 0.18	32 ± 0.45	30 ± 0.64	27 ± 0.33
*C. albicans*	18 ± 0.16	16 ± 0.10	18 ± 0.14	22 ± 0.19	24 ± 0.26	16 ± 0.17	33 ± 0.59	29 ± 0.49	24 ± 0.22
*Asp. niger*	NA	14 ± 0.12	22 ± 0.15	11 ± 0.07	19 ± 0.25	16 ± 0.19	26 ± 0.23	19 ± 0.20	18 ± 0.21
*Mucor circinelloide*	12 ± 0.09	15 ± 0.09	13 ± 0.10	14 ± 0.10	11 ± 0.10	14 ± 0.16	18 ± 0.19	11 ± 0.1	18 ± 0.19

**Table 7:** Minimal inhibitory concentration of NLCs nanoformulations.

Fungus strain	Minimal inhibitory conc. of each single component and tailored NLCs								
	α-Terpinene	Carvacrol	Nerolidol	Citronellal	Geraniol	Flu	1 (NLC-Flu-MTs)	2 (NLC-MTs)	Cont
*C. tropicalis*	62.5 ± 2.33	62,5 ± 2.45	125 ± 8.29	31.2 ± 1.98	62.5 ± 2.93	62.5 ± 2.55	0.97 ± 0.09	15.6 ± 1.13	31.2 ± 2.34
*C. krusei*	NA	125 ± 7.51	NA	125 ± 8.33	NA	62.5 ± 3.14	31.2 ± 1.32	31.2 ± 1.67	125 ± 8.02
*C. glabrata*	NA	NA	NA	125 ± 8.44	NA	NA	31.2 ± 2.15	62.5 ± 1.95	125 ± 6.07
*G. candidum*	62.5 ± 3.11	125 ± 8.77	31.2 ± 1.37	31.2 ± 2.91	125 ± 8.34	125 ± 7.98	3.9 ± 0.69	7.8 ± 0.86	31.2 ± 2.17
*C. albicans*	31.2	31.2	250	62.5	62.5	125	1.9 ± 0.62	3.9 ± 0.99	31.2 ± 1.89
*Asp. niger*	NA	125	125	125	125	NA	15.6 ± 1.02	31.2 ± 1.87	62.5 ± 2.66
*Mucor circinelloide*	31.2	62.5	125	125	NA	NA	31.2 ± 1.13	62.5 ± 2.94	31.2 ± 2.19

**Figure 12:**
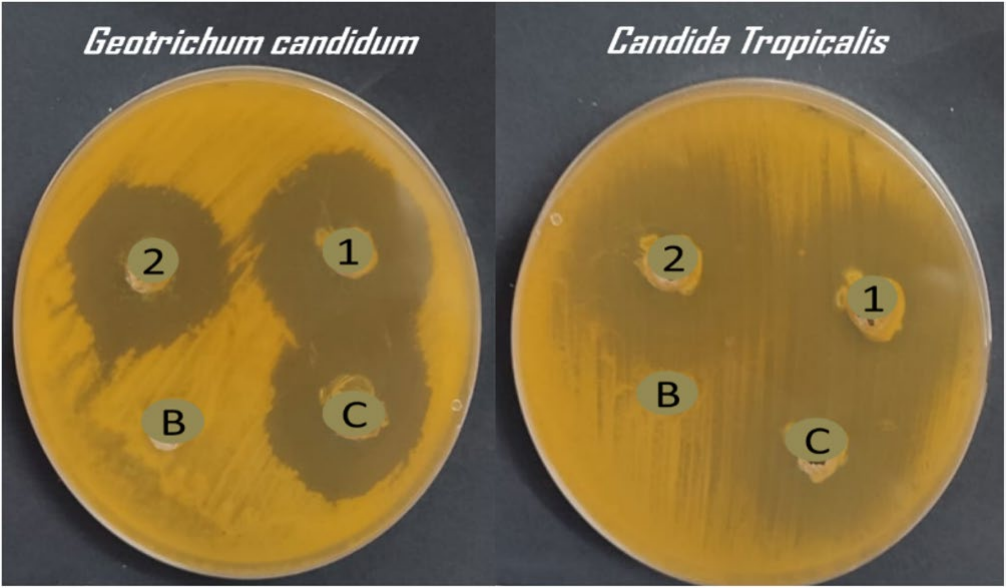
Antifungal evaluation of the encapsulated NLC-Flu-MTs (1), NLC-MTs (2), positive control (c) and untreated blank (B) against *Geotrichum candidum* and *Candida tropicalis*.

**Figure 13:**
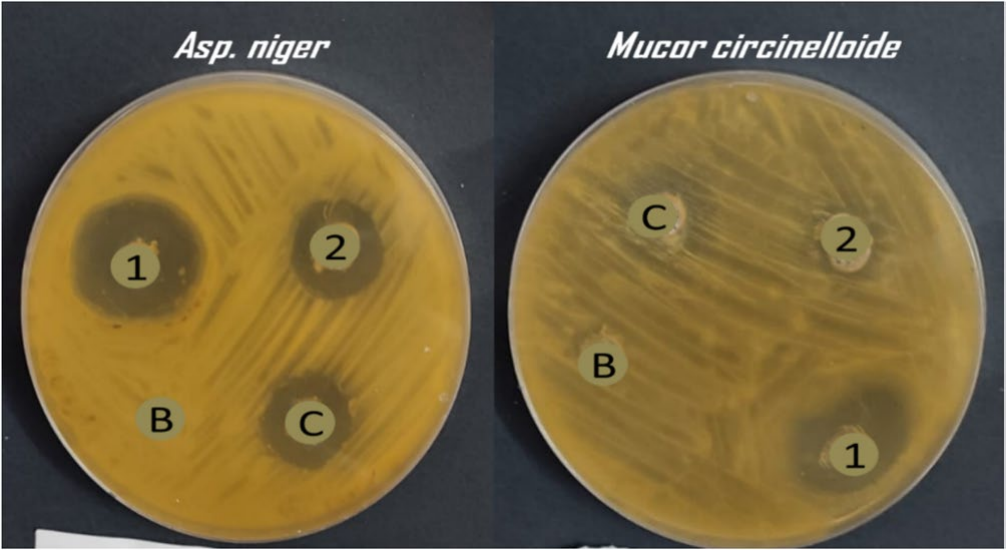
Antifungal evaluation of the encapsulated NLC-Flu-MTs (1), NLC-MTs (2), positive control (C) and untreated blank(B) agains *Asp. niger* and *Mucor citcinelloid*.

**Figure 14:**
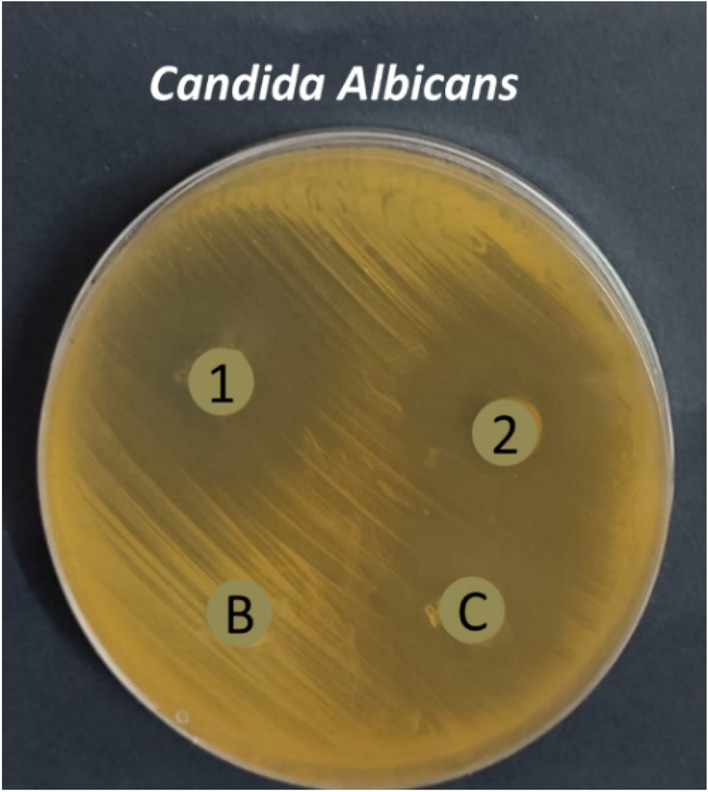
Antifungal evaluation of the encapsulated NLC-Flu-MTs (1), NLC-MTs (2), positive control (c) and untreated blanks against *C. albicans*.

Potential antimicrobial activity exhibited by the nanoformulations of NLC-Flu-MTs and NLC-MTs. The nanoformulation which contains fluconazole combined with monoterpenes active ingredients NLC-FLu-MTs presented broadened inhibition zones (i.z) of 35, 27, 30, 32, 33, 26, and 18 mm, meanwhile nanoformulation without fluconazole NLC-MTs, Which is not much different from the previous one depicted inhibition zones of 27, 22, 14, 30, 29, 19, and 11 mm against the fungus *C. tropicalis*, *C. krusei, C. glabrata, G. candidum*, *C. albicans, A. niger* and *Mucor citcinelloid*, respectively (Table 6). The represented inhibition zones shown in Figs. 12, 13, and 14 controversy settled in favor of fluconazole-based nanoformulation. The most affected fungi by the fluconazole-based NLC were *C. tropicalis* (35 mm) and *G. candidum* (33 mm) strains, especially NLC-FLu-MTs nanoformulation which have the best antifungal sustainability and reduced minimal inhibitory concentration. The minimal inhibitory concentration (MIC) is defined as the lowest concentration of the antimicrobial agent that inhibits the visible microbial growth. According to Table 7, the values of MIC progressed by NLC-Flu-MTs and NLC-MTs were very interesting and promising results exceptionally *C. tropicalis* 0.97 µg/ml and 15.6 µg/ml, respectively). This contrasts with the values given by individual components before the nano system co-delivery. Carvacrol, geraniol, citronellal, nerolidol, and *α*-terpinene, all of these terpenes showed a not-so-great effect on the tested microbes, while the synergistic effect of these compounds together in a single nanoformulation with and /without fluconazole led to an increase in their antimicrobial effect through suppressing its minimal inhibitory concentration 128 folds, for nerolidol (MIC = 125 µg/ml) against *C. tropicalis*, if compared to NLC-Flu-MTs (MIC = 0.97 µg/ml) and 16 folds if compared to NLC-MTs (15.6 µg/ml), against the same fungi, respectively. Although Monoterpenes encapsulated NLC without fluconazole showed very good MIC value, when compared to monoterpenes encapsulated NLC with fluconazole (NLC-Flu-MTs) the MIC diminished by about 16 folds for *C. tropicalis*. The potential antimicrobial activity of NLC-Flu-MTs and NLC-MTs nanoformulation towards different fungus strains promoted that all monoterpenes in addition to fluconazole reacted synergistically causing potential microbial growth inhibition (Supplementary information).

## Conclusion

Carvacrol, geraniol, citronellal, nerolidol, and *α*-terpinene were chosen to be co-delivered with fluconazole to vicinity nanostructure lipid carrier due to their unique antifungal. The accumulative effect of NLC co-delivered MTs in addition to fluconazole was investigated against five pathogenic fungal strains. The six MTs successfully loaded with loading efficiency near to 99% and very good release profile. The synergistic effect of NLC-Flu-MTs nanoparticles showed wide inhibition zones all through most of the tested organisms with very promising minimal inhibitory concentration especially for the *Candida tropicalis* strain of 0.97 µg/ml with very interesting values with the other species. Novel chromatography approach was developed to estimate the abundance and the concentration of monoterpenes loaded and released.

### Supplementary Information

Supplementary Information.

## Data Availability

The datasets used and/or analyzed during the current study available from the corresponding author on reasonable request.

## References

[1] Fernandes CM, Dasilva D, Haranahalli K, McCarthy JB, Mallamo J, Ojima I, Poeta MD (2021). The future of antifungal drug therapy: Novel compounds and targets. Antmicrob. Agents Chemother..

[2] Inanov M, Ciric A, Stojkovic D (2022). Emerging antifungal targets and strategies. Int. J. Mol. Sci..

[3] Palmiori F, Koutsokera A, Bernasconi E, Tunier P, Garnier CV, Ubags N (2022). Recent advances in fungal infections: From lung ecology to therapeutic strategies with a focus on *Aspergillus* spp.. Front. Med..

[4] Tumer SA, Butler G (2014). The candida pathogenic species complex. Cold Spring Harb. Perspect. Med..

[5] Sharma J, Rosiana S, Razzaq I, Shapiro SR (2019). Linking cellular morphogenesis with antifungal treatment and subsceptibility in candida pathogens. Fungi (Basel).

[6] Nanjan MJ, Mohammed M, Kumar PRB, Chandrasekar MJN (2018). Thiazolidinedi-ones as antidiabetic agents: A critical review. Bioorg. Chem..

[7] Metwally NH, Radwan IT, El-Serwy WS, Mohamed MA (2019). Design, synthesis, DNA assessment and molecular docking study of novel 2-(pyridin-2-ylimino)thiazolidin-4-one derivatives as potent antifungal agents. Bioorg. Chem..

[8] Schoustra SE, Debets AJM, Rijs AJMM, Zhang J, Snelders E, Leendertse PC, Melchers WJG, Rietveld AG, Zwaan BJ, Verweij PE (2019). Environmental hotspots for azole resistance selection of *Aspergillus fumigatus*, The Netherlands. Emerg. Infect. Dis..

[9] Jorgensen LN, Heick TM (2021). Azole use in agriculture, horticulture, and wood preservation—Is it indispensable?. Front. Cell. Infect. Microbiol.

[10] Radwan IT, Elwahy AH, Darweesh AF, Sharaky M, Bagato N, Khater HF, Salem ME (2022). Design, synthesis, docking study, and anticancer evaluation of novel bis-thiazole derivatives linked to benzofuran or benzothiazole moieties as PI3k inhibitors and apoptosis inducers. J. Mol. Struct..

[11] Marzi M, Farjam M, Kazemmejad Z, Shiroudi A, Kouhpayeh A, Zarenezhad EA (2022). Recent overview of 1,2,3-triazole-contaning hybrids as novel antifungal agents: Focusing on synthesis, mechanism of action, and structure activity relationship (SAR). J. Chem..

[12] Hitchcock CA (1991). Cytochrome P-450-dependent 14 alpha-sterol demethylase of *Candida albicans* and its interaction with azole antifungals. Biochem. Soc. Trans..

[13] Horne TJ, Hollomon DW (1997). Molecular mechanisms of azole resistance in fungi. FEMS Microbiol. Lett..

[14] Heimark L, Shipkova P, Greene J, Munayyer H, Tomaine TT, Domenico BD, Hare R, Pramanik BN (2002). Mechanism of azole antifungal activity as determined by liquid chromatographic/mass spectrometric monitoring of ergosterol biosynthesis. J. Mass Spectrom..

[15] Yao D, Chen J, Chen W, Li Z (2019). Mechanisms of azole resistance in clinical isolates of *Candida glabrata* from two hospitals in China. Infect. Drug Resist..

[16] Gu W, Guo D, Zhang L, Xu D, Sun S (2016). The synergistic effect of azoles and fluoxetine against resistant *Candida albicans* strains is attributed to attenuating fungal virulence. Antimicrob. Agents Chemother..

[17] Copping VMS, Barelle CJ, Hube B, Gow NAR, Brown AJP, Odds FC (2005). Exposure of *Candida albicans* to antifungal agents affects expression of SAP2 and SAP9 secreted proteinase genes. J. Antimicrob. Chemother..

[18] Martinez RCR, Franceschini SA, Patta MC, Quintana SM, Cardido RC, Ferreira JC, DeMartinis ECP, Reid G (2009). Improved treatment of vulvovaginal candidiasis with fluconazole plus probiotic *Lactobacillus rhamnosus* GR-1 and *Lactobacillus reuteri* RC-14. Lett. Appl. Microbiol..

[19] Sardella D, Gatt R, Valdramidis VP (2017). Assessing the efficacy of zinc oxide nanoparticles against Penicillium expansum by automated turbidimetric analysis. Mycology.

[20] Shi XY, Yang YP, Zhang Y, Li W, Wang JD, Huang WN, Fan YM (2015). Molecular identification and antifungal susceptibility of 186 Candida isolates from vulvovaginal candidiasis in southern China. J. Med. Microbiol..

[21] Yano J, Sobel JD, Nyirjesy P, Sobel R, Willian VL, Yu Q, Noverr MC (2019). Current patient perspectives of vulvovaginal candidiasis: Incidence, symptoms, management and post-treatment outcomes. BMC Womens Health..

[22] Rad MM, Zafarghandi S, Abbasabadi B, Tavallaee M (2011). The epidemiology of Candida species associated with vulvovaginal candidiasis in an Iranian patient population. Eur. J. Obstet. Gynecol. Reprod. Biol..

[23] Hosseini SS, Yadegari MH, Rajabibazl M, Ghaemi EA (2016). Inhibitory effects of carvacrol on the expression of secreted aspartyl proteinases 1–3 in fluconazole-resistant Candida albicans isolates. Iran J. Microbiol..

[24] Paul S, Mohanram K, Kannan I (2018). Antifungal activity of curcumin-silver nano-particles against fluconazole-resistant clinical isolates of Candida species. Ayu.

[25] Hosseini SS, Joshaghani H, Shokohi T, Ahmadi A, Mehrbakhsh Z (2020). Antifungalactivity of ZnO nanoparticles and nystatin and downregulation of SAP1-3 genes expression in fluconazole-resistant candida albicans isolates from vulvovaginal candidiasis. Infect. Drug Resist..

[26] Ghosh M, Mandal S, Roy A, Chakrabarty S, Chakrabarti G, Pradhan SK (2020). Enhanced antifungal activity of fluconazole conjugated with Cu–Ag–ZnO nanocomposite. Mater. Sci. Eng. C Mater. Biol. Appl..

[27] Hamad KM, Mahmoud NN, Al-Dabash S, Al-Samad LA, Abdallah M, Al-Bakri AG (2020). Fluconazole conjugated-gold nanorods as an antifungal nanomedicine with low cytotoxicity against human dermal fibroblasts. RSC Adv..

[28] Lo WH, Deng FS, Chang CJ, Lin CH (2020). Synergistic antifungal activity of Chitosan with fluconazole against candida albicans, candida tropicalis, and fluconazole-resistant strains. Molecules.

[29] Abdellatif AAH, El-Telbany DFA, Zayed G, Al-Sawahli MM (2018). Hydrogel containing PEG-coated fluconazole nanoparticles with enhanced solubility and antifungal activity. J. Pharm. Innov..

[30] Abbaszadeh S, Sharifzadeh A, Shokri H, Khosravi AR, Abbaszadeh A (2014). Antifungal efficacy of thymol, carvacrol, eugenol and menthol as alternative agents to control the growth of food-relevant fungi. J. Mycol. Med..

[31] Zhang J, Ma S, Du S, Chen S, Sun H (2019). Antifungal activity of thymol and carvacrol against postharvest pathogens *Botrytis cinerea*. J. Food Sci. Technol..

[32] Niu C, Wang C, Yang Y, Chen R, Zhang J, Chen H, Zhug Y, Li J, Cheng J, Xu K, Chu M, Ren C, Zhang C, Jia C (2020). Carvacrol induces candida albicans apoptosis associated with Ca^2+^/calcineurin pathway. Front. Cell Infect. Microbiol..

[33] Hsu CC, Lai WL, Chuang KC, Lee MH, Tsai YC (2013). The inhibitory activity of linalool against the filamentous growth and biofilm formation in *Candida albicans*. Med. Mycol..

[34] Lima MIO, Medeiros ACA, Silva KVS, Cardoso GN, Lima EO, Pereira FO (2017). Investigation of the antifungal potential of linalool against clinical isolates of fluconazole resistant *Trichophyton rubrum*. J. Mycol. Med..

[35] Silva KVS, Lima MIO, Cardoso GN, Santos AS, Silva GS, Pereira FO (2017). Inhibitory effects of linalool on fungal pathogenicity of clinical isolates of microsporum canis and microsporum gypseum. Mycoses.

[36] Pereira FO, Mendes JM, Lima IO, Mota KSL, Oliveira WA, Lima EO (2015). Antifungal activity of geraniol and citronellol, two monoterpenes alcohols, against *Trichophyton rubrum* involves inhibition of ergosterol biosynthesis. Pharm Biol..

[37] Li WR, Shi QS, Ouyang YS, Chen YB, Duan SS (2013). Antifungal effects of citronella oil against *Aspergillus niger* ATCC 16404. Appl. Microbiol. Biotechnol..

[38] Vasquez LR, Mesia LR, Ceferino HDC, Mesia WR, Andrés FM, Díaz CE, Coloma AG (2022). Antifungal and herbicidal potential of piper essential oils from the peruvian Amazonia. Plants.

[39] Lockhart SR, Wagner D, Iqbal N, Pappas PG, Andes DR, Kauffman CA, Brumble LM, Hadley S, Walker R, Ito JI, Baddley JW, Chiller T, Park BJ (2011). A comparison of in vitro susceptibility of Candida species from cases of invasive candidiasisin solid organ and stem cell transplant recipients: Transplant-associated infections surveillance network (TRANSNET), 2001 to 2006. J. Clin. Microbiol..

[40] Bagtzoglou AD, Dwivedi P, Ioannidou E, Shaqman M, Hull D, Burleson J (2009). Oral Candida infection and colonization in solid organ transplant recipients. Oral Microbiol. Immunol..

[41] Brilhante RSN, Paiva MAN, Sampaio CMS, Teixeira CEC, Branco DSCMC, Leite JJG, Moreira CA, Silva LP, Cordeiro RA, Monteiro AJ, Sidrim JJC, Rocha MFG (2011). Yeasts from *Macrobrachium amazonicum*: A focus on antifungal susceptibility and virulence factors of Candida spp. FEMS Microbiol. Ecol..

[42] Brito EHS, Fontenelle ROS, Brilhante RSN, Cordeiro RA, Monteiro AJ, Sidrim JJC, Rocha MFG (2009). The anatomical distribution and antimicrobial susceptibility of yeast species isolated from healthy dogs. Vet. J..

[43] Sidrim JJC, Branco DSCMC, Brilhante RSN, Soares GDP, Cordeiro RA, Monteiro AJ (2010). Rocha Candida species isolated from the gastrointestinal tract of cockatiels (Nymphicus hollandicus): In vitro antifungal susceptibility profi le and phospholipase activity. Vet. Microbiol..

[44] Ingroff AE (2009). Novel antifungal agents, targets or therapeutic strategies for the treatment of invasive fungal diseases: A review of the literature (2005–2009). Rev. Iberoam. Micol..

[45] Vyas SP, Gupta S (2006). Optimizing efficacy of amphotericin B through modification. Intern. J. Nanomed..

[46] Nicholls S, MacCallum DM, Kaffarnik FA, Selway L, Peck SC, Brown AJ (2011). Activation of the heat shock transcription factor Hsf1 is essential for the full virulence of the fungal pathogen *Candida albicans*. Fungal Genet. Biol..

[47] Rintala H, Pitkäranta M, Täubel M (2012). Microbial communities associated with house dust. Adv. Appl. Microbiol..

[48] Li X, Zhang T, Wang S (2019). Aerosolization of Aspergillus niger spores from colonies on different positions of a circular tube. E3S Web Conf..

[49] White JK, Nielsen JL, Madsen AM (2020). Potential respiratory deposition and species composition of airborne culturable, viable, and non-viable fungi during occupancy in a pig farm. Atmosphere (Basel).

[50] Barac A, Ong DSY, Jovancevic L, Peric A, Surda P, Spiric VT, Rubino S (2018). Fungi-induced upper and lower respiratory tract allergic diseases: One entity. Front. Microbiol..

[51] Person AK, Chudgar SM, Norton BL, Tong BC, Stout JE (2010). Aspergillus niger: An unusual cause of invasive pulmonary aspergillosis. J. Med. Microbiol..

[52] Jenks J, Hoenigl M (2018). Treatment of aspergillosis. J. Fungi.

[53] Reischies F, Hoenigl M (2014). The role of surgical debridement in different clinical manifestations of invasive aspergillosis. Mycoses.

[54] Snelders E, Veld RAGHI, Rijs AJMM, Kema GHJ, Melchers WJG, Verweij PE (2009). Possible environmental origin of resistance of *Aspergillus fumigatus* to medical triazoles. Appl. Environ. Microbiol..

[55] Azevedo MM, Ramos IF, Cruz LC, Vaz CP, Rodrigues AG (2015). Genesis of azole antifungal resistance from agriculture to clinical settings. J. Agric. Food Chem..

[56] Narbona MT, Guinea J, Alarcon JM, Munoz P, Gadea I, Bouza E (2007). Impact of zygomycosis on microbiology workload: A survey study in Spain. J. Clin. Microbiol..

[57] Kontoyiannis DP, Lewis RE (2011). How I treat mucormycosis. Blood..

[58] Katragkou A, Walsh TJ, Roilides E (2014). Why is mucormycosis more difficult to cure than more common mycoses?. Clin. Microbiol. Infect..

[59] Farghali MA, Selim AM, Khater HF, Bagato N, Alharbi W, Alharbi KH, Radwan TI (2022). Optimized adsorption and effective disposal of Congo red dye from wastewater: Hydrothermal fabrication of MgAl-LDH nanohydrotalcite-like materials. Arab. J. Chem..

[60] Radwan IT, Baz MM, Khater H, Alkhaibari AM, Selim AM (2022). Mg-LDH nanoclays intercalated fennel and green tea active ingredient: Field and laboratory evaluation of insecticidal activities against culex pipiens and their non-target organisms. Molecules.

[61] Metwally NH, Deeb EA (2018). Synthesis, anticancer assessment on human breast, liver and colon carcinoma cell lines and molecular modeling study using novel pyrazolo[4,3-*c*]pyridine derivatives. Bioorg. Chem.

[62] Metwally NH, Badawy MA, Okpy DS (2018). Green synthesis of some new thiopyrano[2,3-*d*][1,3]thiazoles using lemon juice and their antibacterial activity. Synth. Comm.

[63] Metwally NH, Mohamed MS, Ragb EA (2019). Design, synthesis, anticancer evaluation, molecular docking and cell cycle analysis of 3-methyl-4,7-dihydropyrazolo [1,5-*a*]pyrimidine derivatives as potent histone lysine demethylases (KDM) inhibitors and apoptosis inducers. Bioorg. Chem..

[64] Metwally NH, Saad GR, Abdwahab EA (2019). Grafting of multiwalled carbon nanotubes with pyrazole derivatives: Characterization, antimicrobial activity and molecular docking study. Inter. J. Nanomed..

[65] Metwally NH, Abdallah SO, Mohsen MAA (2020). Design, green one-pot synthesis and molecular docking study of novel N, N-bis (cyanoacetyl) hydrazines and bis-coumarins as effective inhibitors of DNA gyrase and topoisomerase IV. Bioorg. Chem..

[66] Metwally NH, Ragab EA, Mohammed MS (2020). Synthesis of some novel N5-sulfonylated and N1-alkyated pyrazole derivatives and their antimicrobial activity in conjunction with molecular docking study. J. Heterocyclic Chem..

[67] Metwally NH, Mohamed MS, Deeb EA (2021). Synthesis, anticancer evaluation, CDK2 inhibition, and apoptotic activity assessment with molecular docking modeling of new class of pyrazolo[1,5-*a*]pyrimidines. Res. Chem. Intermed..

[68] Metwally NH, Koraa TH, Sand SMH (2022). Green one-pot synthesis and in vitro antimicrobial screening of pyrano[2,3-*d*]pyrazoles, 4*H*-chromenes and pyrazolo[1,5-*a*]pyrimidines using biocatalyzed pepsin. Synth. Comm.

[69] Metwally NH, Badawy MA, Okpy DS (2022). Synthesis, biological evaluation of novel thiopyrano[2,3-*d*]thiazole hybrids as potential nonsulfonamide human carbonic anhydrase IX and XII inhibitors: Design, synthesis and biochemical studies. J. Mol. Struct..

[70] Metwally NH, El-Dosoky EA (2023). Novel thiopyrano[2,3-*d*]thiazole-pyrazole hybrids as potential nonsulfonamide human carbonic anhydrase IX and XII inhibitors: Design, synthesis, and biochemical studies. ACS Omega.

[71] Radwan IT, Baz MM, Khater H, Selim AM (2022). Nanostructured lipid carriers (NLC) for biologically active green tea and fennel natural oils delivery: Larvicidal and adulticidal activities against culex pippins. Molecules.

[72] Artigas AM, Pérez LY, Belloso MO (2018). Curcumin-loaded nanoemulsions stability as affected by the nature and concentration of surfactant. Food Chem..

[73] Kamel KM, Khalil IA, Rateb ME, Elgendy H, Elhawary S (2017). Chitosan-coated cinnamon/oregano-loaded solid lipid nanoparticles to augment 5-fluorouracil cytotoxicity for colorectal cancer: extract standardization, nanoparticle optimization, and cytotoxicity evaluation. J. Agric. Food Chem..

[74] Blokhina S, Ol'khovich M, Sharapova A, Perlovich G (2020). Experimental investigation of fluconazole: Equilibrium solubility and sublimation. J. Chem. Thermody.

[75] Cabeza RL, Kah M, Grillo R, Bílkováa Z, Hofman J (2021). Is centrifugal ultrafiltration a robust method for determining encapsulation efficiency of pesticide nanoformulations?. Nanoscale.

[76] Magaldia S, Essayaga SM, Caprilesa CH, Pereza C, Colella MT, Olaizolaa C, Ontiverosb Y (2004). Well diffusion for antifungal susceptibility testing. Inter. J. Infect. Dise.

[77] Ingroff EA, Canton E, Fothergill A, Ghannoum M, Johnson E, Joned RN, Zeichner LO, Schell W, Gibbs DL, Wang A, Turnidge J (2011). Quality control guidelines for amphotericin B, itraconazole, posaconazole and voriconazole disk diffusion susceptibility tests with non-supplemented Mueller-Hinton agar (M51-A document) for nondermatophyte filamentous fungi. J. Clin. Microbiol..

[78] Wiegand I, Hilpart K, Hancocpa REW (2008). Agar and broth dilution methods to determine the nminimal inhibitory concentration (MIC) of antimicrobial substances. Nat. Protocols.

[79] Narawi MN, Chiu HI, Yong YK, Zain NNM, Ramachandran MR, Tham CL, Samsurrijal SF, Lim V (2020). Biocompatible nutmeg oil-loaded nanoemulsion as phyto-repellent. Front. Pharmacol..

[80] Yu M, Yuan W, Li D, Schwendeman A, Schwendeman SP (2019). Predicting drug release kinetics from nanocarriers inside dialysis bags. J. Control Release.

[81] Apalli VK, Kaul V, Gorantla S, Waghule T, Dubey SK, Pandey MM, Singhvi G (2020). UV Spectrophotometric method for characterization of curcumin loaded nanostructured lipid nanocarriers in simulated conditions: Method development, in-vitro and ex-vivo applications in topical delivery. Spectrochimica Acta Part A Mol. Biomol. Spectrosc..

[82] Pedersen DS, Capone DL, Skouroumounis GK, Pollnitz AP, Sefton MA (2003). Quantitative analysis of geraniol, nerol, linalool, and *α*-terpineol in wine. Anal. Bioanal. Chem..

