# Supplementary material for: Synergistic and potential antifungal properties of tailored, one pot multicomponent monoterpenes co-delivered with fluconazole encapsulated nanostructure lipid carrier
Source: Sci Rep. 2024 Jun 22;14:14382. doi: 10.1038/s41598-024-63149-x (PMC11193721; doi:10.1038/s41598-024-63149-x)
Supplement: Supplementary file 1 — Supplementary Information. [file 41598_2024_63149_MOESM1_ESM.pdf]

Supplementary data drug release study (DRS)

Mix oils (standards, known concentration)

Qualitative Analysis Report

**Data Filename**	Mix E oil 10ul.D	**Sample Name**	Mix E oil 10ul
**Sample Type**		**Position**	1
**Instrument Name**	Head Space	**User Name**	
**Acq Method**	Essnestial oil DB-624 (He).M	**Acquired Time**	5/29/2023 1:03:19 PM
**IRM Calibration Status**	Not Applicable	**DA Method**	SignalToNoiseCheckout.m
**Comment**			

**Expected Barcode**		**Sample Amount**	
**Dual Inj Vol**	1	**TuneName**	ATUNE.U
**TunePath**	D:\MassHunter\GCMS\1\5977 \	**TuneDateStamp**	2023-05-09T16:01:14+02:00
**MSFirmwareVersion**	6.00.34	**OperatorName**	
**RunCompletedFlag**	True	**Acquisition SW Version**	MassHunter GC/MS Acquisition 10.0.368 14-Feb-2019 Copyright © 1989-2018 Agilent Technologies, Inc

User Chromatograms

Fragmentor Voltage | **Collision Energy** 0 | **Ionization Mode** EI

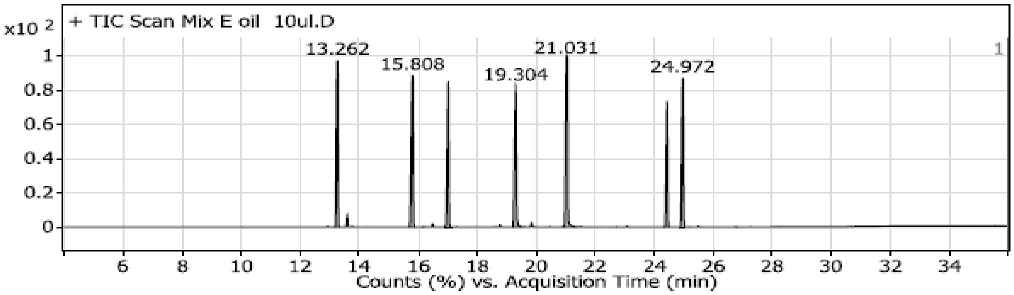

Integration Peak List

Peak	Start	RT	End	Height	Area	Area %
1	13.173	13.262	13.339	406678.88	1541316.38	74.17
2	15.707	15.808	15.889	369513.3	1497195.3	72.04
3	16.918	17.013	17.11	355431.53	1359477.83	65.42
4	19.194	19.304	19.488	344003.85	1436341.25	69.11
5	20.936	21.031	21.322	418592.55	2078204.8	100
6	24.349	24.438	24.515	306904.98	1077254.34	51.84
7	24.879	24.972	25.061	363567.01	1372377.83	66.04

User Spectra

Spectrum Source	Collision Energy	Ionization Mode
Peak (1) in "+ TIC Scan"	0	EI

Qualitative Analysis Report

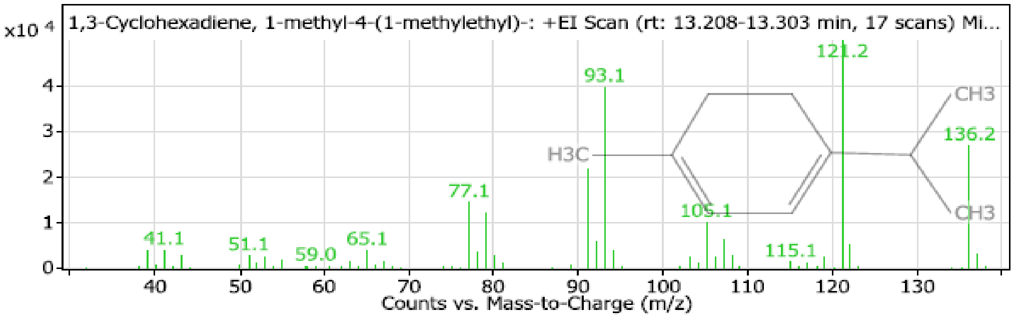

Library Spectrum

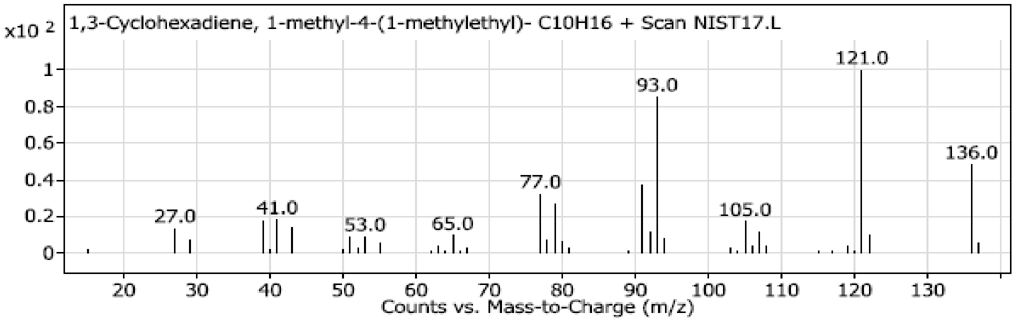

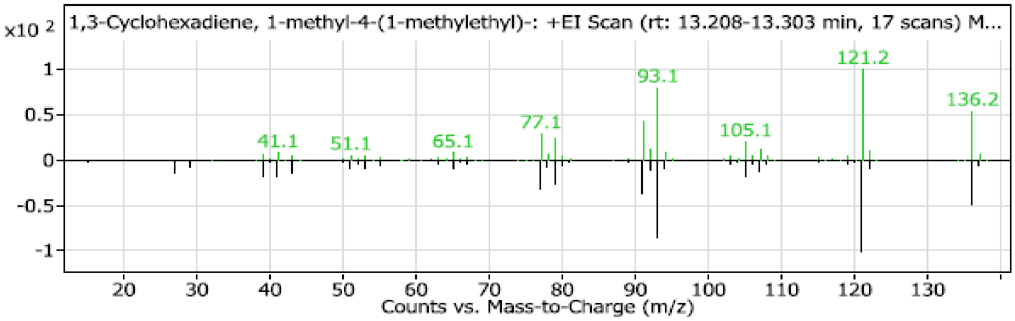

Spectrum Structure

1,3-Cyclohexadiene, 1-methyl-4-(1-methylethyl)-

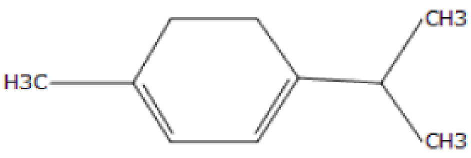

Qualitative Analysis Report

Spectrum Source	Collision Energy	Ionization Mode
Peak (2) in "+ TIC Scan"	0	EI

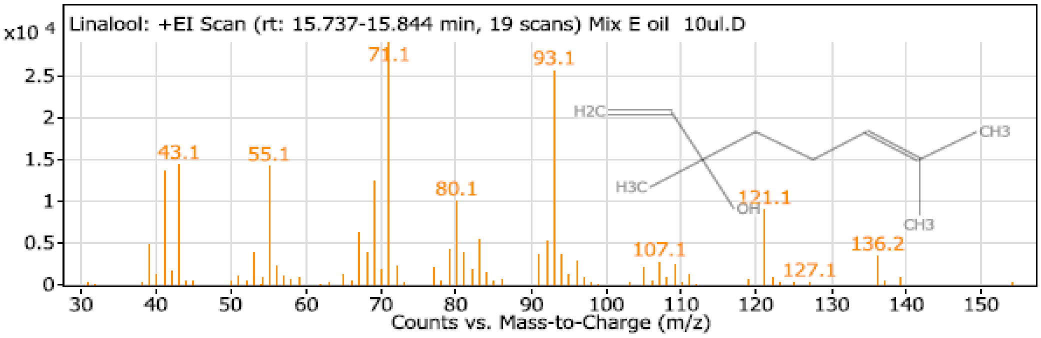

Library Spectrum

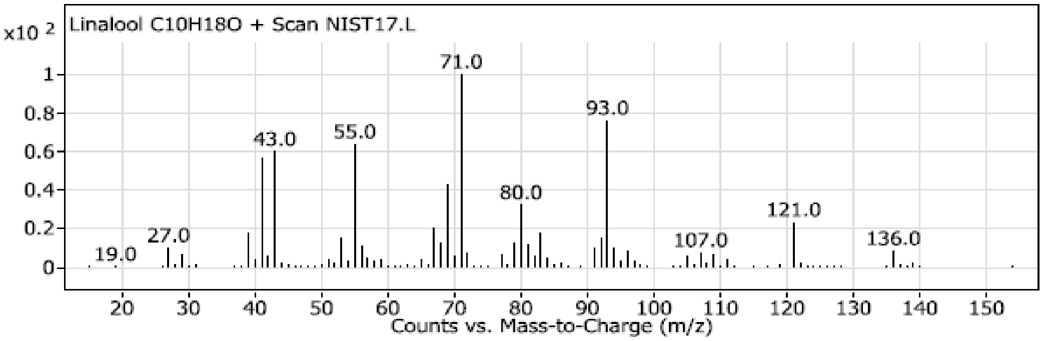

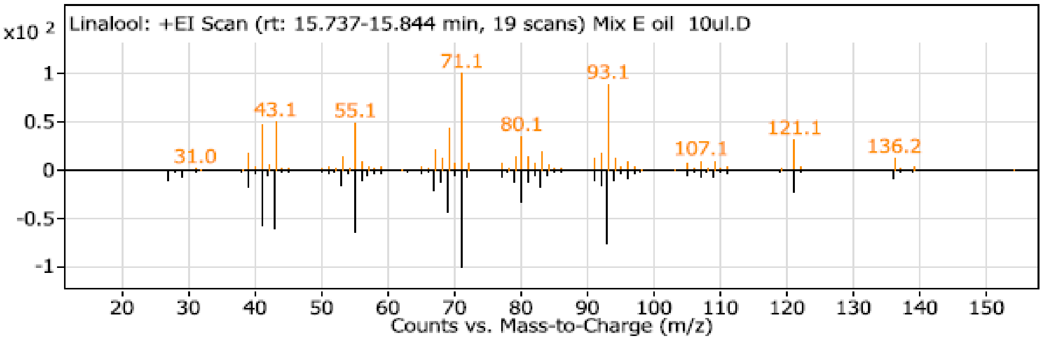

Spectrum Structure

Linalool

Spectrum Source	Collision Energy	Ionization Mode
Peak (2) in "+ TIC Scan"	0	EI

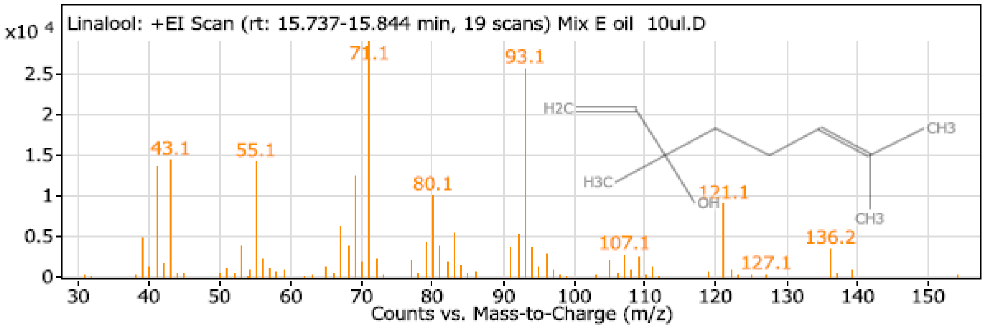

Library Spectrum

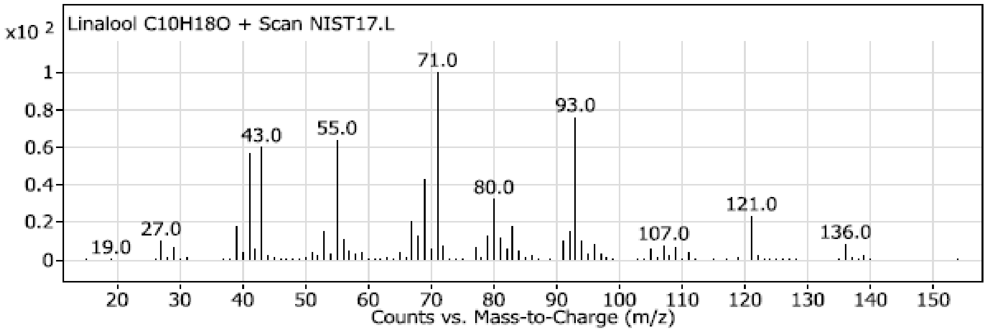

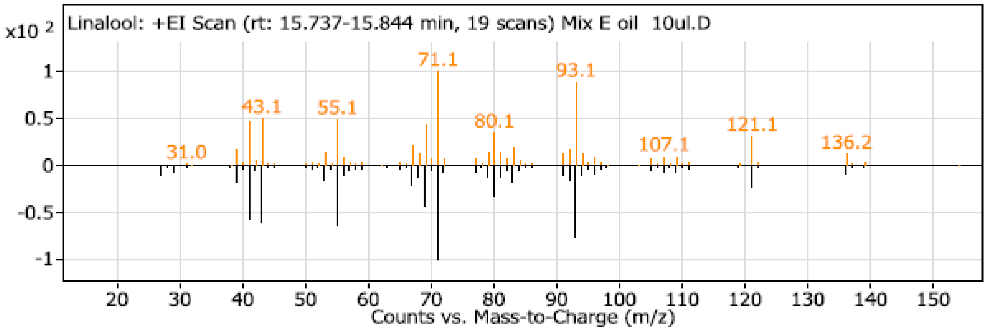

Spectrum Structure

Linalool

Qualitative Analysis Report

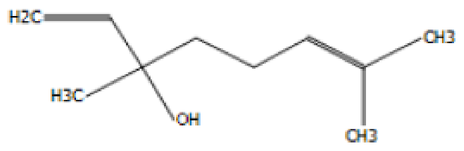

Spectrum Source	Collision Energy	Ionization Mode
Peak (3) in "+ TIC Scan"	0	EI

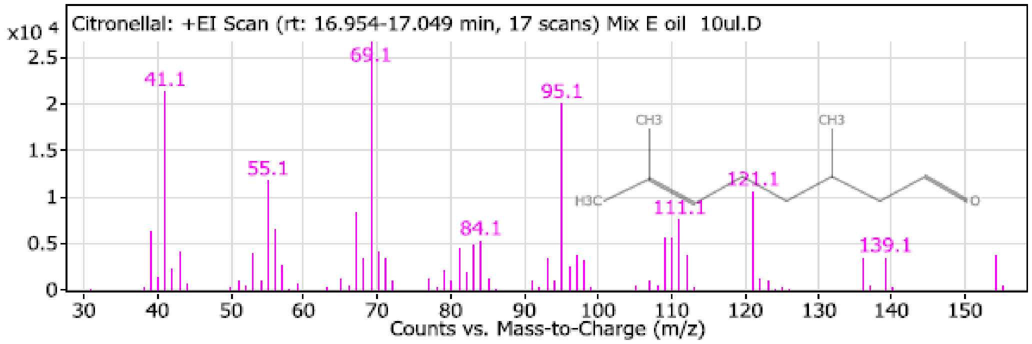

Library Spectrum

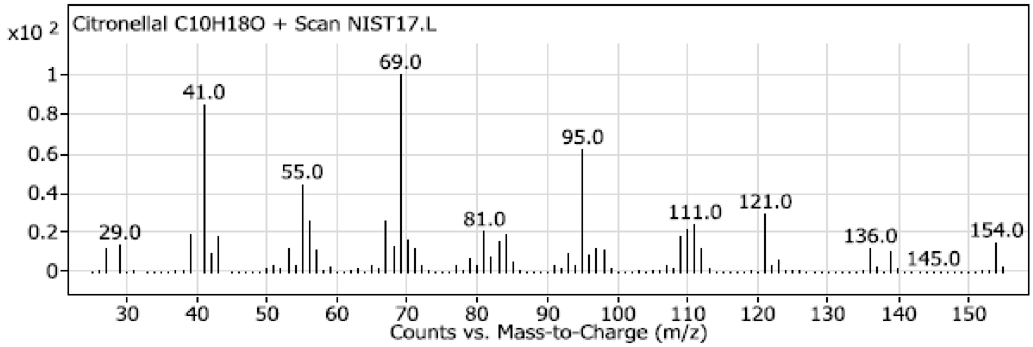

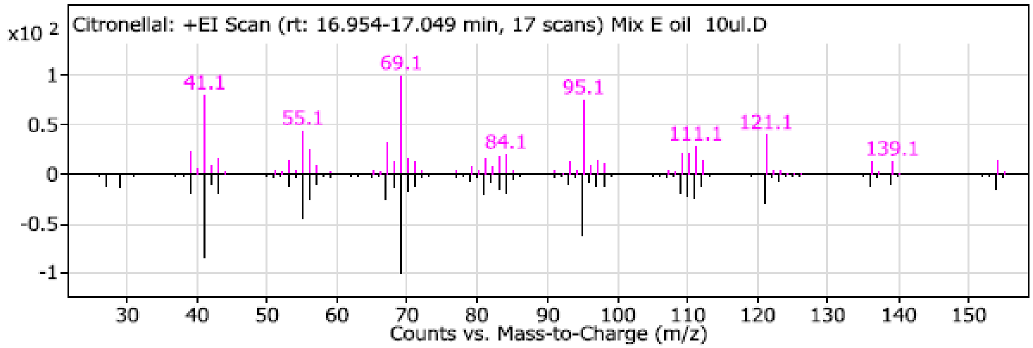

Spectrum Structure

Citronellal

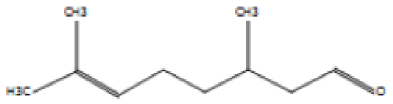

**Spectrum Source**	**Collision Energy**	**Ionization Mode**
Peak (4) in "+ TIC Scan"	0	EI

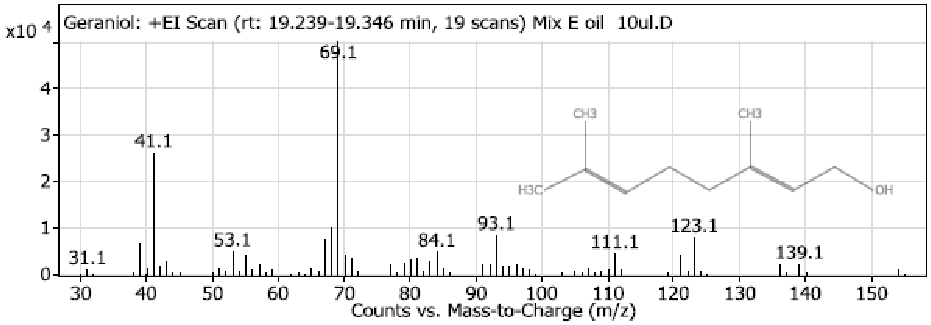

Library Spectrum

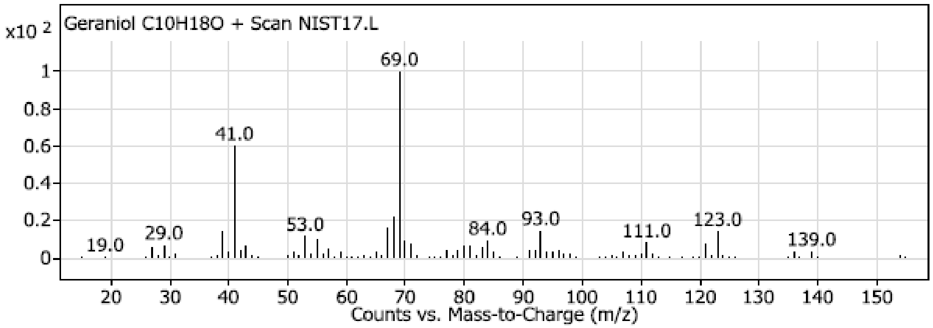

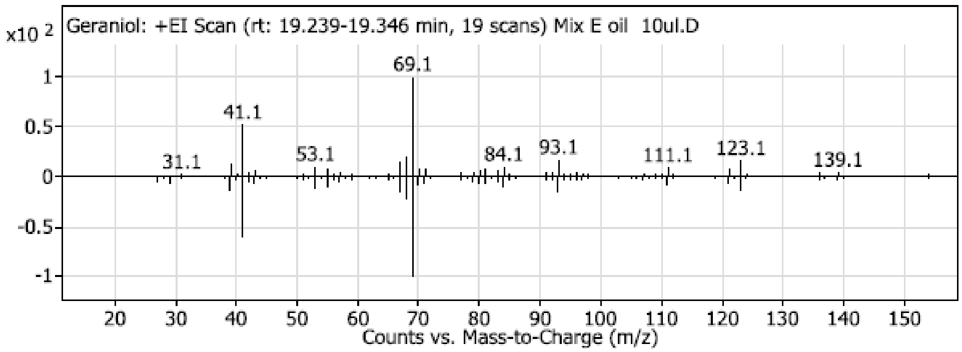

Spectrum Structure

Geraniol

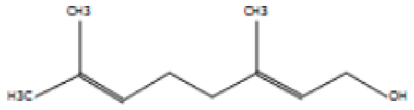

Spectrum Source	Collision Energy	Ionization Mode
Peak (5) in "+ TIC Scan"	0	EI

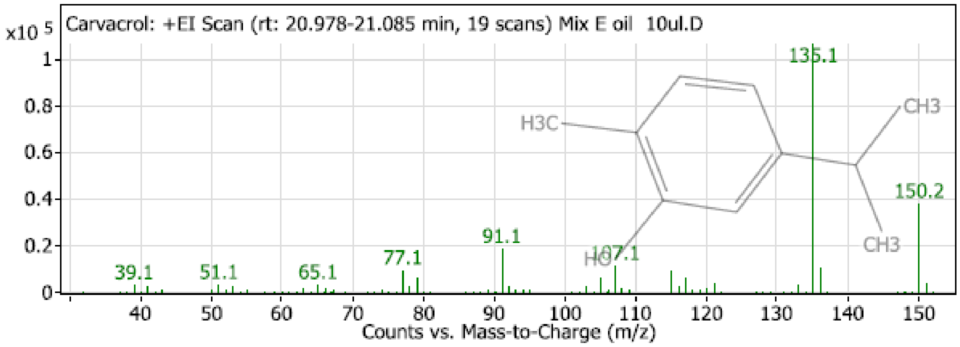

Library Spectrum

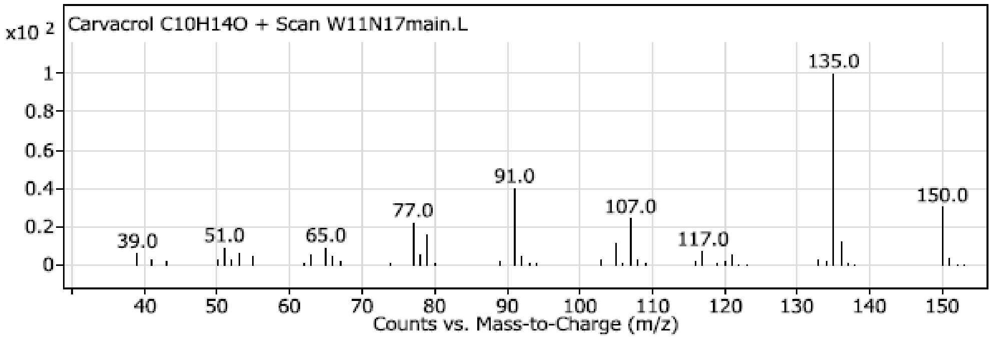

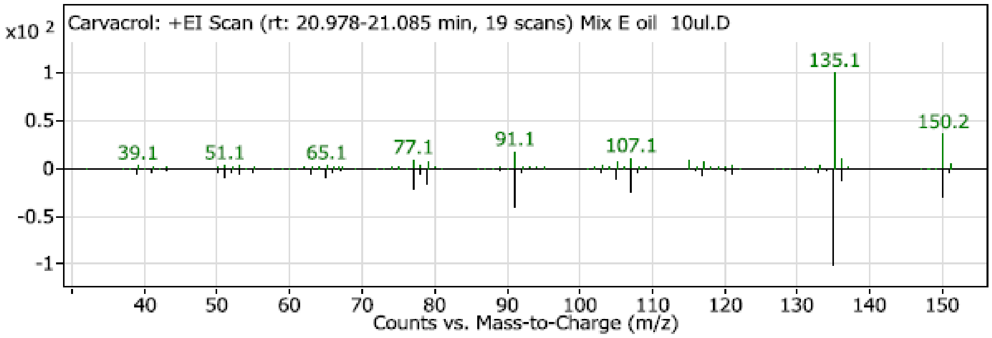

Spectrum Structure

Carvacrol

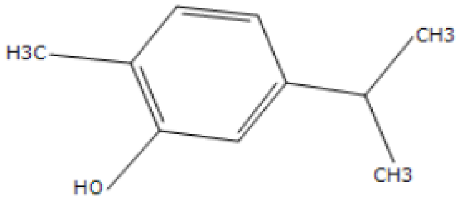

Spectrum Source	Collision Energy	Ionization Mode
Peak (6) in "+ TIC Scan"	0	EI

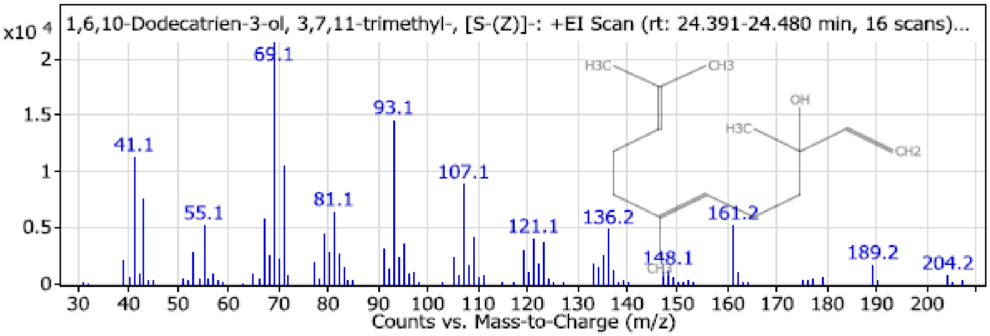

Library Spectrum

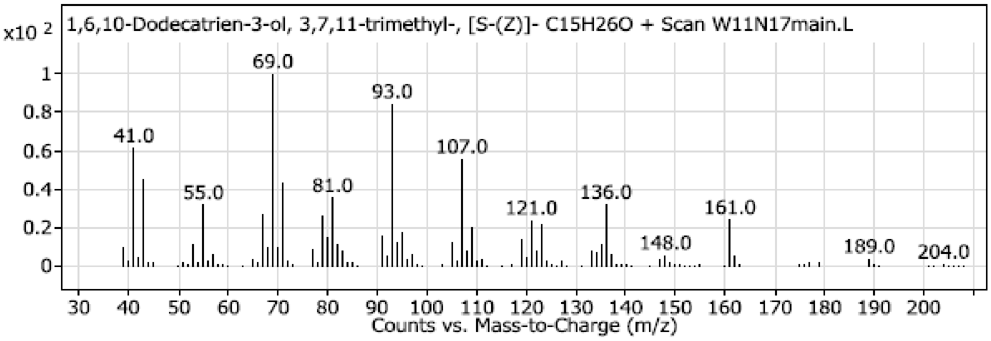

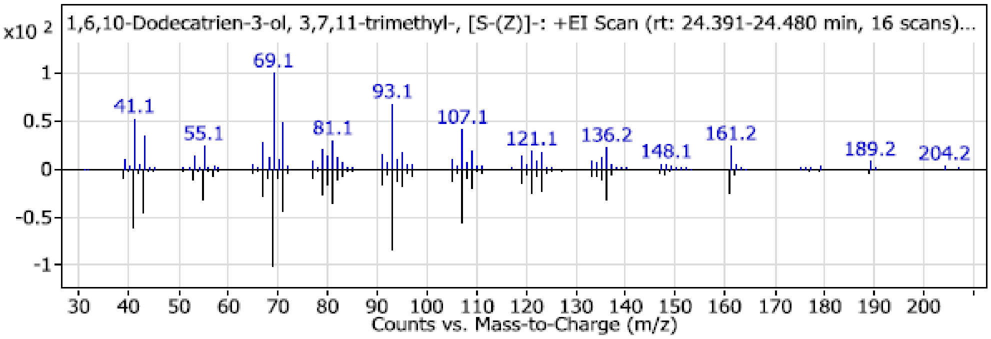

Spectrum Structure

1,6,10-Dodecatrien-3-ol, 3,7,11-trimethyl-, [S-(Z)]-

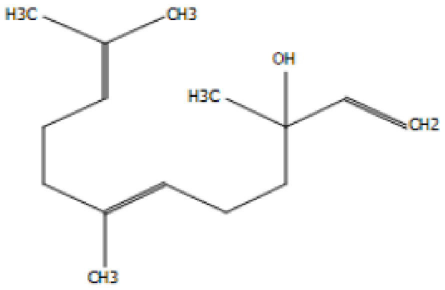

Spectrum Source	Collision Energy	Ionization Mode
Peak (7) in "+ TIC Scan"	0	EI

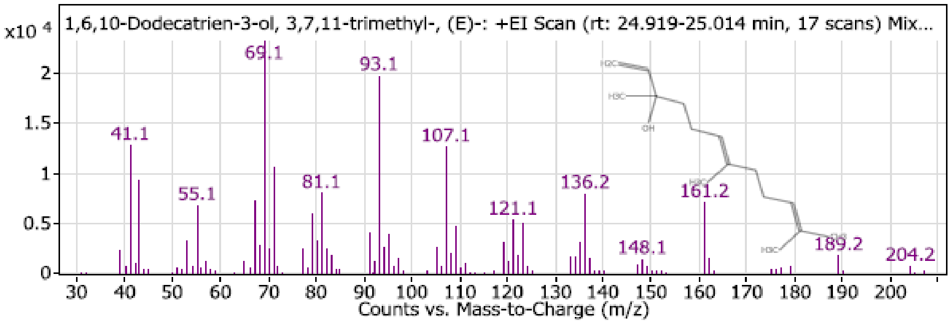

Library Spectrum

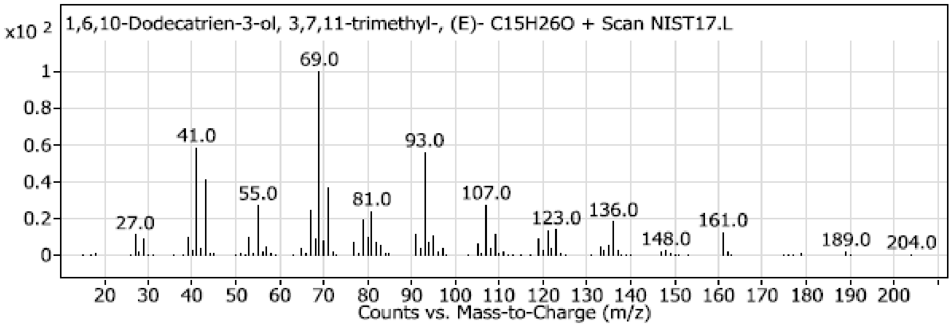

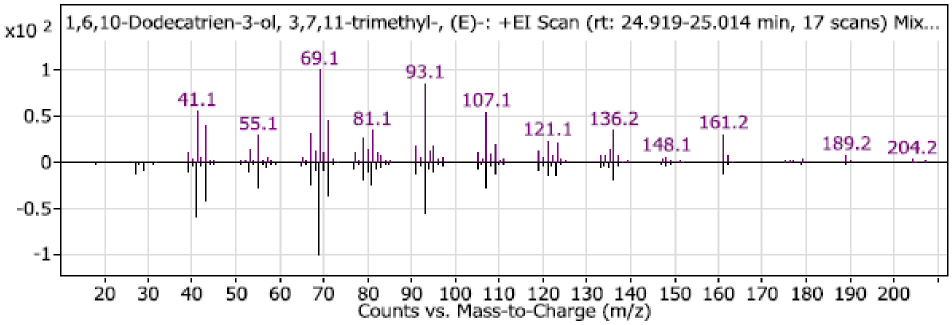

Spectrum Structure

1,6,10-Dodecatrien-3-ol, 3,7,11-trimethyl-, (E)-

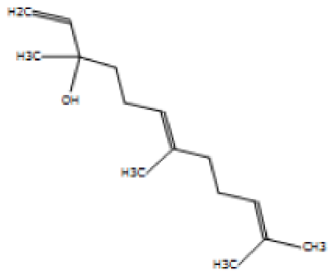

--- End Of Report ---

DRS-(2hr)

Qualitative Analysis Report

**Data Filename**	DRS-1.D	**Sample Name**	DRS-1
**Sample Type**		**Position**	1
**Instrument Name**	Head Space	**User Name**	
**Acq Method**	Essnestial oil DB-624 (He).M	**Acquired Time**	5/29/2023 1:51:03 PM
**IRM Calibration Status**	Not Applicable	**DA Method**	SignalToNoiseCheckout.m
**Comment**			

**Expected Barcode**		**Sample Amount**	
**Dual Inj Vol**	1	**TuneName**	ATUNE.U
**TunePath**	D:\MassHunter\GCMS\1\5977 \	**TuneDateStamp**	2023-05-09T16:01:14+02:00
**MSFirmwareVersion**	6.00.34	**OperatorName**	
**RunCompletedFlag**	True	**Acquisition SW Version**	MassHunter GC/MS Acquisition 10.0.368 14-Feb-2019 Copyright © 1989-2018 Agilent Technologies, Inc

User Chromatograms

Fragmentor Voltage **Collision Energy** 0 **Ionization Mode** EI

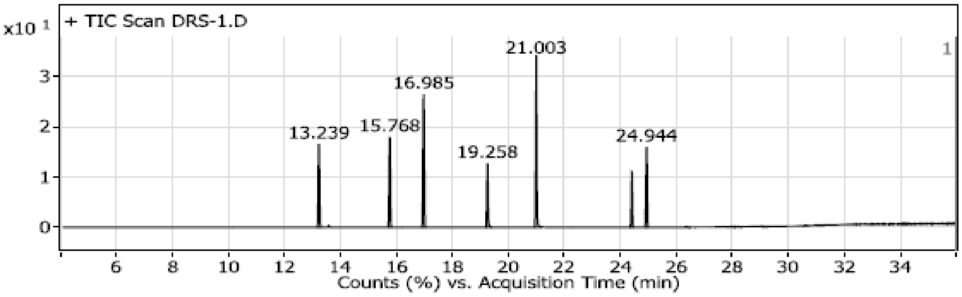

Integration Peak List

Peak	Start	RT	End	Height	Area	Area %
1	13.175	13.239	13.304	69502.79	195193.74	47.05
2	15.709	15.768	15.827	75352.49	213206.8	51.39
3	16.914	16.985	17.05	111116.87	324678.91	78.26
4	19.204	19.258	19.424	53495.51	158403.54	38.18
5	20.938	21.003	21.187	143524.38	414874.08	100
6	24.35	24.416	24.481	46241.92	131032.41	31.58
7	24.885	24.944	25.009	67077.94	188644.32	45.47

User Spectra

Spectrum Source Peak (1) in "+ TIC Scan" **Collision Energy** 0 **Ionization Mode** EI

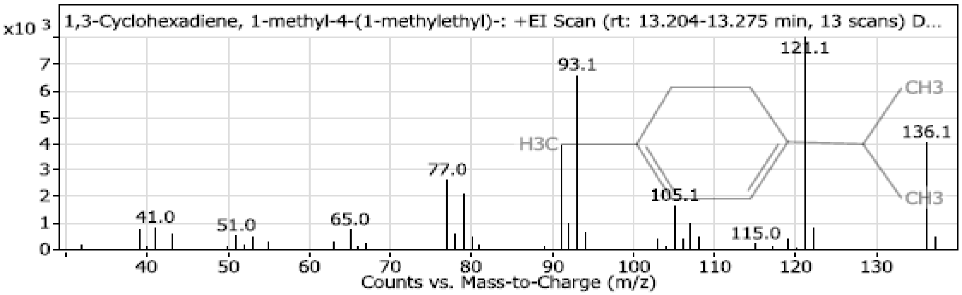

Library Spectrum

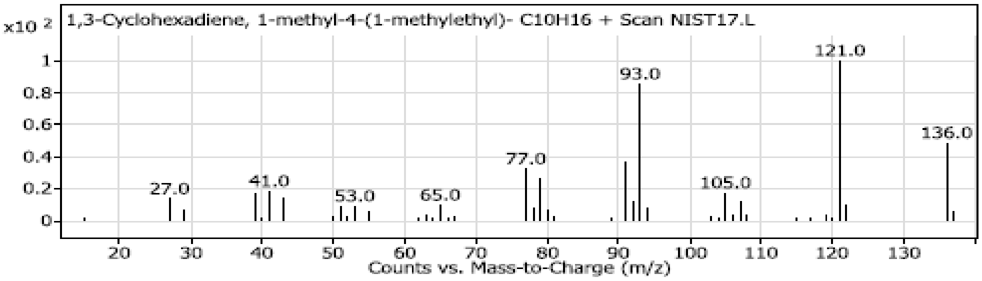

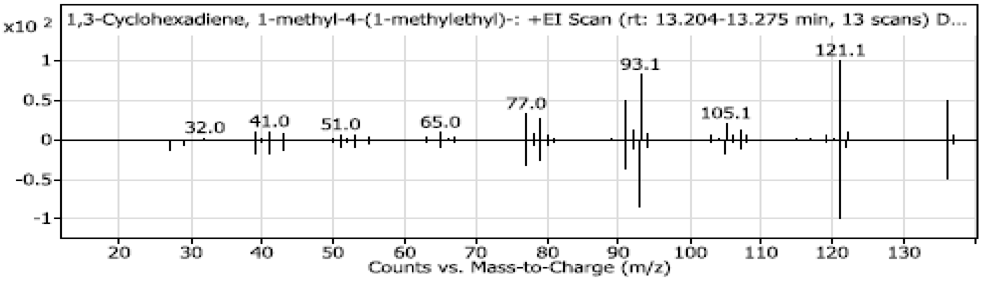

Spectrum Structure

1,3-Cyclohexadiene, 1-methyl-4-(1-methylethyl)-

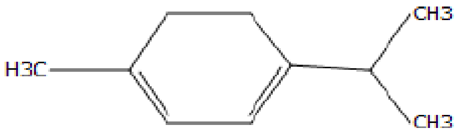

**Spectrum Source**	**Collision Energy**	**Ionization Mode**
Peak (2) in "+ TIC Scan"	0	EI

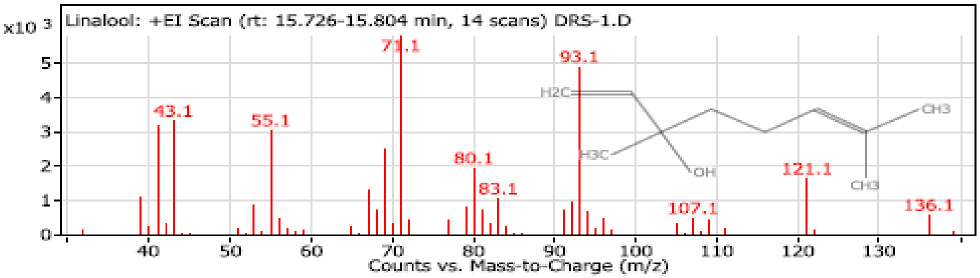

Library Spectrum

Qualitative Analysis Report

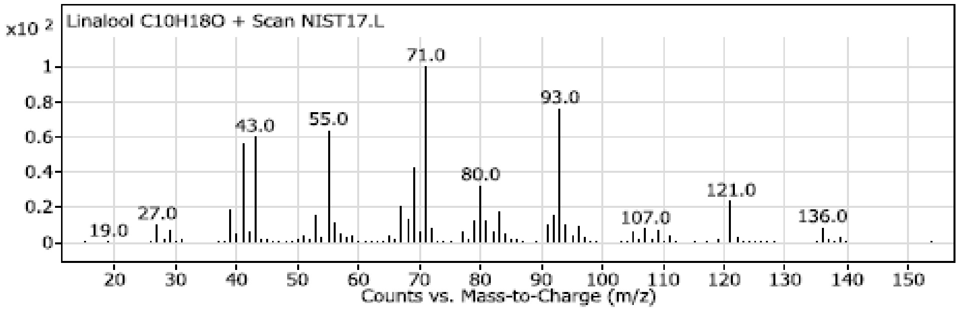

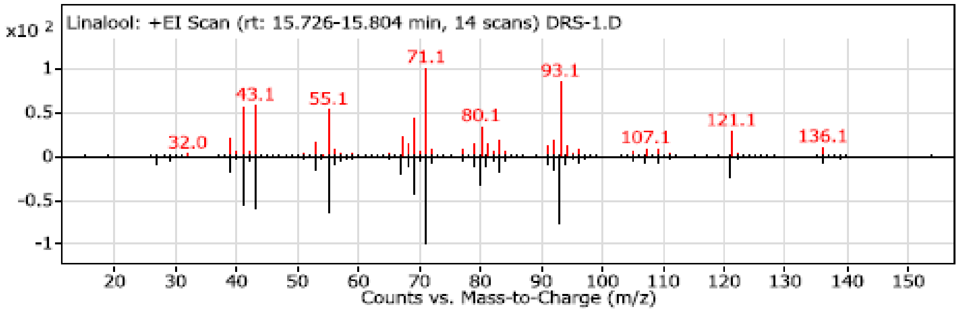

Spectrum Structure
Linalool

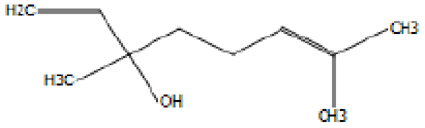

**Spectrum Source**	**Collision Energy**	**Ionization Mode**
Peak (3) in "+ TIC Scan"	0	EI

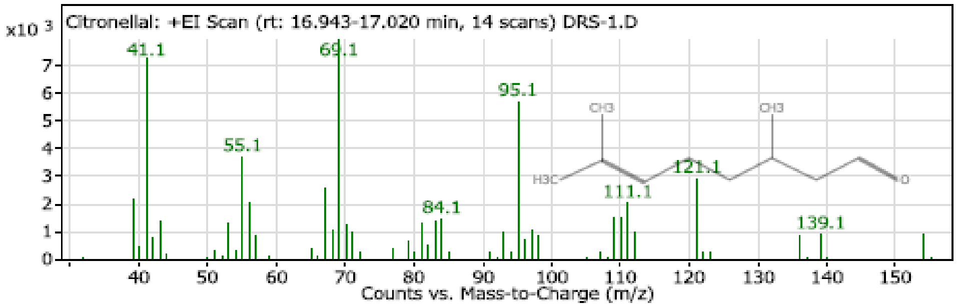

Library Spectrum

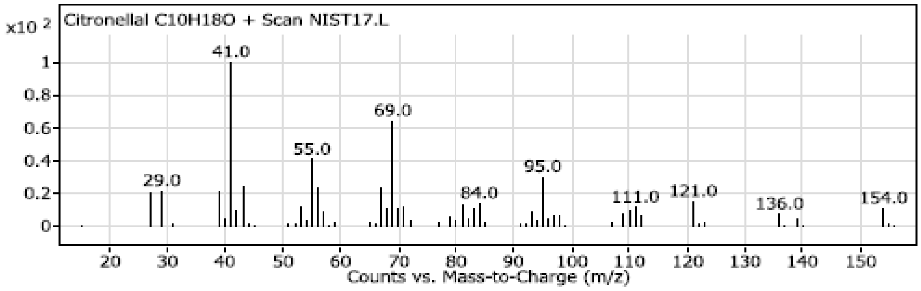

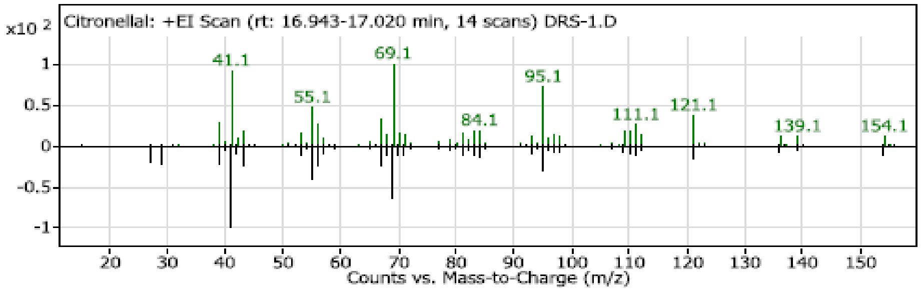

Spectrum Structure

Citronellal

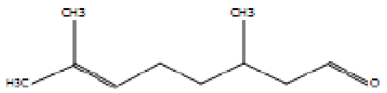

Spectrum Source	Collision Energy	Ionization Mode
Peak (4) in "+ TIC Scan"	0	EI

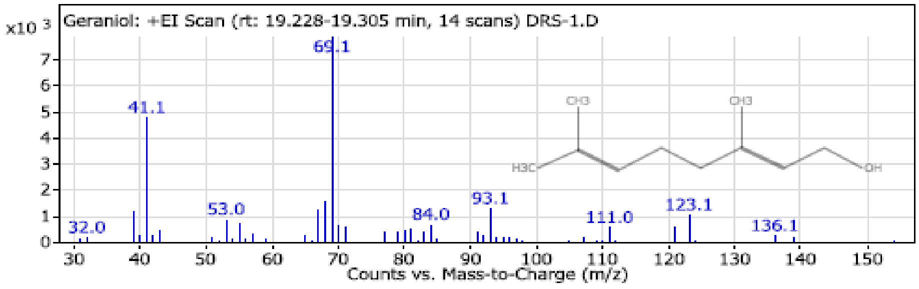

Library Spectrum

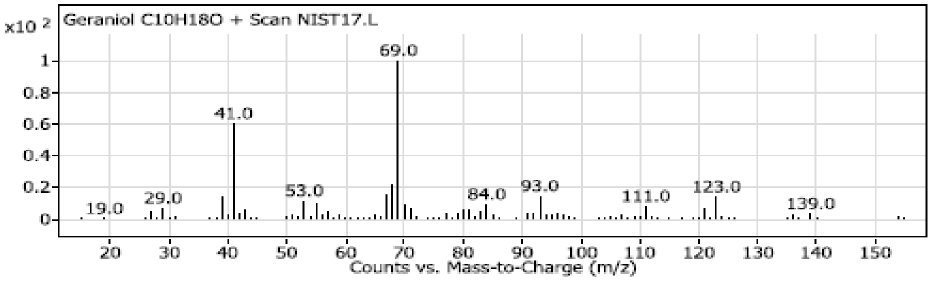

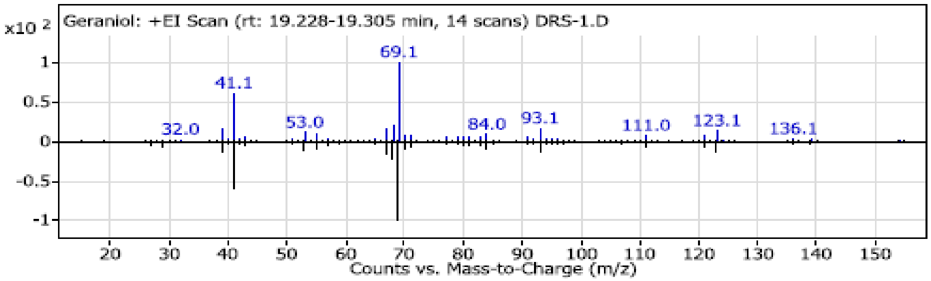

Spectrum Structure
Geraniol

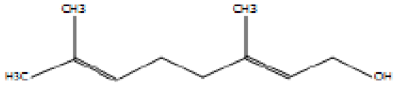

**Spectrum Source**	**Collision Energy**	**Ionization Mode**
Peak (5) in "+ TIC Scan"	0	EI

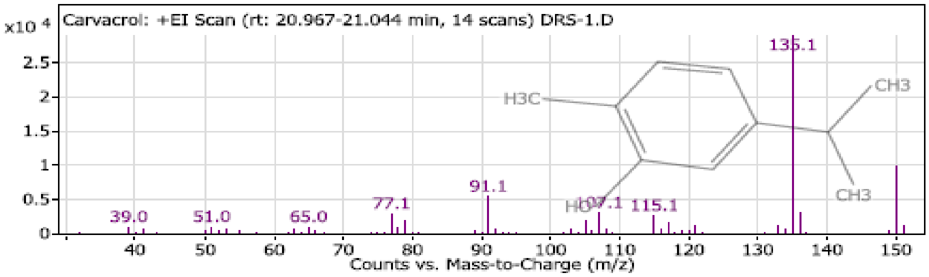

Library Spectrum

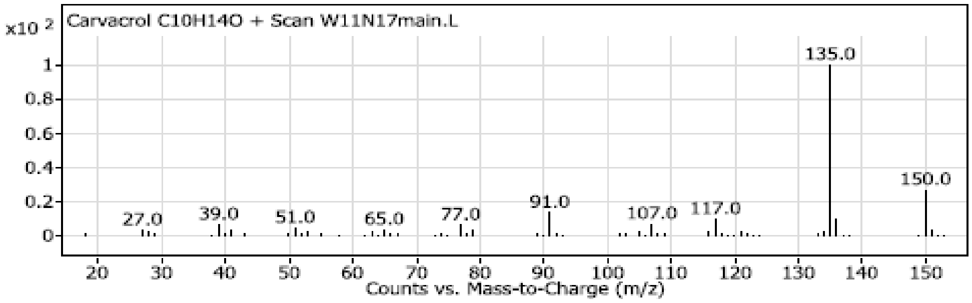

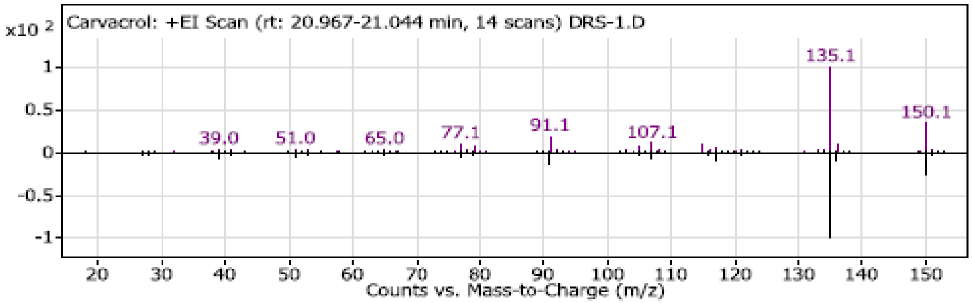

Spectrum Structure

Carvacrol

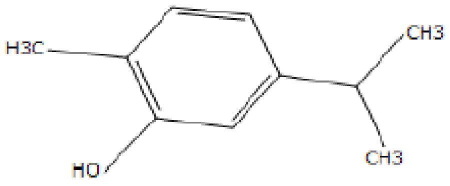

**Spectrum Source**	**Collision Energy**	**Ionization Mode**
Peak (6) in "+ TIC Scan"	0	EI

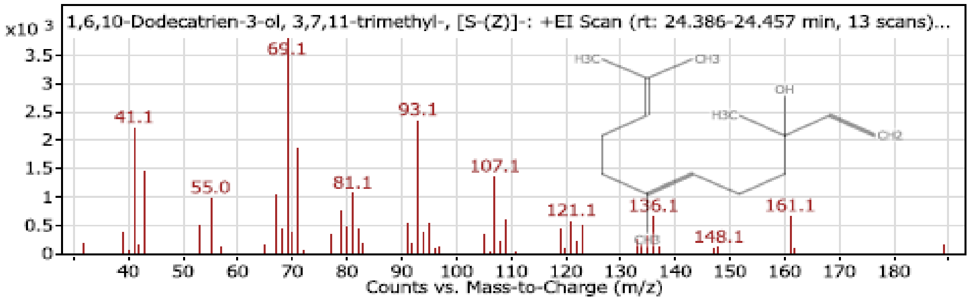

Library Spectrum

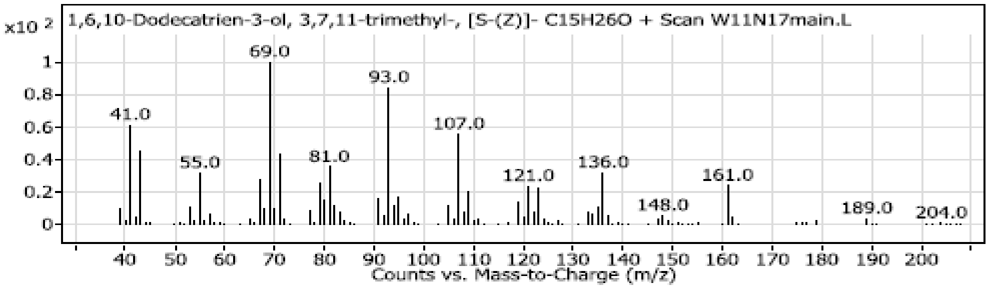

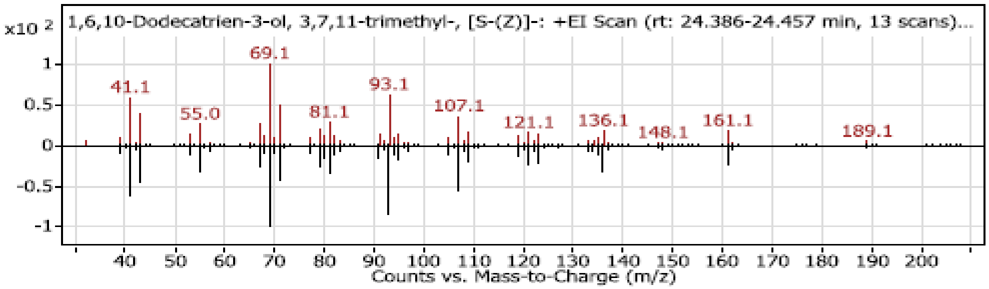

Spectrum Structure

1,6,10-Dodecatrien-3-ol, 3,7,11-trimethyl-, [S-(Z)]-

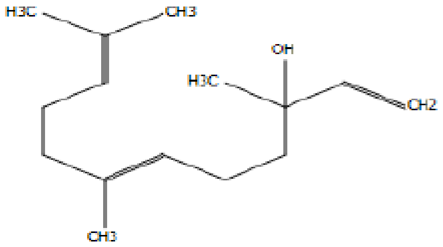

Spectrum Source	Collision Energy	Ionization Mode
Peak (7) in "+ TIC Scan"	0	EI

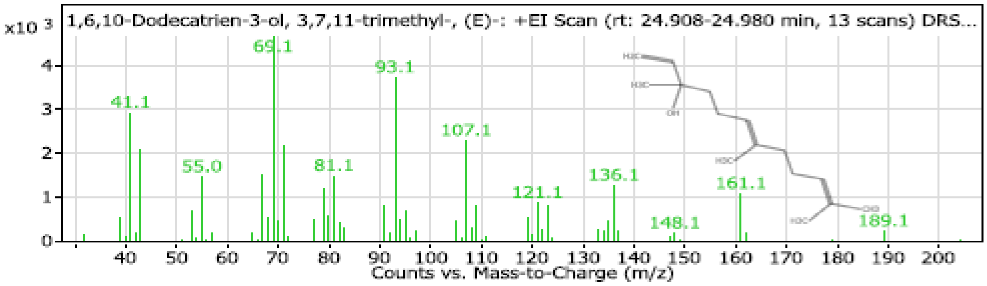

Library Spectrum

Qualitative Analysis Report

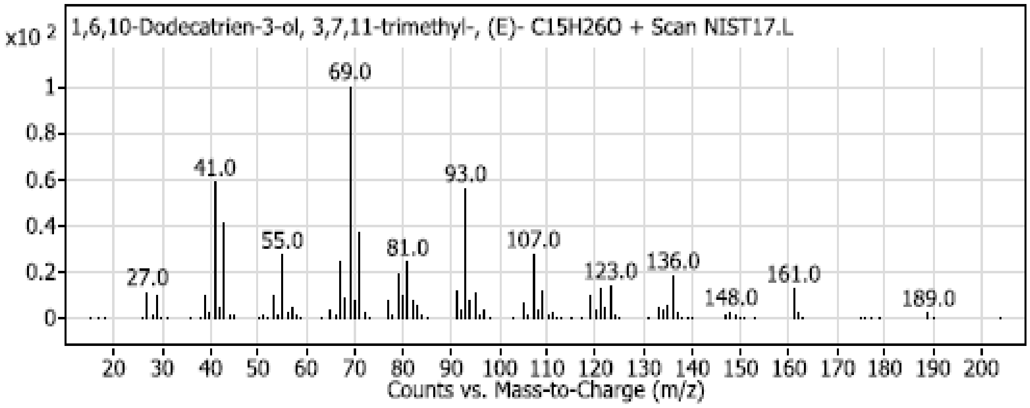

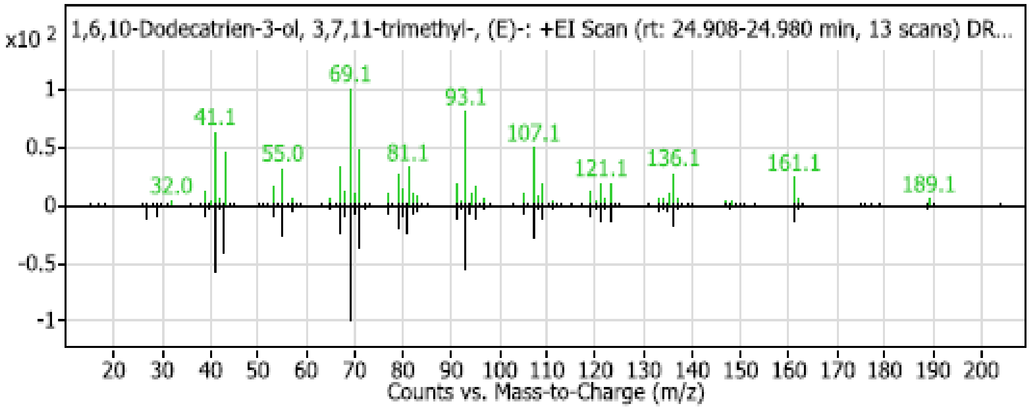

Spectrum Structure

1,6,10-Dodecatrien-3-ol, 3,7,11-trimethyl-, (E)-

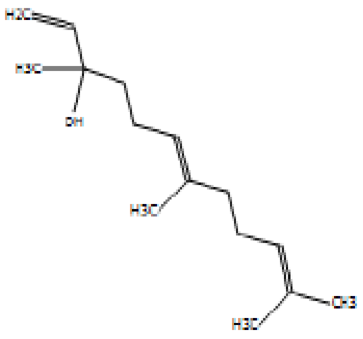

--- End Of Report ---

Drug Release retention time and peak area after2 hr

Peak	RT	Name	Area	Area Sum %	common names
1	13.24	1,3-Cyclohexadiene, 1-methyl-4-(1-methylethyl)-	195194	12	α-Terpinene
2	15.77	Linallol	213207	13.11	Linallol
3	16.99	Citronellal	324679	19.97	Citronellal
4	19.26	Geraniol	158404	9.74	Geraniol
5	21	Carvacrol	414874	25.51	Carvacrol
6	24.42	1,6,10-Dodecatrien-3-ol, 3,7,11-trimethyl-, [S-(Z)]-	131032	8.06	cis-Nerolidol
7	24.94	1,6,10-Dodecatrien-3-ol, 3,7,11-trimethyl-, (E)-	188644	11.6	trans-Nerolidol

DRS-(4hr)

Qualitative Analysis Report

**Data Filename**	DRS-2.D	**Sample Name**	DRS-2
**Sample Type**		**Position**	1
**Instrument Name**	Head Space	**User Name**	
**Acq Method**	Essnestial oil DB-624 (He).M	**Acquired Time**	5/29/2023 2:46:14 PM
**IRM Calibration Status**	Not Applicable	**DA Method**	SignalToNoiseCheckout.m
**Comment**			

**Expected Barcode**		**Sample Amount**	
**Dual Inj Vol**	1	**TuneName**	ATUNE.U
**TunePath**	D:\MassHunter\GCMS\1\5977 \	**TuneDateStamp**	2023-05-09T16:01:14+02:00
**MSFirmwareVersion**	6.00.34	**OperatorName**	
**RunCompletedFlag**	True	**Acquisition SW Version**	MassHunter GC/MS Acquisition 10.0.368 14-Feb-2019 Copyright © 1989-2018 Agilent Technologies, Inc

User Chromatograms

Fragmentor Voltage **Collision Energy** 0 **Ionization Mode** EI

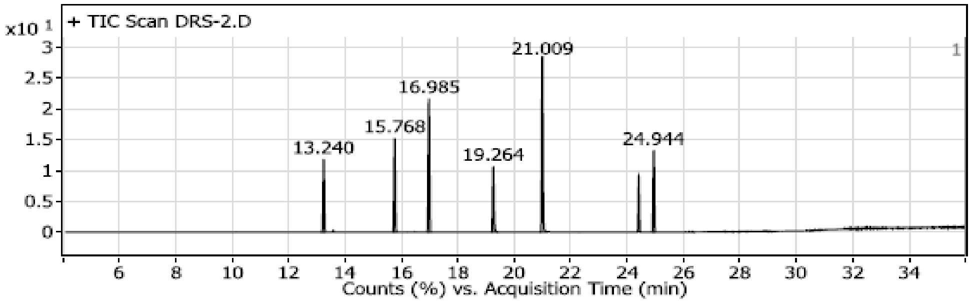

Integration Peak List

Peak	Start	RT	End	Height	Area	Area %
1	13.186	13.24	13.299	49508.02	135805.22	39.84
2	15.709	15.768	15.827	63874.08	179492.31	52.65
3	16.92	16.985	17.05	90780.25	262069.01	76.88
4	19.205	19.264	19.401	44801.73	129645.67	38.03
5	20.926	21.009	21.157	119572.42	340894.07	100
6	24.361	24.422	24.475	38257.78	107792.99	31.62
7	24.882	24.944	25.003	55608.77	157066.45	46.07

User Spectra

Spectrum Source	Collision Energy	Ionization Mode
Peak (1) in "+ TIC Scan"	0	EI

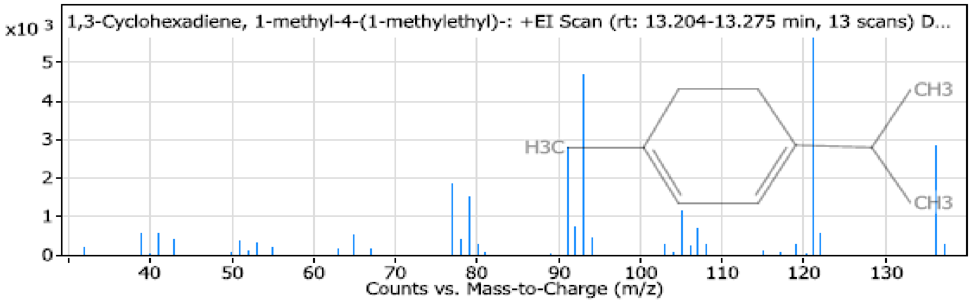

Library Spectrum

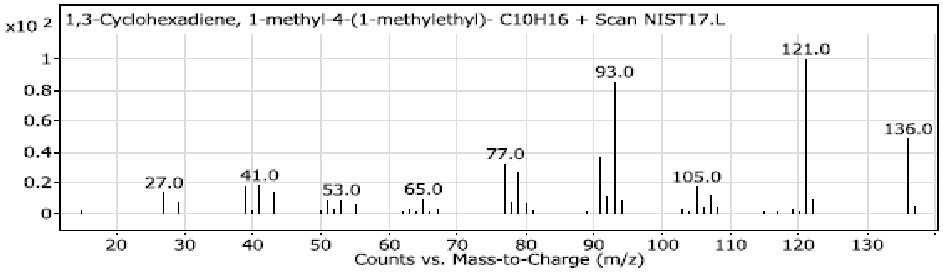

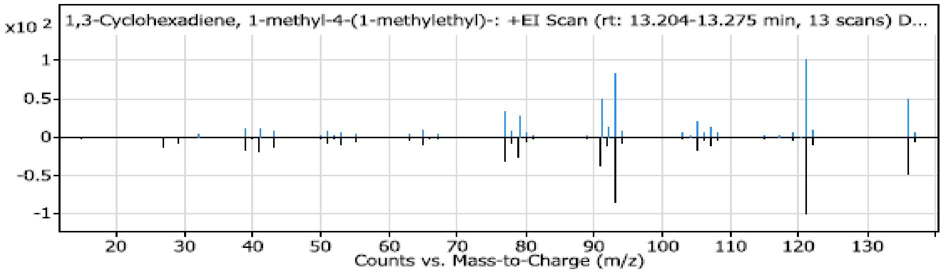

Spectrum Structure

1,3-Cyclohexadiene, 1-methyl-4-(1-methylethyl)-

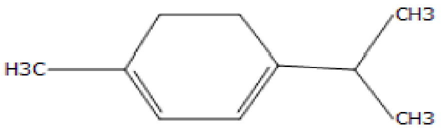

**Spectrum Source**	**Collision Energy**	**Ionization Mode**
Peak (2) in "+ TIC Scan"	0	EI

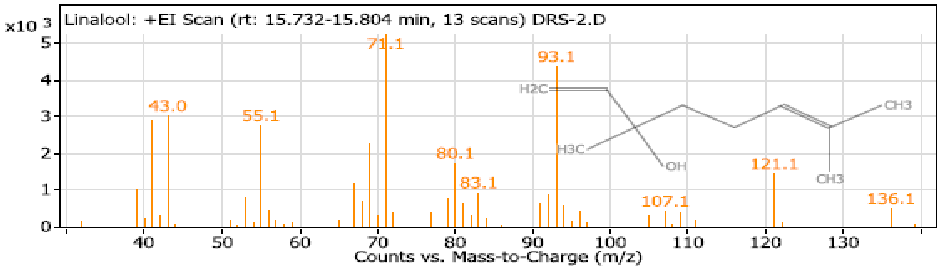

Library Spectrum

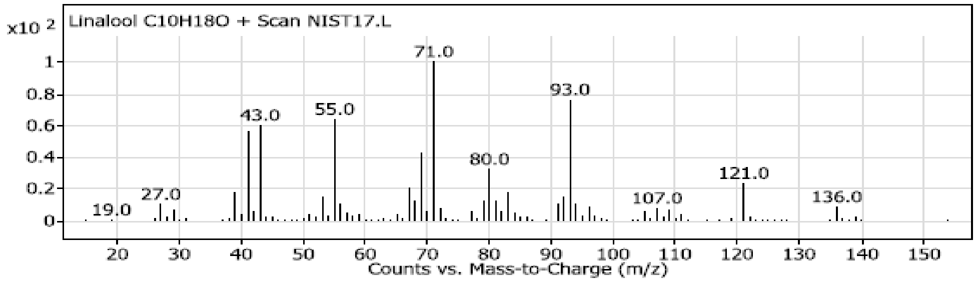

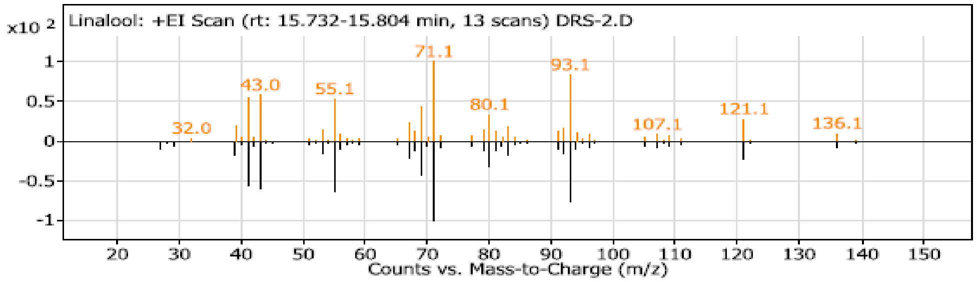

Spectrum Structure

Linalool

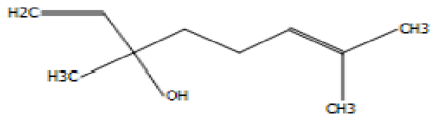

**Spectrum Source**	**Collision Energy**	**Ionization Mode**
Peak (3) in "+ TIC Scan"	0	EI

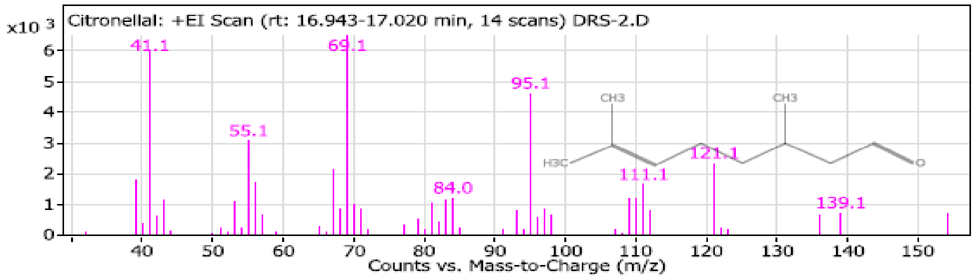

Library Spectrum

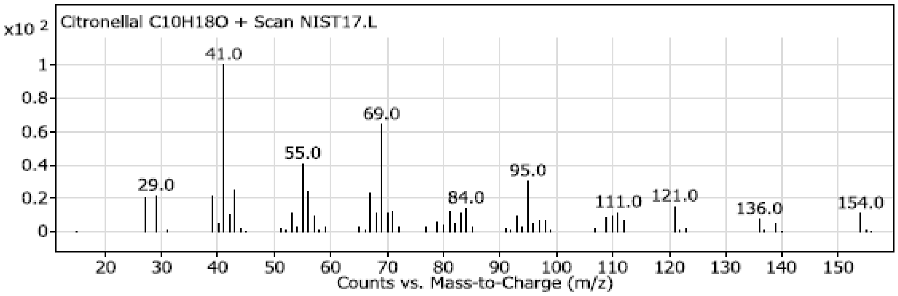

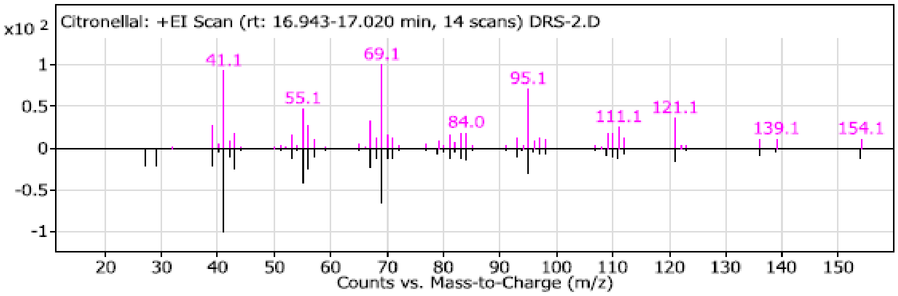

Spectrum Structure

Citronellal

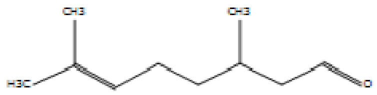

Spectrum Source	Collision Energy	Ionization Mode
Peak (4) in "+ TIC Scan"	0	EI

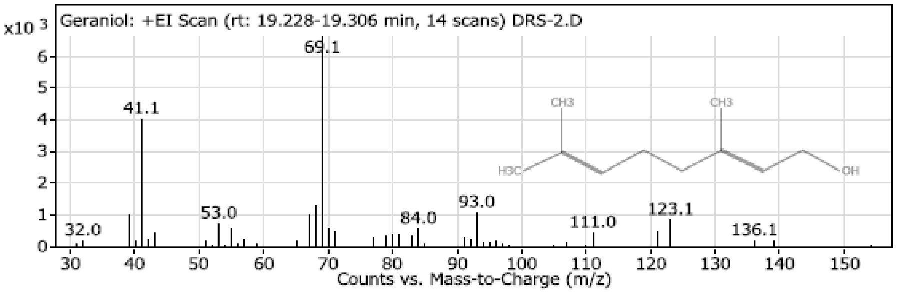

Library Spectrum

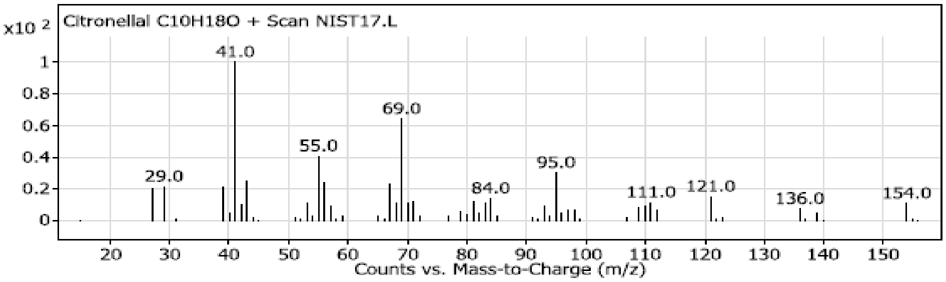

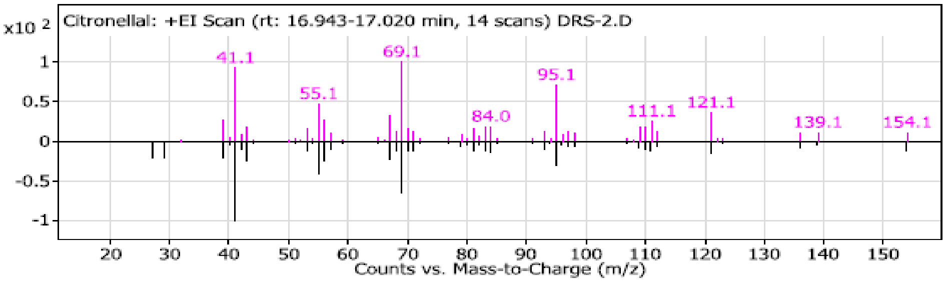

Spectrum Structure
Citronellal

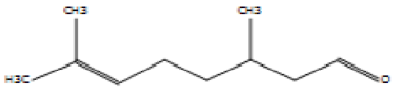

**Spectrum Source**	**Collision Energy**	**Ionization Mode**
Peak (4) in "+ TIC Scan"	0	EI

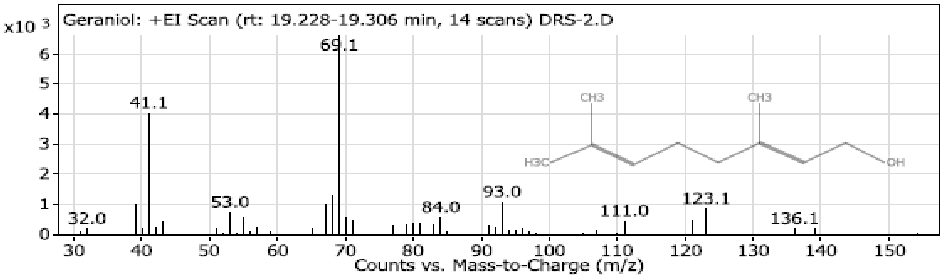

Library Spectrum

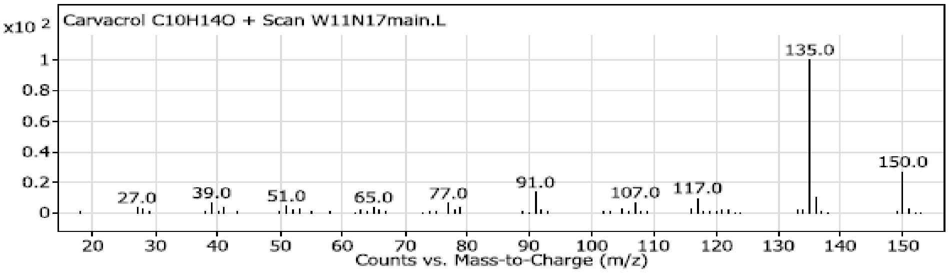

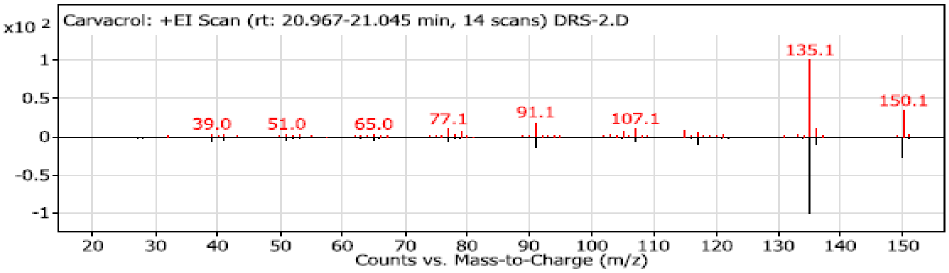

Spectrum Structure

Carvacrol

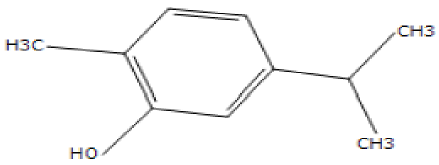

Spectrum Source	Collision Energy	Ionization Mode
Peak (6) in "+ TIC Scan"	0	EI

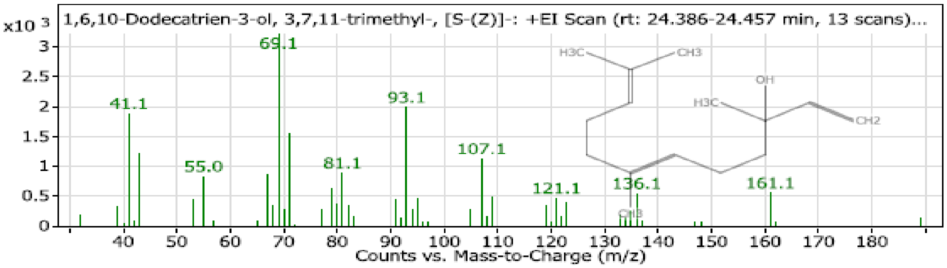

Library Spectrum

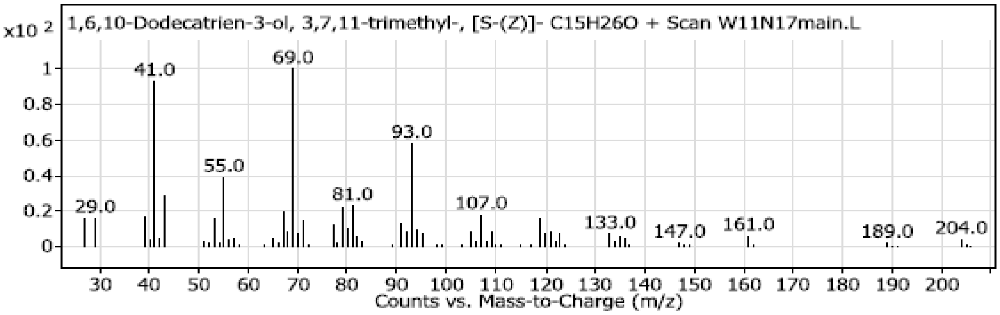

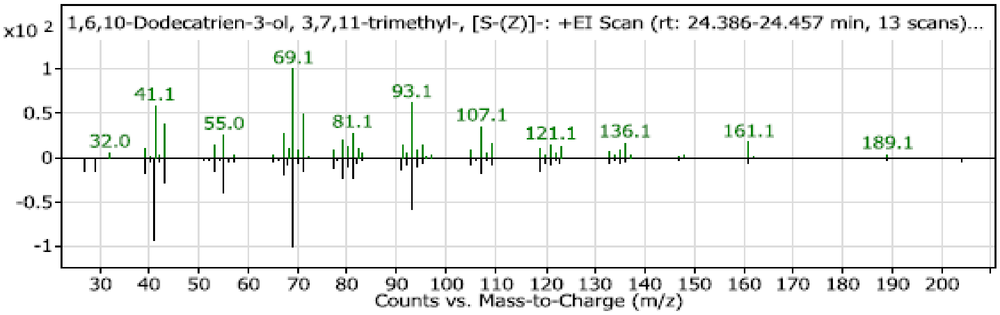

Spectrum Structure

1,6,10-Dodecatrien-3-ol, 3,7,11-trimethyl-, [S-(Z)]-

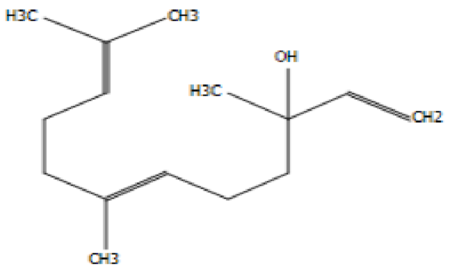

Spectrum Source	Collision Energy	Ionization Mode
Peak (7) in "+ TIC Scan"	0	EI

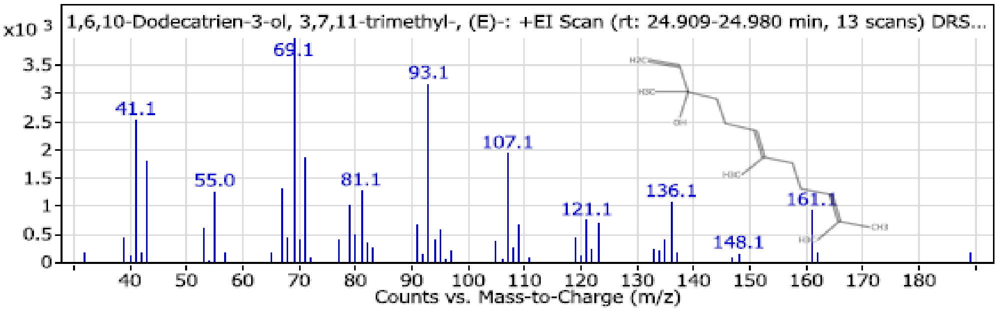

Library Spectrum

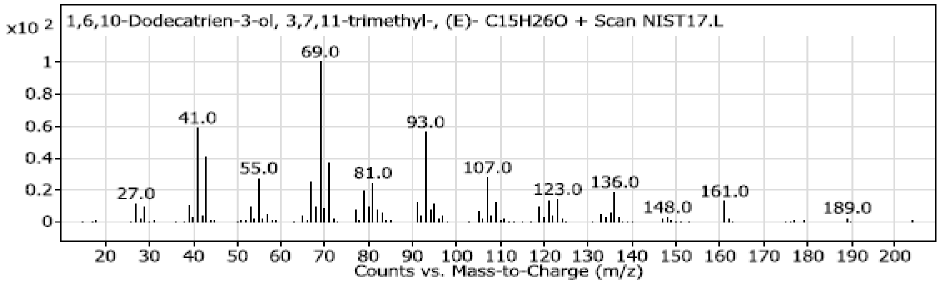

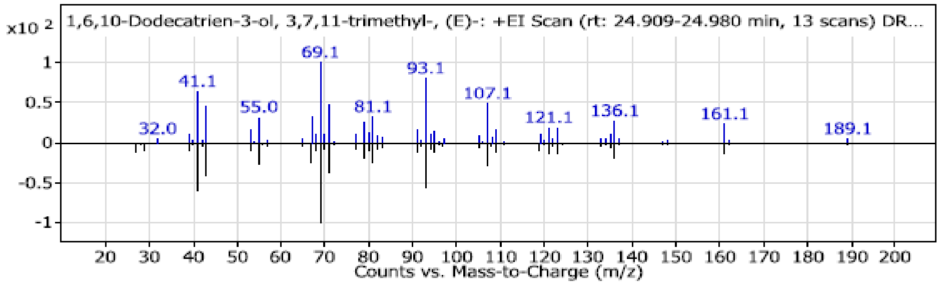

Spectrum Structure

1,6,10-Dodecatrien-3-ol, 3,7,11-trimethyl-, (E)-

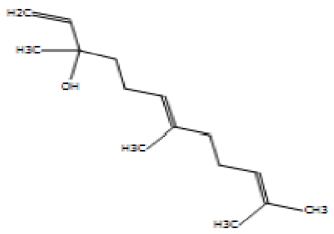

--- End Of Report ---

Drug Release retention time and peak area after 4 hr

Peak	RT	Name	Area	Area Sum %	
1	13.24	1,3-Cyclohexadiene, 1-methyl-4-(1-methylethyl)-	135805	10.34	α-Terpinene
2	15.77	Linalool	179492	13.67	Linalool
3	16.99	Citronellal	262069	19.96	Citronellal
4	19.26	Geraniol	129646	9.88	Geraniol
5	21.01	Carvacrol	340894	25.97	Carvacrol
6	24.42	1,6,10-Dodecatrien-3-ol, 3,7,11-trimethyl-, [S-(Z)]-	107793	8.21	cis-Nerolidol
7	24.94	1,6,10-Dodecatrien-3-ol, 3,7,11-trimethyl-, (E)-	157066	11.96	trans-Nerolidol

DRS-(6hr)

Qualitative Analysis Report

**Data Filename**	DRS-3.D	**Sample Name**	DRS-3
**Sample Type**		**Position**	2
**Instrument Name**	Head Space	**User Name**	
**Acq Method**	Essnestial oil DB-624 (He).M	**Acquired Time**	5/29/2023 3:30:07 PM
**IRM Calibration Status**	Not Applicable	**DA Method**	SignalToNoiseCheckout.m
**Comment**			

**Expected Barcode**		**Sample Amount**	
**Dual Inj Vol**	1	**TuneName**	ATUNE.U
**TunePath**	D:\MassHunter\GCMS\1\5977 \	**TuneDateStamp**	2023-05-09T16:01:14+02:00
**MSFirmwareVersion**	6.00.34	**OperatorName**	
**RunCompletedFlag**	True	**Acquisition SW Version**	MassHunter GC/MS Acquisition 10.0.368 14-Feb-2019 Copyright © 1989-2018 Agilent Technologies, Inc

User Chromatograms

Fragmentor Voltage **Collision Energy** 0 **Ionization Mode** EI

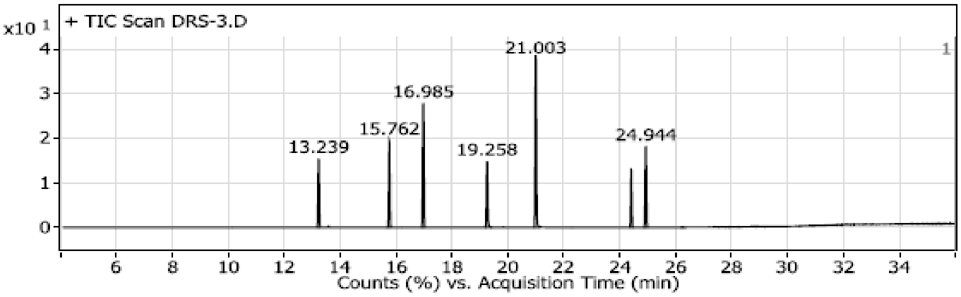

Integration Peak List

Peak	Start	RT	End	Height	Area	Area %
1	13.169	13.239	13.299	64905.53	181159.19	38.84
2	15.703	15.762	15.83	82106.31	240873.77	51.64
3	16.903	16.985	17.051	116968.97	346654.07	74.31
4	19.199	19.258	19.429	62450.11	185151.64	39.69
5	20.938	21.003	21.139	161799.8	466466.67	100
6	24.362	24.416	24.481	55350.75	154760.01	33.18
7	24.885	24.944	25.01	76395.62	221346.1	47.45

User Spectra

Spectrum Source	Collision Energy	Ionization Mode
Peak (1) in "+ TIC Scan"	0	EI

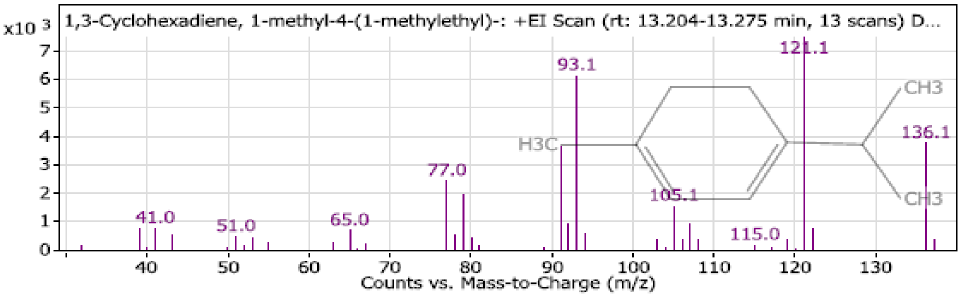

Library Spectrum

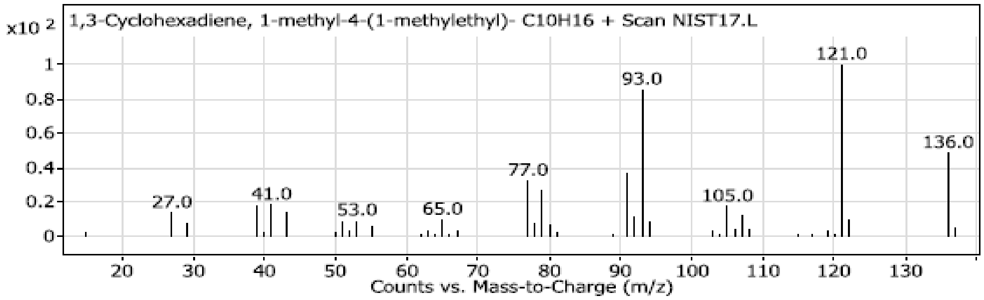

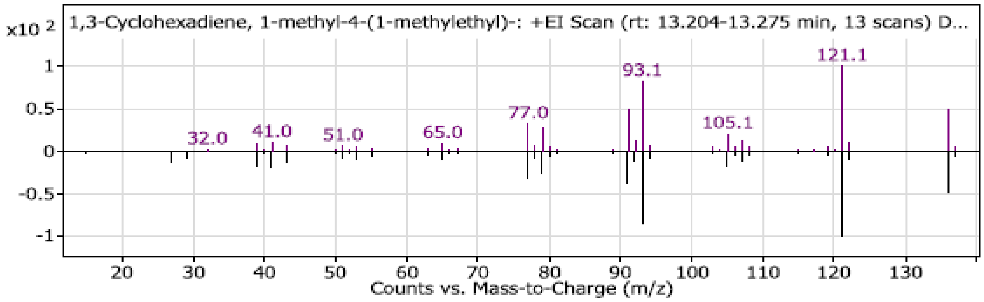

Spectrum Structure

1,3-Cyclohexadiene, 1-methyl-4-(1-methylethyl)-

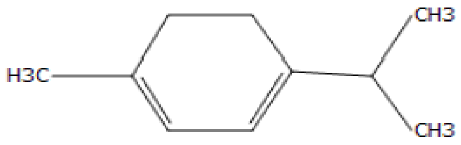

Spectrum Source	Collision Energy	Ionization Mode
Peak (2) in "+ TIC Scan"	0	EI

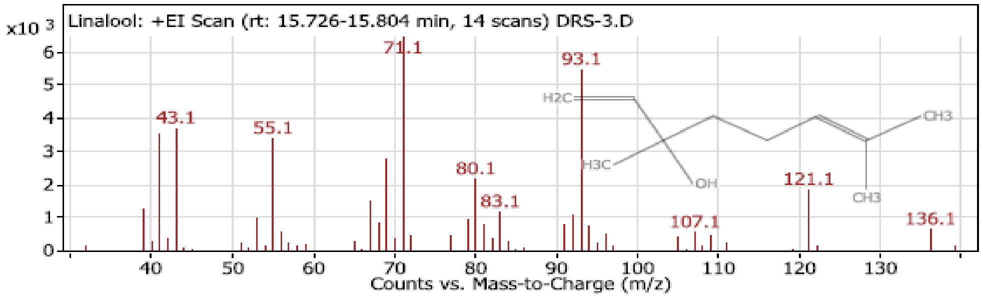

Library Spectrum

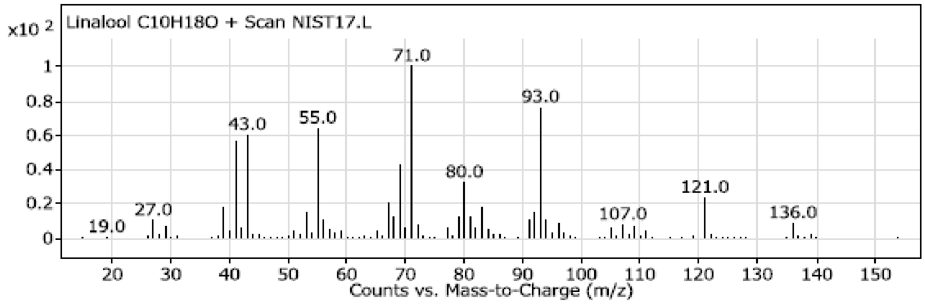

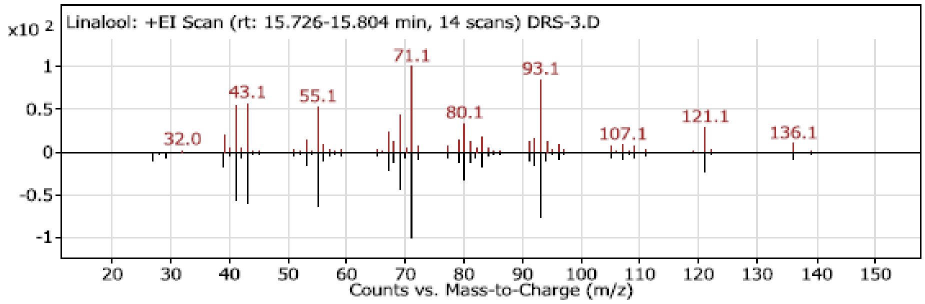

Spectrum Structure

Linalool

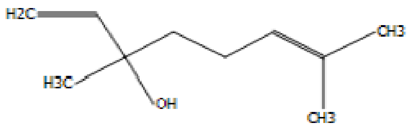

**Spectrum Source**	**Collision Energy**	**Ionization Mode**
Peak (3) in "+ TIC Scan"	0	EI

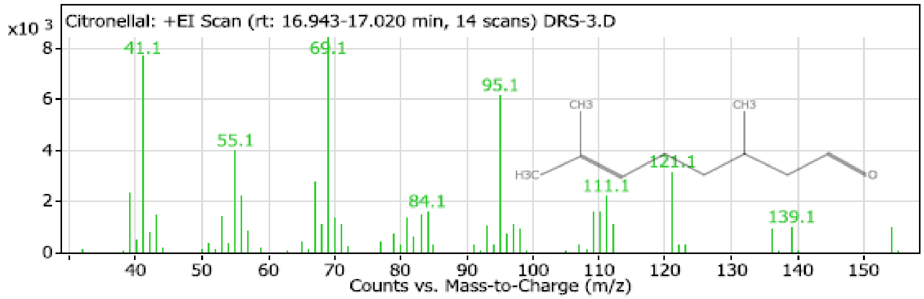

Library Spectrum

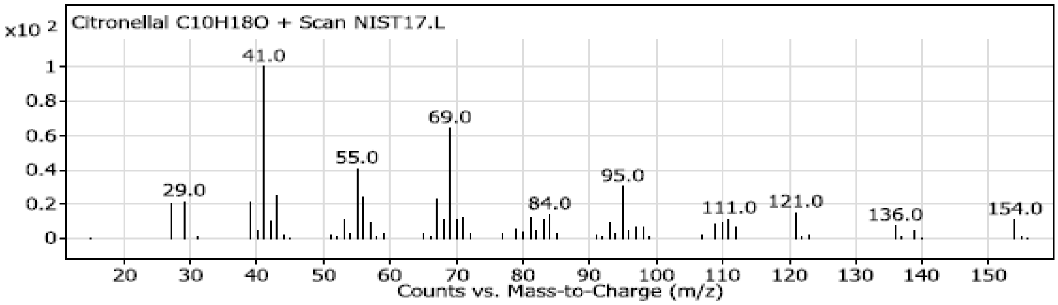

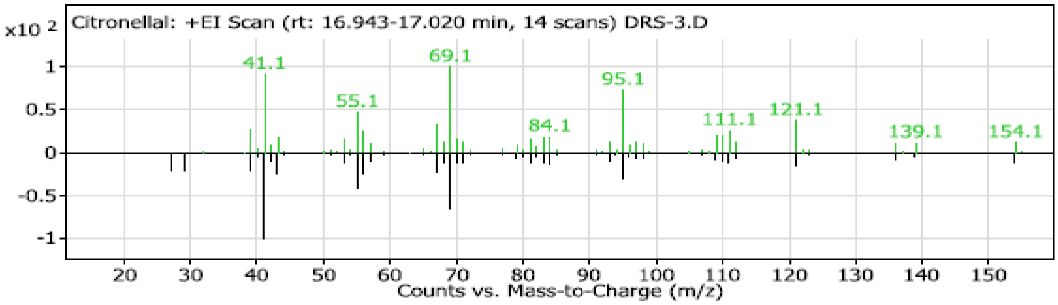

Spectrum Structure

Citronellal

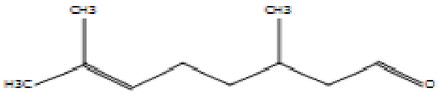

Spectrum Source	Collision Energy	Ionization Mode
Peak (4) in "+ TIC Scan"	0	EI

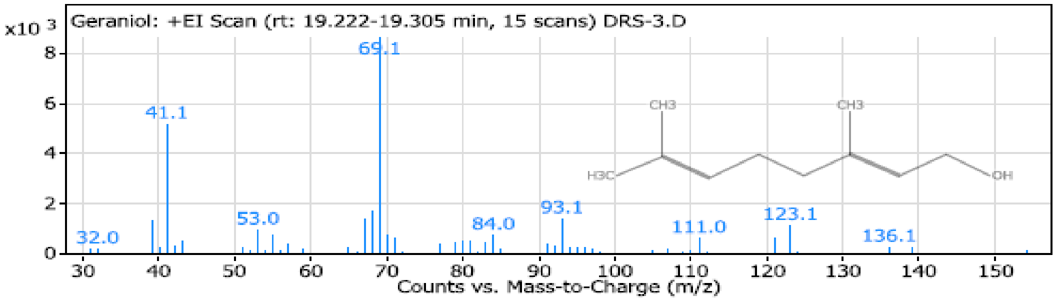

Library Spectrum

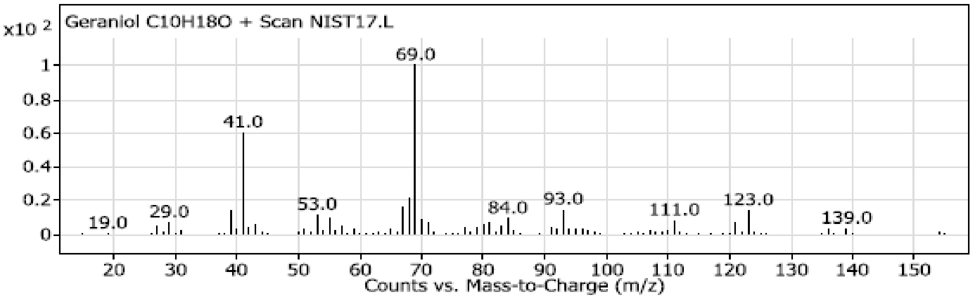

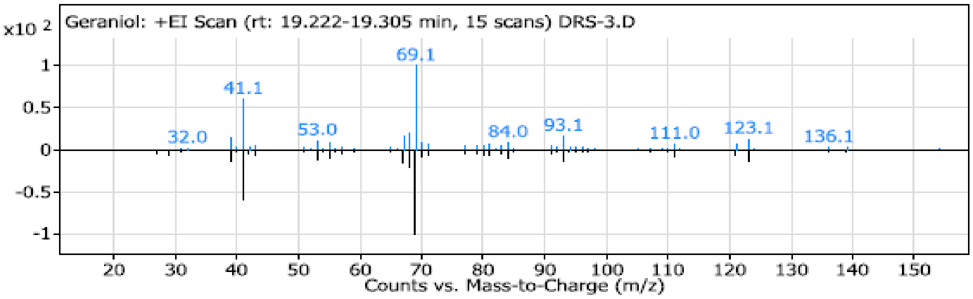

Spectrum Structure
Geraniol

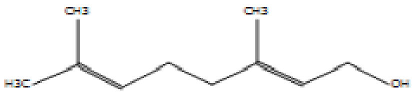

Spectrum Source	Collision Energy	Ionization Mode
Peak (5) in "+ TIC Scan"	0	EI

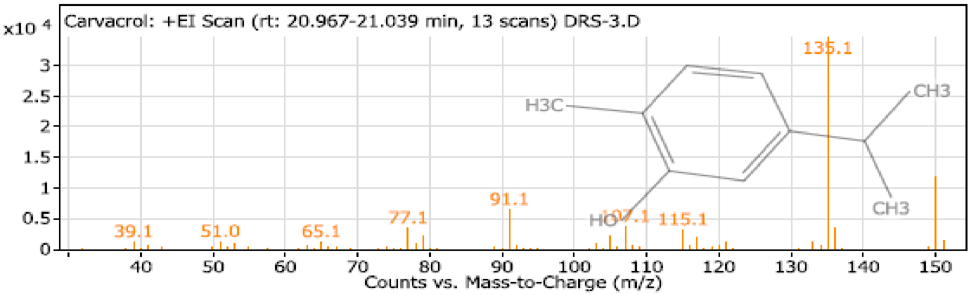

Library Spectrum

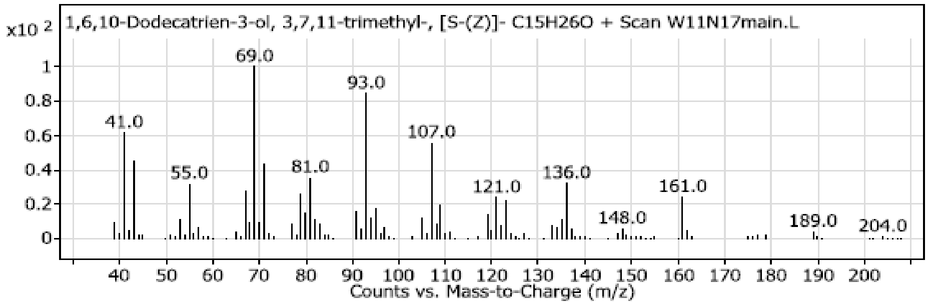

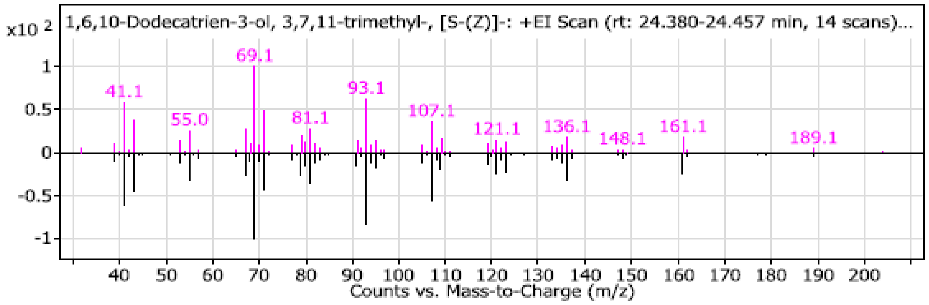

Spectrum Structure

1,6,10-Dodecatrien-3-ol, 3,7,11-trimethyl-, [S-(Z)]-

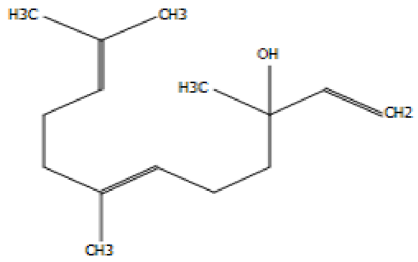

Spectrum Source	Collision Energy	Ionization Mode
Peak (7) in "+ TIC Scan"	0	EI

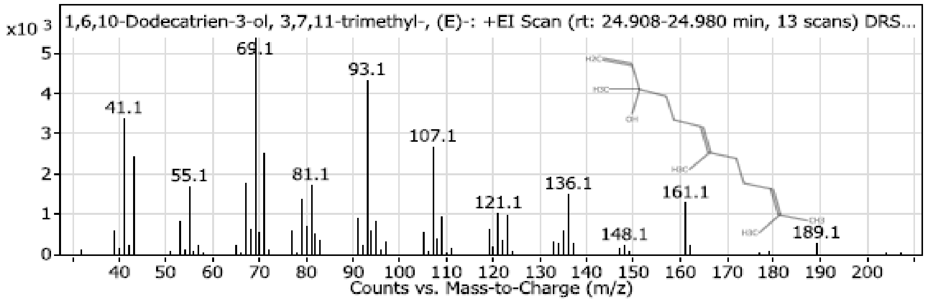

Library Spectrum

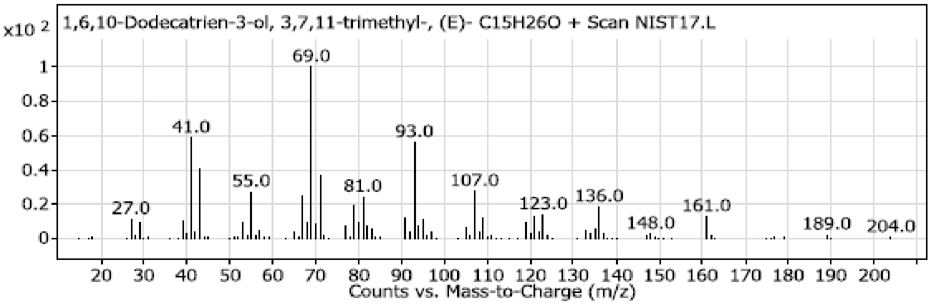

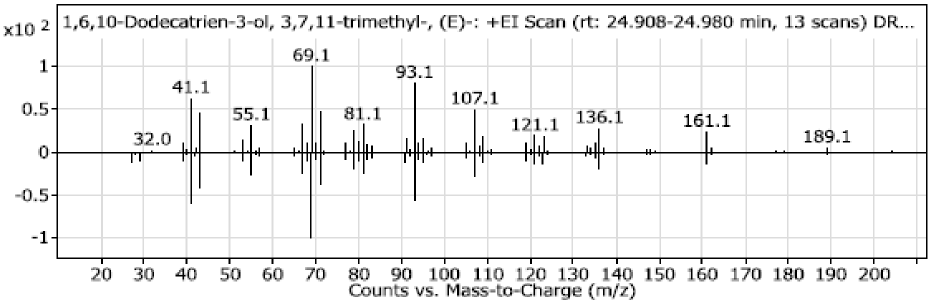

Spectrum Structure

1,6,10-Dodecatrien-3-ol, 3,7,11-trimethyl-, (E)-

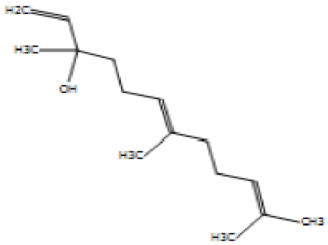

--- End Of Report ---

Drug Release retention time and peak area after 6 hr

Peak	RT	Name	Area	Area Sum %	
1	13.24	1,3-Cyclohexadiene, 1-methyl-4-(1-methylethyl)-	181159	10.08	α-Terpinene
2	15.76	Linallol	240874	13.41	
3	16.99	Citronellal	346654	19.3	
4	19.26	Geraniol	185152	10.31	
5	21	Carvacrol	466467	25.97	
6	24.42	1,6,10-Dodecatrien-3-ol, 3,7,11-trimethyl-, [S-(Z)]-	154760	8.61	cis-Nerolidol
7	24.94	1,6,10-Dodecatrien-3-ol, 3,7,11-trimethyl-, (E)-	221346	12.32	trans-Nerolidol

DRS-(8hr)

Qualitative Analysis Report

**Data Filename**	DRS-4.D	**Sample Name**	DRS-4
**Sample Type**		**Position**	3
**Instrument Name**	Head Space	**User Name**	
**Acq Method**	Essnestial oil DB-624 (He).M	**Acquired Time**	5/29/2023 4:14:04 PM
**IRM Calibration Status**	Not Applicable	**DA Method**	SignalToNoiseCheckout.m
**Comment**			

**Expected Barcode**		**Sample Amount**	
**Dual Inj Vol**	1	**TuneName**	ATUNE.U
**TunePath**	D:\MassHunter\GCMS\1\5977 \	**TuneDateStamp**	2023-05-09T16:01:14+02:00
**MSFirmwareVersion**	6.00.34	**OperatorName**	
**RunCompletedFlag**	True	**Acquisition SW Version**	MassHunter GC/MS Acquisition 10.0.368 14-Feb-2019 Copyright © 1989-2018 Agilent Technologies, Inc

User Chromatograms

Fragmentor Voltage **Collision Energy** 0 **Ionization Mode** EI

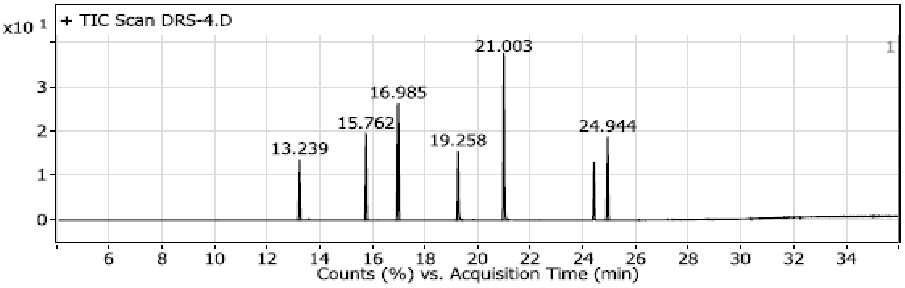

Integration Peak List

Peak	Start	RT	End	Height	Area	Area %
1	13.174	13.239	13.299	57050.46	156186.39	34.09
2	15.703	15.762	15.827	81311.78	234258.93	51.13
3	16.919	16.985	17.05	110171.86	322544.01	70.4
4	19.199	19.258	19.43	64977.41	193169.99	42.16
5	20.938	21.003	21.181	156857.89	458190.42	100
6	24.357	24.422	24.481	54655.85	157784.5	34.44
7	24.875	24.944	25.009	78438.31	225823.09	49.29

User Spectra

Spectrum Source	Collision Energy	Ionization Mode
Peak (1) in "+ TIC Scan"	0	EI

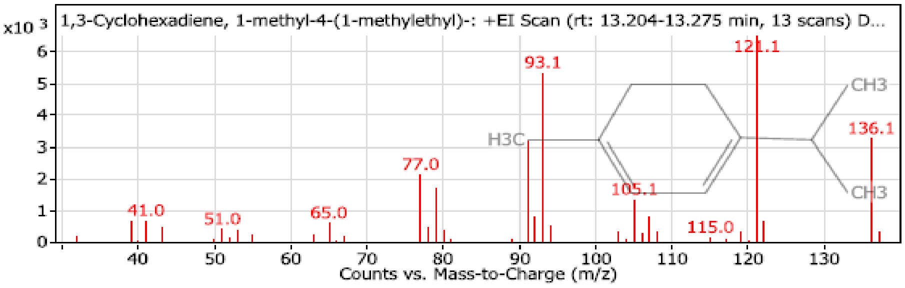

Library Spectrum

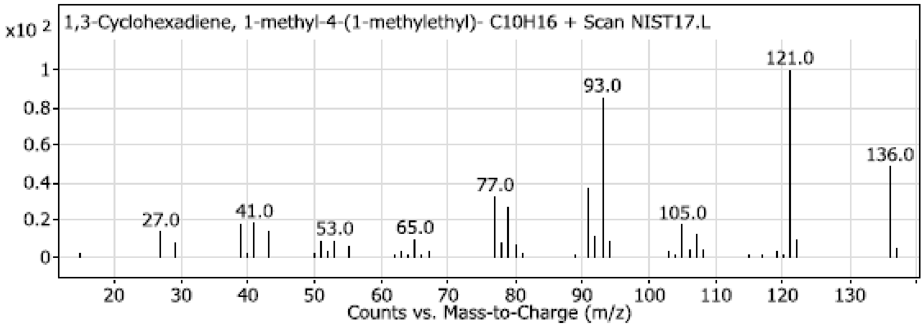

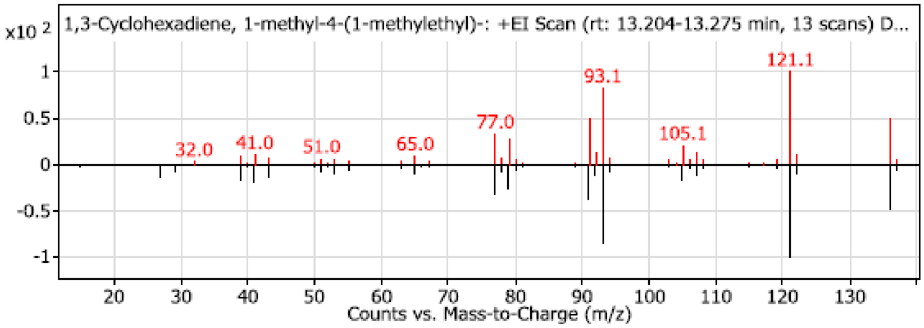

Spectrum Structure

1,3-Cyclohexadiene, 1-methyl-4-(1-methylethyl)-

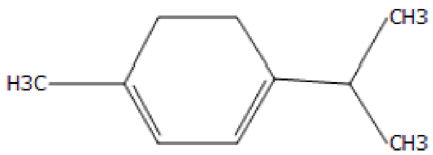

Spectrum Source	Collision Energy	Ionization Mode
Peak (2) in "+ TIC Scan"	0	EI

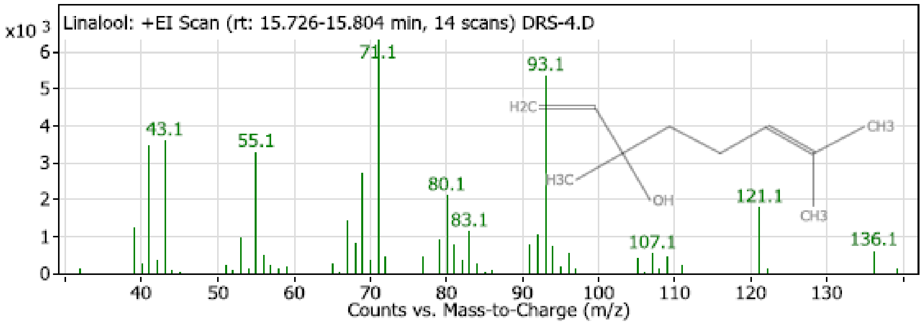

Library Spectrum

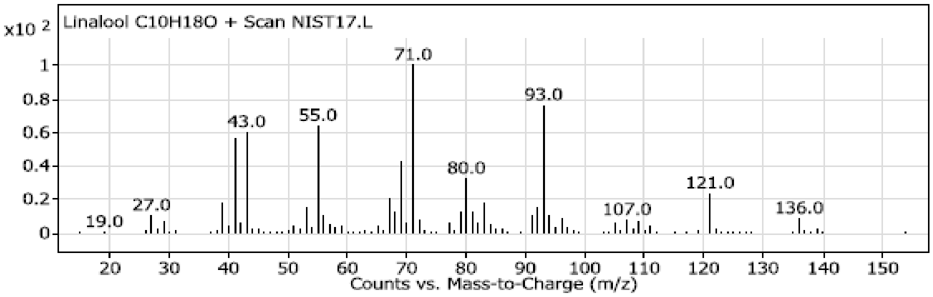

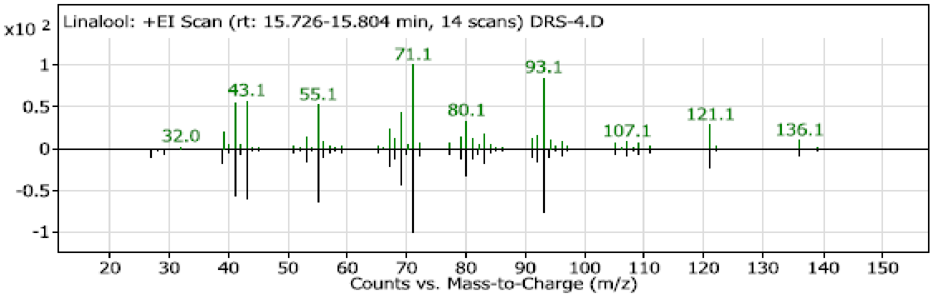

Spectrum Structure

Linalool

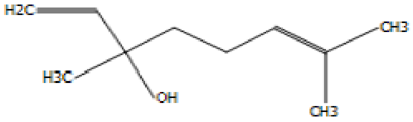

Spectrum Source	Collision Energy	Ionization Mode
Peak (3) in "+ TIC Scan"	0	EI

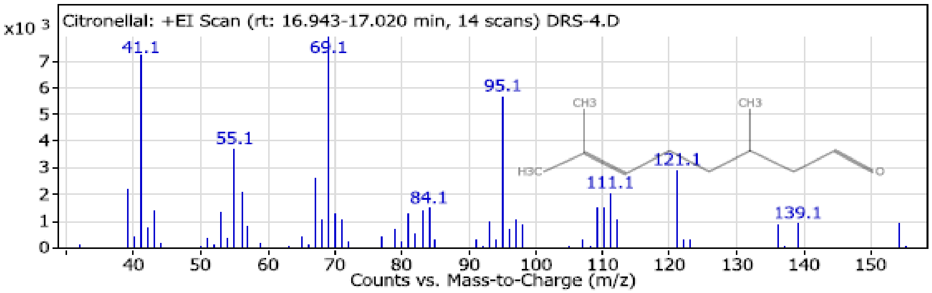

Library Spectrum

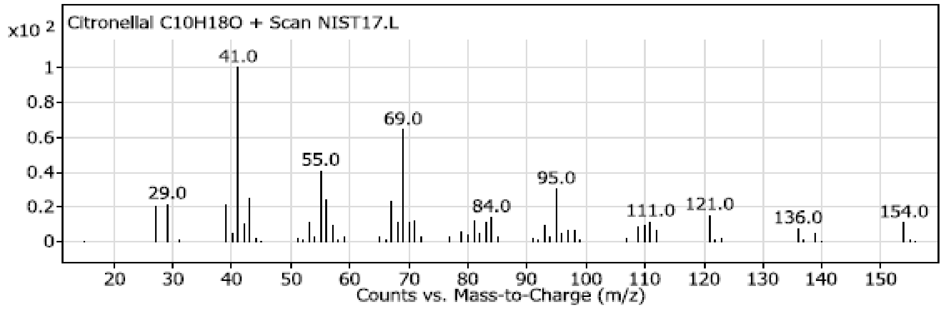

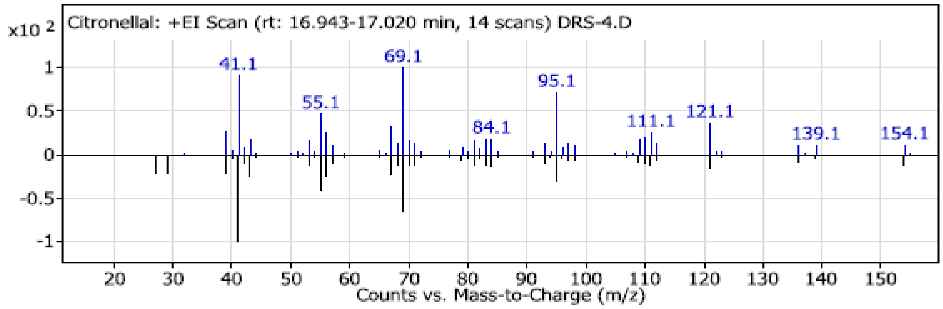

Spectrum Structure

Citronellal

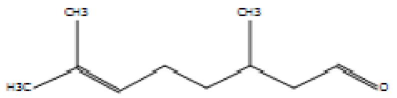

Spectrum Source	Collision Energy	Ionization Mode
Peak (4) in "+ TIC Scan"	0	EI

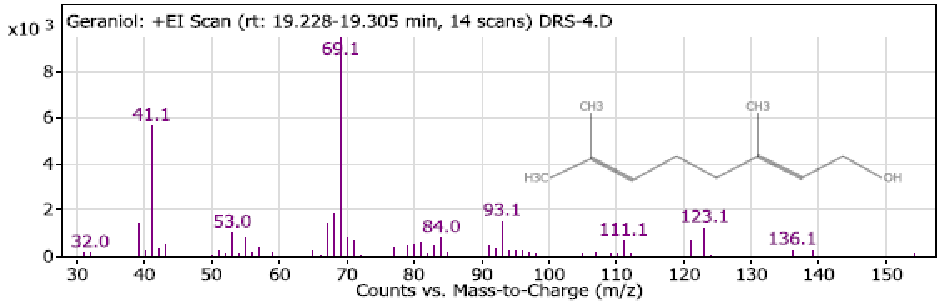

Library Spectrum

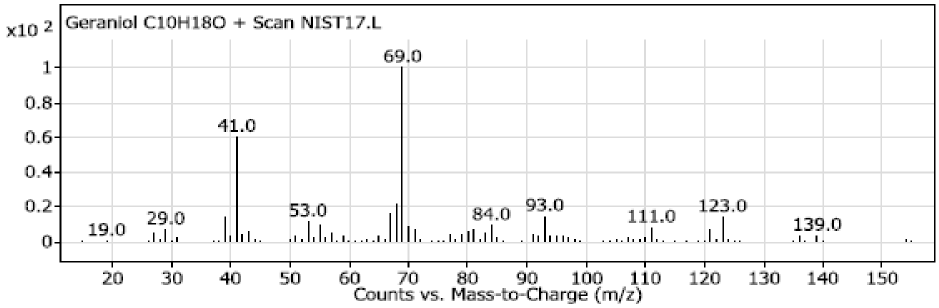

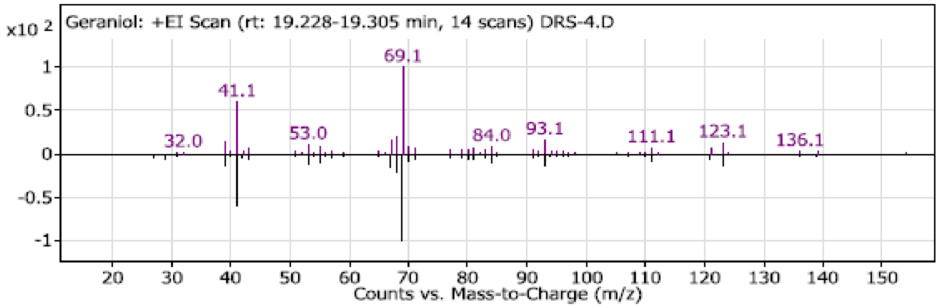

Spectrum Structure

Geraniol

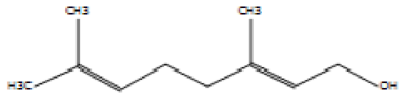

**Spectrum Source**	**Collision Energy**	**Ionization Mode**
Peak (5) in "+ TIC Scan"	0	EI

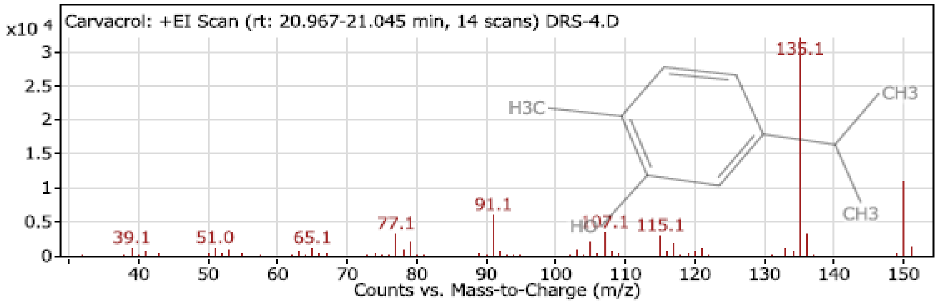

Library Spectrum

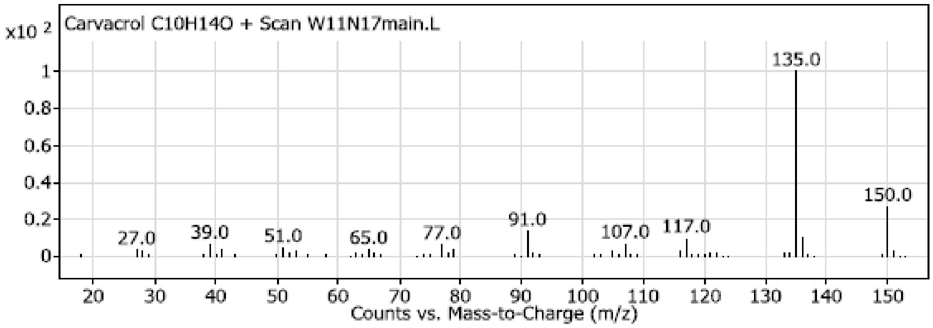

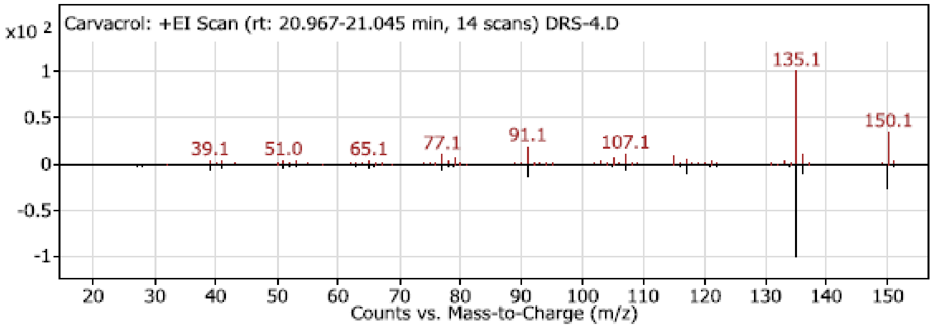

Spectrum Structure

Carvacrol

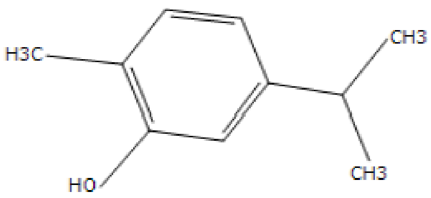

Spectrum Source	Collision Energy	Ionization Mode
Peak (6) in "+ TIC Scan"	0	EI

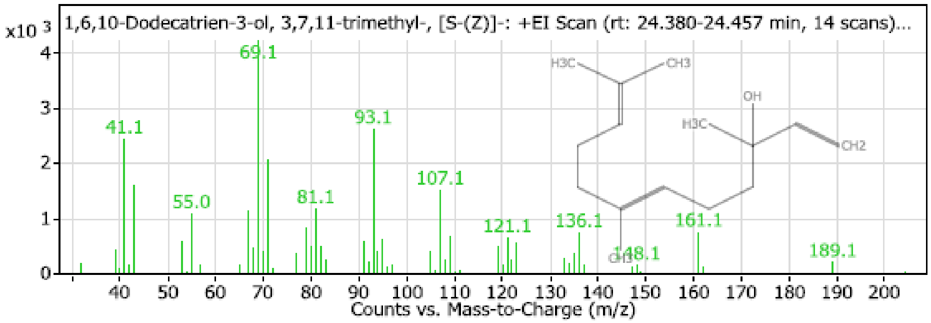

Library Spectrum

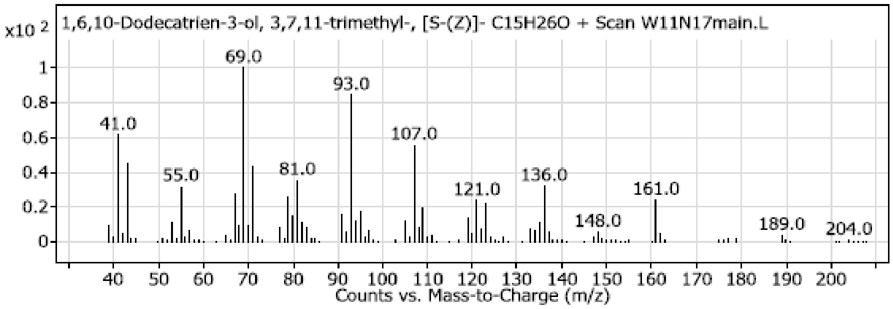

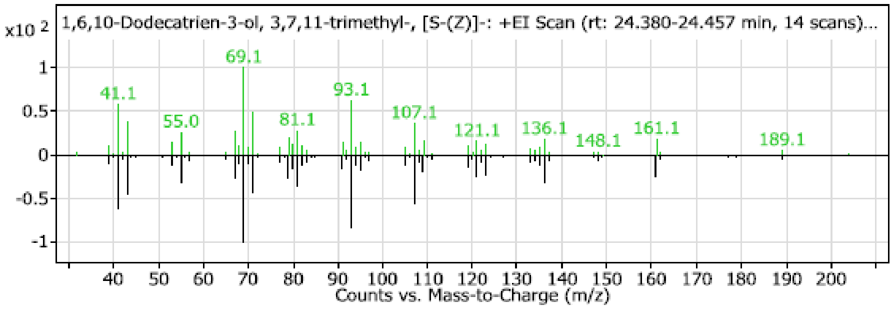

Spectrum Structure

1,6,10-Dodecatrien-3-ol, 3,7,11-trimethyl-, [S-(Z)]-

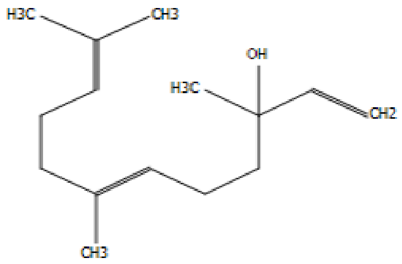

Spectrum Source	Collision Energy	Ionization Mode
Peak (7) in "+ TIC Scan"	0	EI

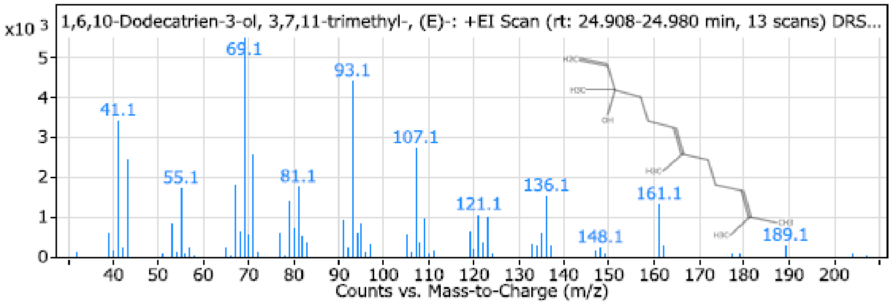

Library Spectrum

Drug Release retention time and peak area after 8hr

Peak	RT	Name	Area	Area Sum %	
1	13.24	1,3-Cyclohexadiene, 1-methyl-4-(1-methylethyl)-	156186	8.94	α-Terpinene
2	15.76	Linalool	234259	13.4	Linalool
3	16.99	Citronellal	322544	18.45	Citronellal
4	19.26	Geraniol	193170	11.05	Geraniol
5	21	Carvacrol	458190	26.21	Carvacrol
6	24.42	1,6,10-Dodecatrien-3-ol, 3,7,11-trimethyl-, [S-(Z)]-	157785	9.03	cis-Nerolidol
7	24.94	1,6,10-Dodecatrien-3-ol, 3,7,11-trimethyl-, (E)-	225823	12.92	trans-Nerolidol

DRS-(10hr)

Qualitative Analysis Report

**Data Filename**	DRS-5.D	**Sample Name**	DRS-5
**Sample Type**		**Position**	4
**Instrument Name**	Head Space	**User Name**	
**Acq Method**	Essnestial oil DB-624 (He).M	**Acquired Time**	5/29/2023 4:58:01 PM
**IRM Calibration Status**	Not Applicable	**DA Method**	SignalToNoiseCheckout.m
**Comment**			

**Expected Barcode**		**Sample Amount**	
**Dual Inj Vol**	1	**TuneName**	ATUNE.U
**TunePath**	D:\MassHunter\GCMS\1\5977 \	**TuneDateStamp**	2023-05-09T16:01:14+02:00
**MSFirmwareVersion**	6.00.34	**OperatorName**	
**RunCompletedFlag**	True	**Acquisition SW Version**	MassHunter GC/MS Acquisition 10.0.368 14-Feb-2019 Copyright © 1989-2018 Agilent Technologies, Inc

User Chromatograms

Fragmentor Voltage **Collision Energy** 0 **Ionization Mode** EI

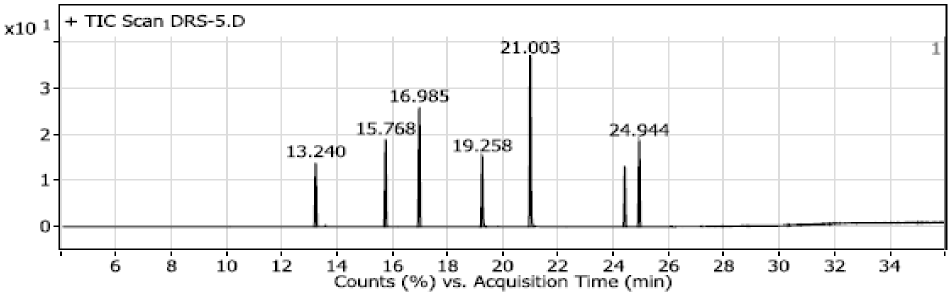

Integration Peak List

Peak	Start	RT	End	Height	Area	Area %
1	13.168	13.24	13.299	58040.19	161242.66	35.69
2	15.694	15.768	15.827	78842.62	228830.51	50.65
3	16.919	16.985	17.05	108502.48	317841.42	70.35
4	19.199	19.258	19.43	63379.53	186955.34	41.38
5	20.938	21.003	21.14	156049.73	451773.44	100
6	24.362	24.416	24.481	55132.2	157610.94	34.89
7	24.876	24.944	25.009	77404.51	226142.94	50.06

User Spectra

**Spectrum Source**	**Collision Energy**	**Ionization Mode**
Peak (1) in "+ TIC Scan"	0	EI

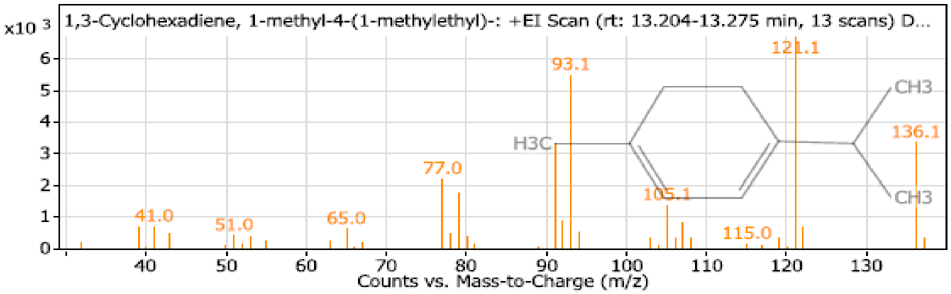

Library Spectrum

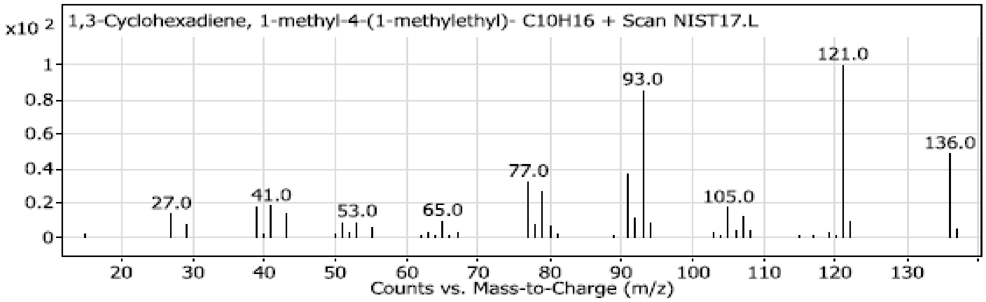

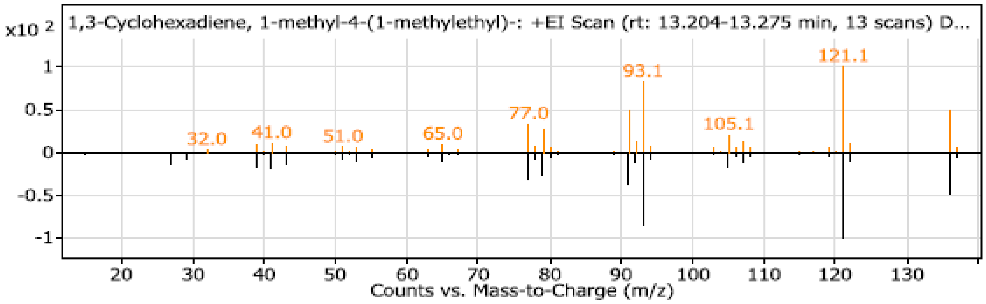

Spectrum Structure

1,3-Cyclohexadiene, 1-methyl-4-(1-methylethyl)-

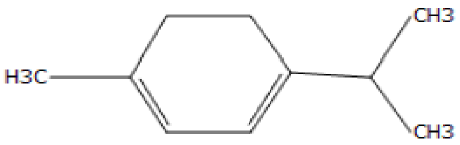

**Spectrum Source**	**Collision Energy**	**Ionization Mode**
Peak (2) in "+ TIC Scan"	0	EI

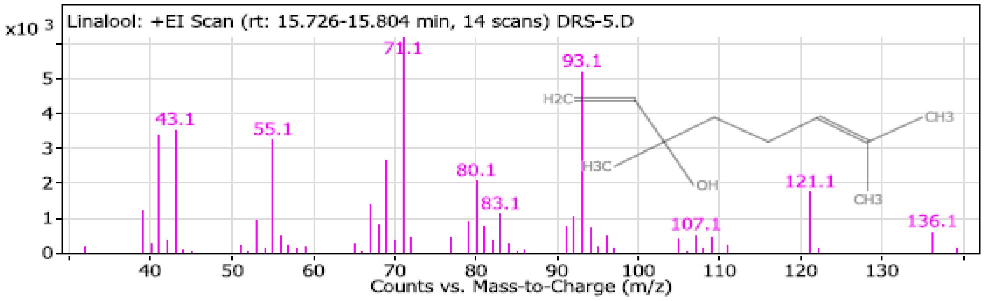

Library Spectrum

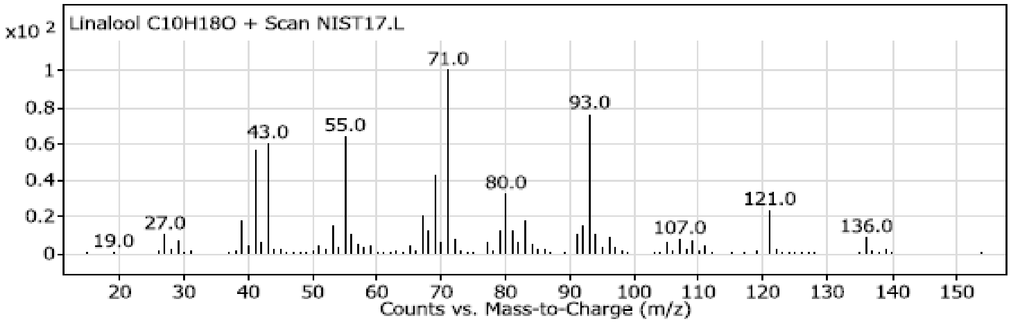

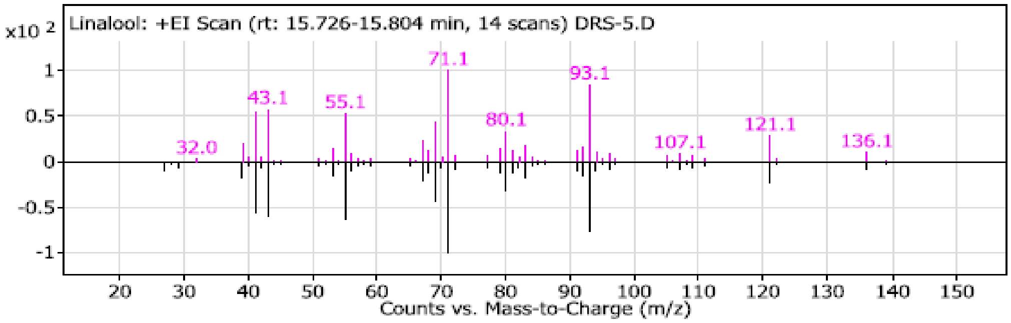

Spectrum Structure

Linalool

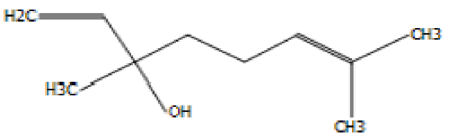

Spectrum Source	Collision Energy	Ionization Mode
Peak (3) in "+ TIC Scan"	0	EI

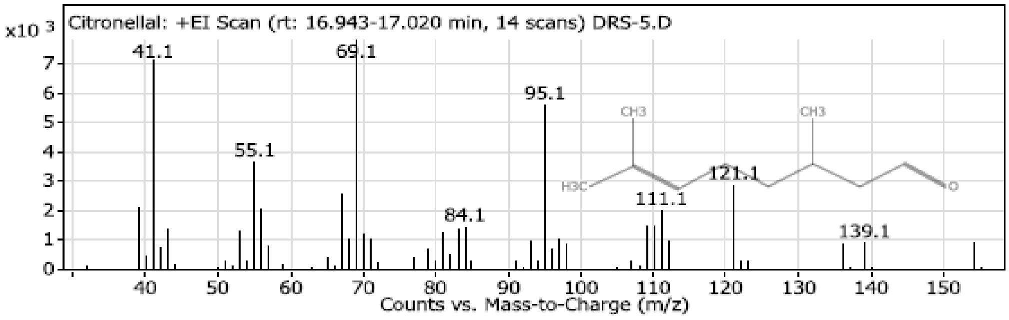

Library Spectrum

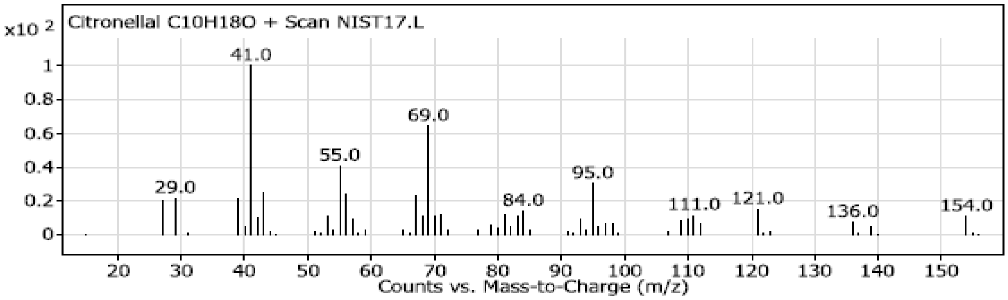

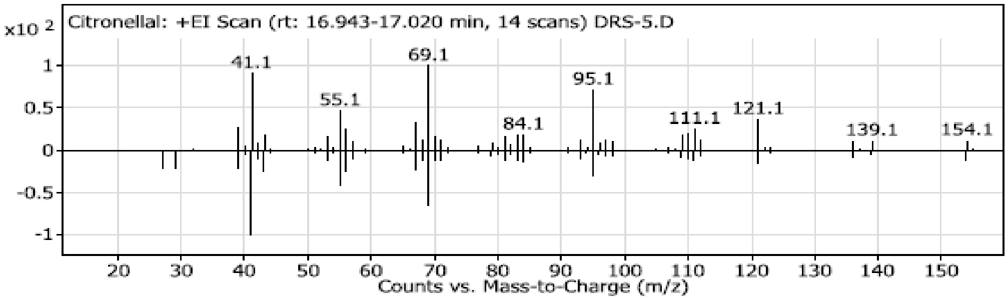

Spectrum Structure

Citronellal

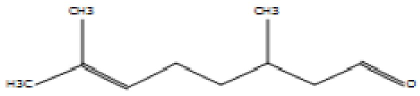

Spectrum Source	Collision Energy	Ionization Mode
Peak (4) in "+ TIC Scan"	0	EI

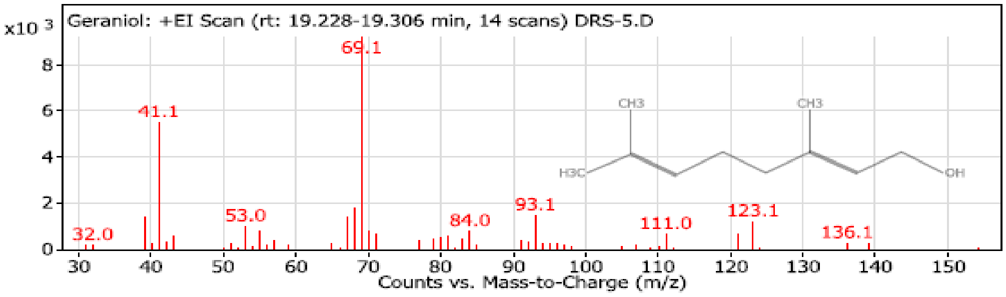

Library Spectrum

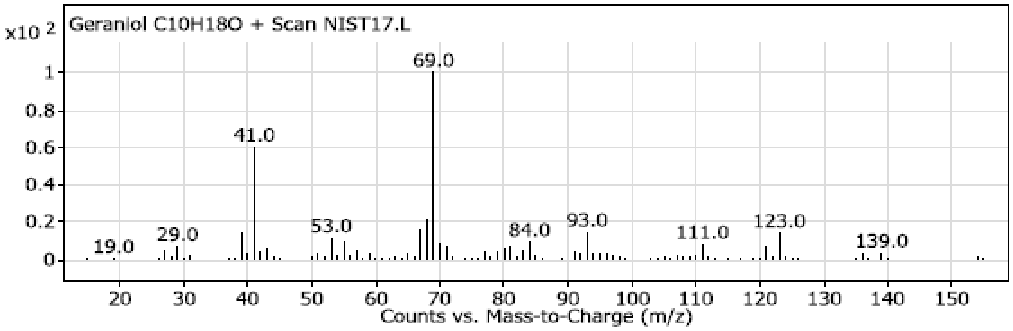

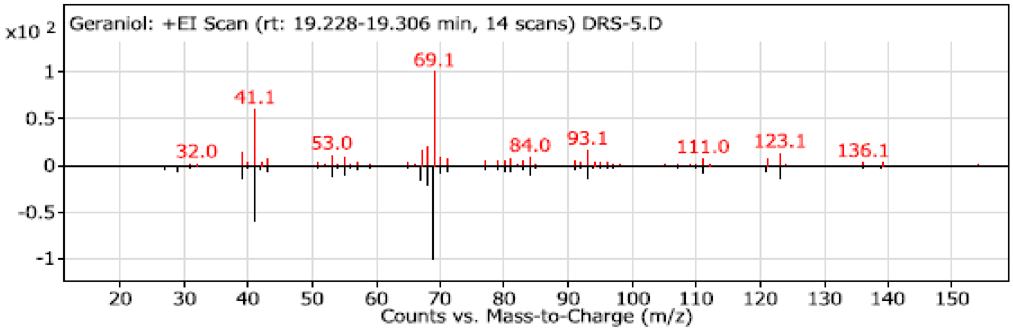

Spectrum Structure

Geraniol

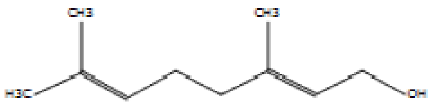

Spectrum Source	Collision Energy	Ionization Mode
Peak (5) in "+ TIC Scan"	0	EI

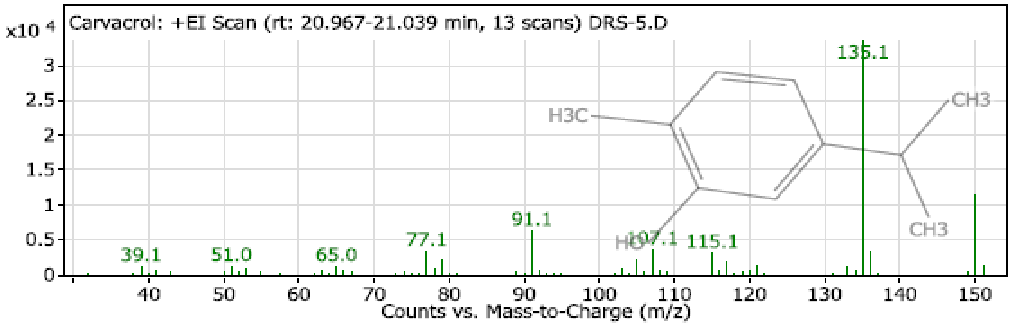

Library Spectrum

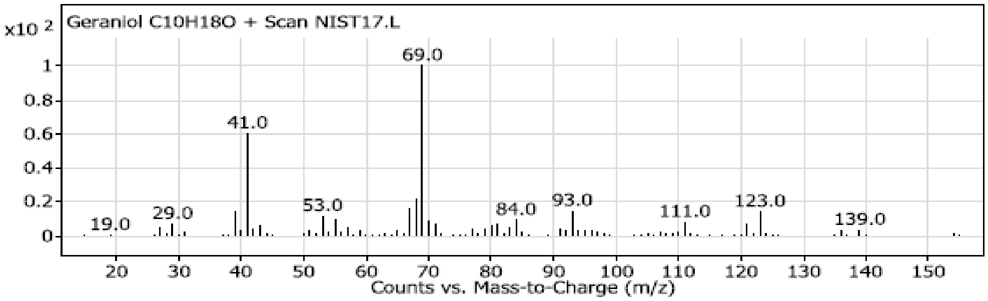

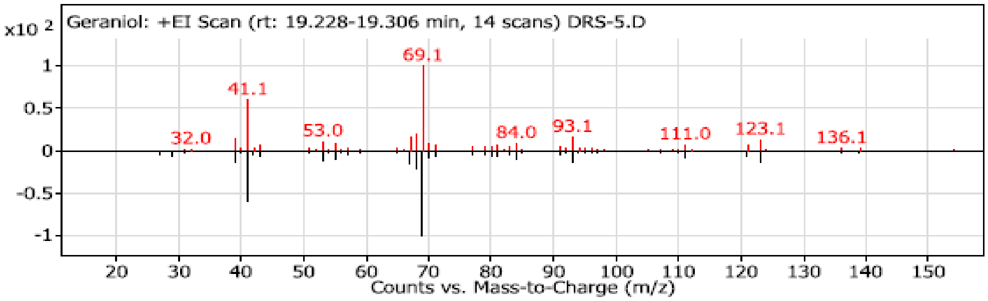

Spectrum Structure

Geraniol

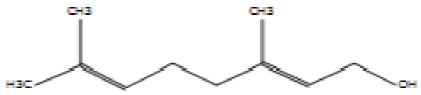

**Spectrum Source**	**Collision Energy**	**Ionization Mode**
Peak (5) in "+ TIC Scan"	0	EI

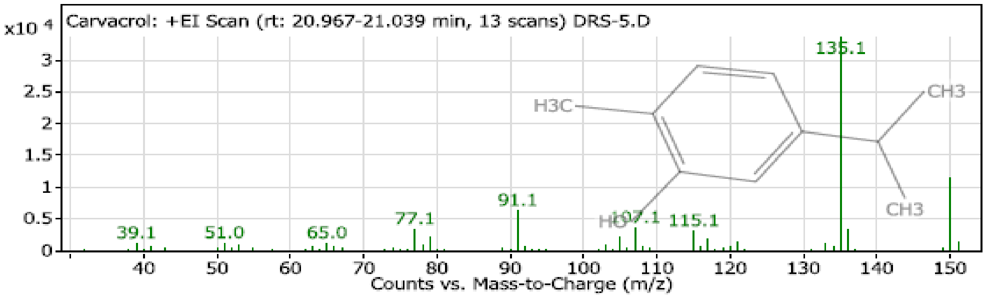

Library Spectrum

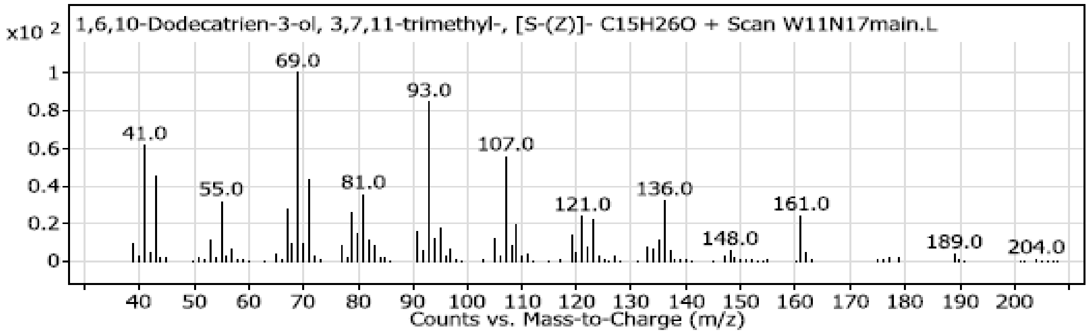

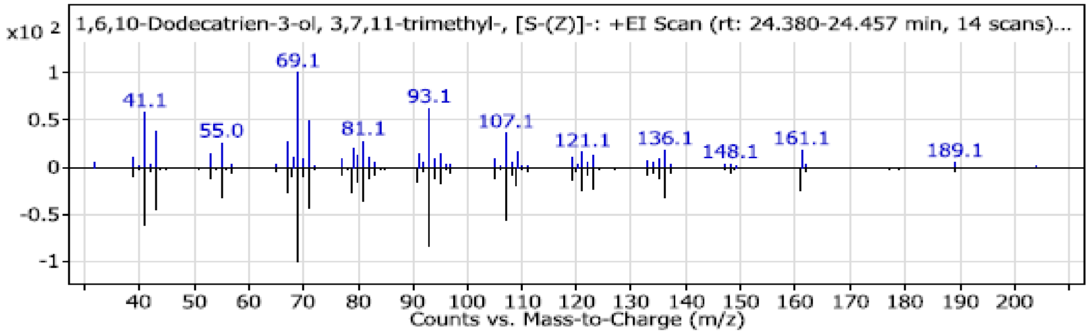

Spectrum Structure

1,6,10-Dodecatrien-3-ol, 3,7,11-trimethyl-, [S-(Z)]-

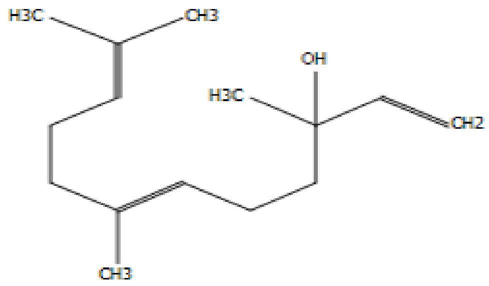

Spectrum Source	Collision Energy	Ionization Mode
Peak (7) in "+ TIC Scan"	0	EI

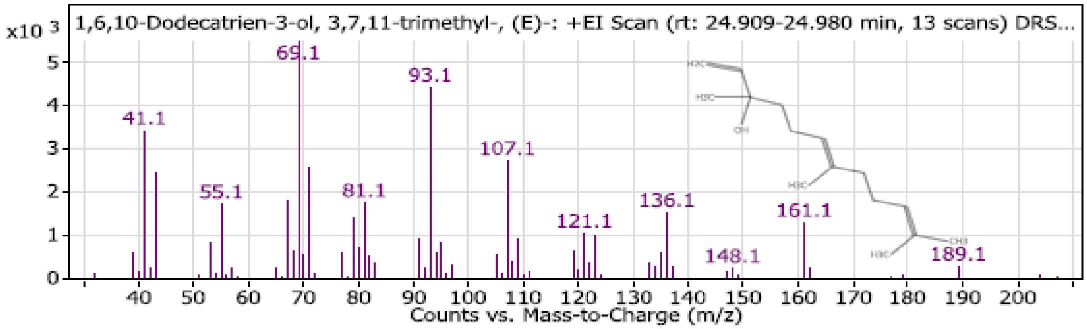

Library Spectrum

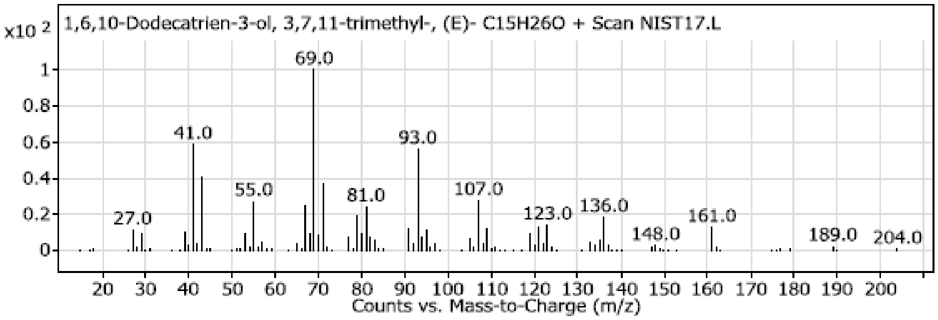

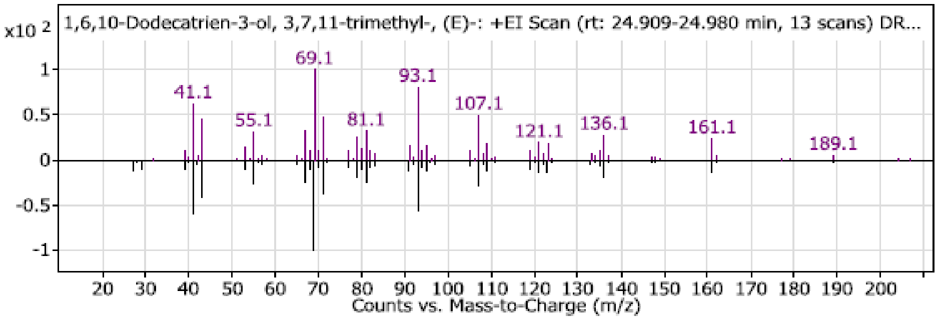

Spectrum Structure

1,6,10-Dodecatrien-3-ol, 3,7,11-trimethyl-, (E)-

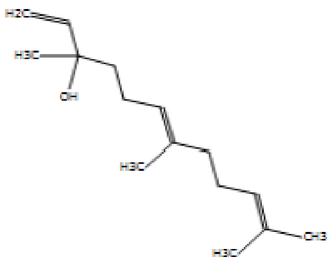

--- End Of Report ---

Drug Release retention time and peak area after 8hr

Peak	RT	Name	Area	Area Sum %	
1	13.24	1,3-Cyclohexadiene, 1-methyl-4-(1-methylethyl)-	161243	9.32	α-Terpinene
2	15.77	Linalool	228831	13.22	Linalool
3	16.99	Citronellal	317841	18.37	Citronellal
4	19.26	Geraniol	186955	10.8	Geraniol
5	21	Carvacrol	451773	26.11	Carvacrol
6	24.42	1,6,10-Dodecatrien-3-ol, 3,7,11-trimethyl-, [S-(Z)]-	157611	9.11	cis-Nerolidol
7	24.94	1,6,10-Dodecatrien-3-ol, 3,7,11-trimethyl-, (E)-	226143	13.07	trans-Nerolidol

STD		
Compund	Conc. (mg/ml)	Area
α-Terpinene	7.533	1541316.38
Linallol	8.3517	1497195.3
Citronellal	7.998	1359477.83
Geraniol	8.692	1436341.25
Carvacrol	9.272	2078204.8
Nerolidol	8.439	2449632.17

DRS-1		
Compund	Area	Conc. (mg/ml)
α-Terpinene	195193.74	95.40
Linallol	213206.8	118.93
Citronellal	324678.91	191.01
Geraniol	158403.54	95.86
Carvacrol	414874.08	185.10
Nerolidol	319676.73	110.13

DRS-2		
Compund	Area	Conc. (mg/ml)
α-Terpinene	135805.22	66.37
Linallol	179492.31	100.12
Citronellal	262069.01	154.18
Geraniol	129645.67	78.45
Carvacrol	340894.07	152.09
Nerolidol	264859.44	91.24

DRS-3		
Compund	Area	Conc. (mg/ml)
α-Terpinene	181159.19	88.54
Linallol	240873.77	134.36
Citronellal	346654.07	203.94
Geraniol	185151.64	112.04
Carvacrol	466466.67	208.12
Nerolidol	376106.11	129.57

DRS-4		
Compund	Area	Conc. (mg/ml)
α-Terpinene	156186.39	76.33
Linallol	234258.93	130.68
Citronellal	322544.01	189.76
Geraniol	193169.99	116.90
Carvacrol	458190.42	204.42
Nerolidol	383607.59	132.15

DRS-5		
Compund	Area	Conc. (mg/ml)
α-Terpinene	30226.28	14.77
Linallol	81546.29	45.49
Citronellal	102682.45	60.41
Geraniol	110643.91	66.96

Carvacrol	155060.11	69.18
Nerolidol	109231.44	37.63
